# N-Glycans modulate tilting of HIV-1 envelope glycoprotein

**DOI:** 10.1038/s41467-026-71874-2

**Published:** 2026-04-15

**Authors:** Mohamed Shehata, Lorenzo Casalino, Madeleine Duquette, Siyu Chen, Alex Flaherty, Patrick M. Waller, Elizabeth Villa, Rommie E. Amaro

**Affiliations:** 1 Department of Molecular Biology, University of California San Diego, La Jolla, CA USA; 2 Howard Hughes Medical Institute, University of California San Diego, La Jolla, CA USA

**Keywords:** Glycobiology, Computational biophysics, Molecular modelling, Structural biology, Biophysics

## Abstract

Human immunodeficiency virus-1 (HIV-1) remains a global health crisis, with over 40 million people living with the virus and no effective vaccine available. Central to HIV infection and immune evasion is the envelope glycoprotein (Env), a heavily glycosylated class I fusion protein that mediates viral entry and is the sole immunogenic target. Despite the recent advancements provided by imaging techniques, the characterization of Env’s structure and dynamics within its native membrane environment remains incomplete. Here, we present microsecond-long, all-atom molecular dynamics simulations of the full-length, glycosylated Env glycoprotein embedded in a biologically relevant lipid bilayer. Our simulations, corroborated by cryo-electron tomography, reveal a pronounced tilting motion of Env relative to the membrane. Importantly, we identify a critical role for N-linked glycans at N88 and N611 in modulating the transition to tilted conformations. Alongside illuminating the sites of vulnerability within the glycan shield, the results presented here underscore Env tilting dynamics as a feature that can be leveraged in immunogen design.

## Abstract (web summary)

HIV-1 uses its heavily glycosylated envelope glycoprotein (Env) to enter cells and evade immune response. Here, authors combine molecular dynamics simulations and cryo-electron tomography to show that N-glycans drive Env tilting, increasing MPER epitope exposure.

## Introduction

Human immunodeficiency virus-1 (HIV-1) remains a global health crisis, having caused between 37.6 and 53.4 million deaths, with over 40 million people currently living with HIV-1 worldwide^1^. The ongoing lack of an effective vaccine continues to hinder global efforts to control the epidemic, which sees approximately 1.5 million new infections annually^1^. At the core of HIV research efforts is the envelope glycoprotein (Env), which mediates viral entry into host cells and serves as the sole viral target exposed on the virion surface for broadly neutralizing antibodies (bNAbs)^2,3^. During viral assembly, Env is expressed as a trimeric gp160, which is subsequently cleaved to become a trimer of gp120-gp41 heterodimers. The gp120 subunit is responsible for binding to the host cell’s CD4 receptor and chemokine coreceptors (CCR5 or CXCR4), while gp41 primes membrane fusion^2,4,5^. Each Env protomer is tethered to the viral envelope via the membrane-proximal external region (MPER), which connects the ectodomain to a single-pass transmembrane domain (TMD) and an intracellular cytoplasmic tail  domain (CTD)^6,7^. Similar to other class I viral fusion glycoproteins^8^, Env is extensively glycosylated^9–14^, with glycans accounting for more than half of its total mass^12,15,16^. Glycans are essential for Env’s folding, stability, and dynamics^16–19^, while also forming a glycan shield that masks underlying proteinaceous epitopes and allows the virus to thwart the host’s immune response^13,16,20,21^. Over the past decade, mass spectrometry-based methods have driven significant progress in the study of the HIV-1 Env glycan shield^9–14^. However, the presence of 23–30 potential N-linked glycosylation sites (PNGS) on the gp160 monomer with remarkable microheterogeneity at each site, the glycans’ inherent flexibility, and the vast sequence diversity among circulating viral strains pose significant hurdles for the structural and functional characterization of the glycan shield^10^. These obstacles are compounded by persistent challenges associated with resolving Env’s three-dimensional structure. Low expression levels and poor long-term stability of native Env, coupled with its structural plasticity, have steered most structural research efforts toward the routine use of engineered, truncated versions of the trimer ectodomain, known as SOSIP trimers^22^. This approach has dramatically facilitated the acquisition of high-resolution structures of the Env ectodomain and provided detailed insights into its conformational dynamics^13,23–31^. However, a comprehensive understanding of Env structure in its native viral membrane context has remained elusive^6^.

Obtaining high-resolution structures of the MPER, TMD, and CTD is challenging due to their proximity to the viral membrane^32^. The structures of these regions have only been resolved in isolation from the ectodomain using nuclear magnetic resonance (NMR)^32–37^. Nonetheless, the majority of current knowledge about Env’s in situ structural plasticity has been primarily derived from studies employing cryo-electron tomography (cryo-ET) and cryo-electron microscopy (cryo-EM)^6,7,38^. For example, Prasad et al.^6^ reported Env tilting on the native membrane in an unliganded state using cryo-ET. Similarly, Env tilting was also observed upon interaction with MPER-directed antibodies, as demonstrated via cryo-EM^7^. In the same study, the authors refer to Env tilting as a part of the Env neutralization mechanism for such antibodies^7,39^. While these studies shed light on Env’s in situ dynamics, their resolution constraints hinder the ability to capture fine structural details. Key aspects, such as the molecular mechanism underlying large conformational transitions (including tilting relative to the membrane) and the dynamics of the glycan shield, cannot be adequately captured by current imaging techniques. To overcome these limitations, molecular dynamics (MD) simulations provide high-resolution, atomic-level insights, enabling detailed characterization of conformational changes closely tied to key functional processes or immunogenic properties. In this regard, MD simulations have been successfully used to investigate the dynamics of class I fusion glycoproteins, either in situ or embedded in a native membrane, for SARS-CoV-2 spike^40–46^ and influenza hemagglutinin^47,48^. On the other hand, computational research addressing Env dynamics remains relatively sparse and underexplored. Some studies have examined the dynamics of the truncated membrane-interacting regions (TMD^49^ or MPER–TMD^37^ without the ectodomain) or a single gp120 monomer with^18^ or without glycans^50^. Other studies have focused on the ectodomain trimer with a simplified glycosylation profile^13,51^, or investigated an unglycosylated full-length model embedded in a nanodisc membrane through coarse-grained simulations^38^. More recently, simulations of a glycosylated ectodomain trimer have been employed to study large-scale conformational changes in HIV-1 Env^52^. This narrow range of studies further underscores the need to bridge the critical gap in understanding the dynamics of the full-length HIV-1 Env in its native membrane environment.

**Fig. 1:**
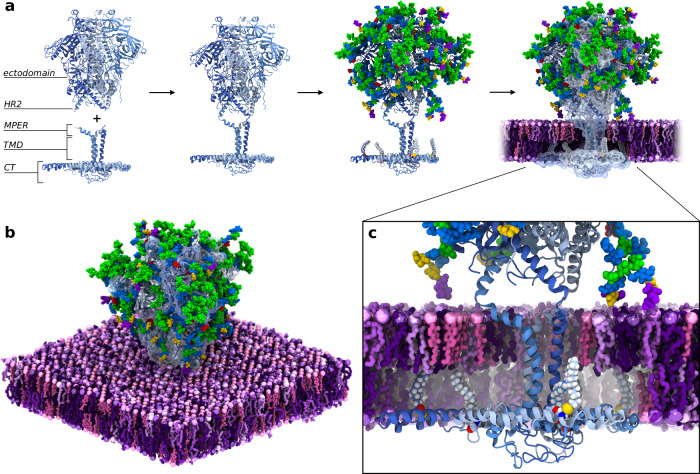
Modeling and simulations of the full-length, glycosylated HIV-1 Env trimer in the closed state. **a** Workflow of the assembly of the ectodomain, membrane-proximal external region (MPER), transmembrane domain (TMD), and cytoplasmic tail (CT) domain into the full-length, glycosylated model of the HIV-1 Env trimer embedded into an all-atom membrane bilayer mimicking the composition of the viral budding site. The protein is depicted with light blue cartoons, with each protomer differentiated by distinct shades. N-glycans are shown in van der Waals (vdW) representation: GlcNAc in blue, mannose in green, fucose in red, galactose in yellow, and sialic acid in purple. Lipid headgroups are depicted as vdW spheres, with shades of purple (from darker to lighter) representing DPPC, POPE, POPS, PSM, and POPC, and pink indicating CHOL. Lipid tails are shown in licorice representation. **b** Oblique view of the fully assembled, glycosylated Env trimer embedded into an all-atom membrane bilayer. **c** Close-up view of the Env’s membrane-interacting region, where the palmitoylated cysteines within the CT are depicted with vdW spheres.

Here, we present µs-long, all-atom, explicitly solvated MD simulations of the full-length Env glycoprotein in the closed state embedded in a native-like membrane lipid bilayer, with a glycosylation profile consistent with glycoanalytic data (Fig. 1). Our simulations reveal that Env undergoes a pronounced tilting motion relative to the membrane normal, exhibiting extraordinary flexibility that is integral to its activity. Consistent with MD simulations, structural characterization of Env expressed in enveloped virus-like particles (eVLPs) using cryo-ET further demonstrates Env’s intrinsic dynamic nature, highlighting its ability to adopt multiple orientations relative to the viral membrane.

Beyond their canonical role in shielding, our simulations reveal that the N88 and N611 glycans interact persistently with the membrane, anchoring Env in tilted conformations and thereby increasing its propensity to tilt. Removal of these glycans in our simulations reduces the extent of Env tilting, underscoring their role in modulating such a transition. Driven by coupled hinging motions, MPER and TMD adopt a diverse set of conformations pivotal to accommodate Env tilting, with the most tilted states showing increased accessibility of the MPER epitope. Finally, we provide an atomically detailed view of the Env’s glycan shield over microsecond time scales, illuminating the sites of vulnerability to neutralizing antibodies. Overall, the simulations and the tomographic data presented here enhance and expand the existing structural and biological data, offering a previously unexplored, atomic-level perspective on the full-length Env glycoprotein’s structure and dynamics in a native-like membrane environment. The identified glycans play dual roles as both shielding devices and functional modifications integral to Env’s conformational changes. Our findings could suggest a potential strategy to modulate Env’s tilting dynamics relative to the membrane, offering mechanistic insights relevant to immunogen design.

## Results

### N-Glycans at N88 and N611 modulate Env tilting cooperatively

In this work, we constructed and simulated a full-length, glycosylated model of the HIV-1 Env trimer. The model was built in three different steps, as fully detailed in the Methods section and illustrated in Fig. 1a, b. The model includes the ectodomain region (residues 31–662), based on the BG505 SOSIP.664 X-ray structure (PDB ID: 5T3Z^23^), and the membrane-interacting region (residues 663–856, comprising MPER, TMD, and CT), modeled from the NMR structure with PDB ID: 7LOI^37^. We note that stabilizing mutations specific to the SOSIP design were reverted to reflect the native sequence of clade A (BG505) Env. Residue numbering referenced throughout this work aligns with the HXB2 sequence, as reported in Supplementary Fig. 1. The resulting construct was glycosylated using an asymmetric, site-specific glycoprofile consistent with glycoanalytic data (see Supplementary Tables 1–3 for composition)^9–11^. Notably, although gp41 contains four consensus PNGS^10,53^, two of them (N618 and N625) were not glycosylated in our model. These sites lack direct experimental evidence for occupancy in the full-length clade A BG505 viral Env^53^, whereas they are glycosylated in Env from other HIV-1 clades and in some membrane-associated ΔCT constructs^53^. As a result, the present simulations are specific to clade A BG505 viral Env and do not capture potential contributions of N618 and N625 glycans. Following cysteine palmitoylation within the CT (Fig. 1a-c), the resulting model was embedded into an all-atom membrane bilayer mimicking the composition of the viral budding site (see Supplementary Table 4).

**Fig. 2:**
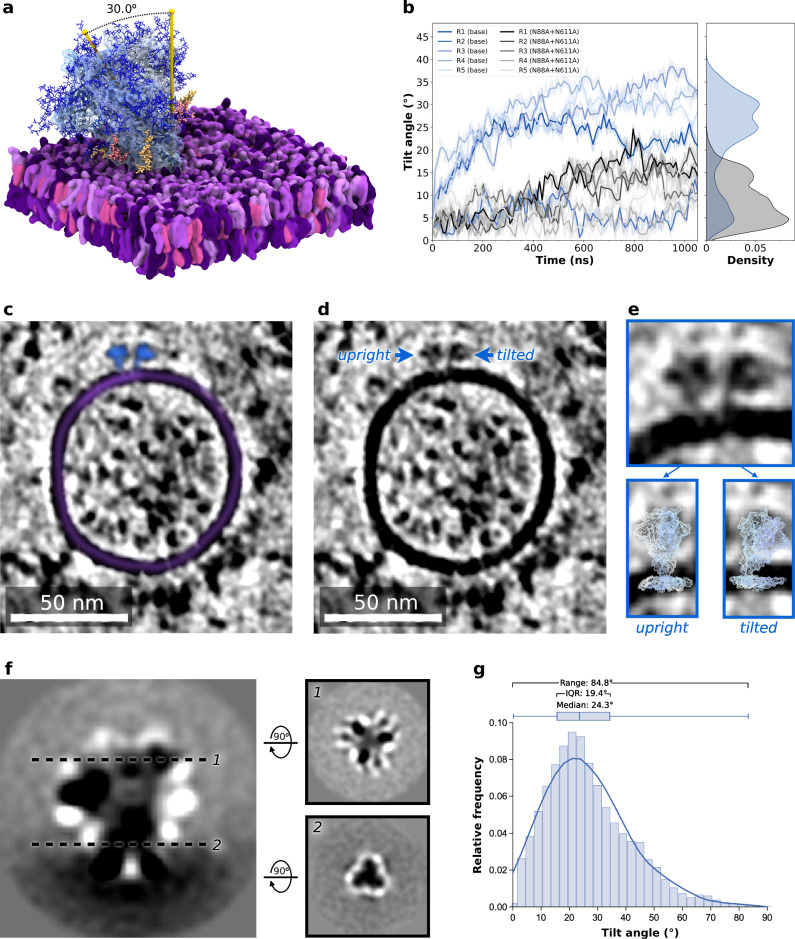
MD simulations and Cryo-ET reveal pronounced Env tilting motion. **a** A representative snapshot of the Env protein showing significant tilting motion relative to the membrane. The Env is displayed with a transparent blue representation, the membrane with a purple surface, and N-glycans are shown with thin blue sticks. N-glycans N88 and N611 are colored in yellow and red, respectively. A tilt angle of ~30.0° is indicated by drawn yellow cylinders. **b** The plot on the left displays the time evolution (x-axis, ns) of the Env tilt angle (y-axis, degree) across the five independent MD simulation replicates for both the base system (blue lines) and the N88A + N611A mutant (black lines). The plot on the right shows the kernel density distribution (x-axis) of Env tilt angles (y-axis, degree). **c** Deconvoluted tomogram slice of a virus-like particle showing Env (blue) coating the membrane surface (purple). **d** Same tomogram slice as (**c**) with representative upright and tilted Envs indicated. **e** Top: Inlay is a closer look at two Envs. An upright Env is on the left and a tilted Env is on the right. Bottom: Upright and tilted conformations of Env extracted from MD simulations are overlayed on the respective upright and tilted Envs shown in the panel above. **f** Subtomogram average density of unliganded Env. The left panel shows a central slice through the averaged density with dashed lines (1 and 2) indicating the positions of the orthogonal sections along the trimer axis, which are displayed on the right. **g** Histogram and box-and-whisker plot of the Env tilt angle distribution from the cryo-ET dataset. Tilt angles were calculated for *n* = 4396 Env subtomogram particles extracted from 239 independent tomograms (565 enveloped virus-like particles). Each data point represents one Env trimer measurement. Values are binned by rounding to the nearest multiple of 3. Range, interquartile range, and median values are shown. Source data are provided as a Source Data file.

We performed µs-long MD simulations to investigate the conformational dynamics of the Env glycoprotein, conducting five independent replicates to ensure robustness and reproducibility (see Supplementary Table 5). Remarkably, our simulations revealed a pronounced tilting motion of the Env ectodomain across multiple replicates (Fig. 2a and Supplementary Movie 1). We quantified the extent of this motion throughout the simulations by calculating the tilt angle of the Env ectodomain relative to the membrane normal (Fig. 2a, b). In four out of five instances, the Env rapidly tilts by ~30°, occasionally reaching tilt angles of ~40° (Fig. 2b). In only one case, the Env oscillates around its initial upright conformation, remaining within the 0–10° range.

To experimentally assess Env’s conformational flexibility, we incorporated Env into eVLPs to preserve its native membrane topology, using the EABR technology described by Hoffmann et al.^54^ to enhance its incorporation efficiency. After purification, the eVLPs were prepared for cryo-ET, and 261 tilt series were collected (see Methods section). The reconstructed tomograms were manually inspected, with each Env identified and marked for subtomogram extraction and subsequent analysis (Fig. 2c–f, Supplementary Fig. 2, Supplementary Table 6). Cryo-ET reveals multiple orientations of Env relative to the membrane, demonstrating its conformational flexibility in vitro (Fig. 2d, e). To calculate the range of motion empirically, the orientation of Env after refinement was used to geometrically determine the tilt angle relative to the normal plane of the eVLP membrane (Supplementary Fig. 3). The distribution of the Env’s tilt angles indicates that unbound Env has an interquartile range of 19.4° and a median tilt angle of 24.3° (Fig. 2g), aligning with our simulations, where most tilted conformations fall within the 20°–30° range (Fig. 2b). Overlay of upright and tilted conformations extracted from MD simulations shows remarkable agreement with Env densities identified from tomogram slices (Fig. 2e). Notably, the distribution of tilt angles determined from cryo-ET subtomogram analysis exhibit a broad distribution peaking around ~25° (Fig. 2g), whereas our simulations reveal a more defined bimodal distribution, with one peak corresponding to the upright state and a predominant peak for the tilted state (Fig. 2b). The difference likely arises from the inherent limitations of conventional MD simulations, where the accessible timescales restrict the number of observed tilting events. In our simulations, Env typically undergoes a single tilting event that progresses quickly without fully returning to the upright (or less tilted) orientation (Fig. 2b). By contrast, cryo-ET captures an ensemble of conformations across many particles, effectively integrating over much longer timescales. As a result, it encompasses multiple independent tilting events, yielding a broader distribution of tilt angles that approximates a normal distribution (Fig. 2g).

Visual inspection of our simulations revealed direct interactions between Env’s N-linked glycans proximal to the gp120-gp41 interface and the lipid membrane (Fig. 2a and Supplementary Movie 1). To quantify these interactions, the contact frequency of each glycan with the membrane was calculated over the course of the simulations. Two glycans, N88 and N611, exhibit the highest contact frequencies with the membrane, with N88 at 58.1% and N611 at 67.0% (Supplementary Fig. 4). No notable contact frequencies are observed for the remaining glycans; however, interactions with the membrane increase for some of these glycans toward the end of the simulation as a result of Env tilting (Supplementary Table 7). Given the high contact frequency of N88 and N611 glycans, we investigated their conservation among different HIV strains (Supplementary Fig. 5). Interestingly, we found that these two glycans, modeled here as complex bi-/tri-antennary with partial fucosylation and sialylation, are strongly conserved. To assess the contribution of these two glycans to Env’s conformational dynamics, we generated an additional Env model in which we introduced alanine substitutions at positions N88 and N611, effectively deleting the respective N-linked glycans (see Methods section). Remarkably, MD simulations of the mutant demonstrated a substantial reduction in the Env tilt angle (Fig. 2b, Supplementary Movie 2). For most of the simulation time, the mutant system remains within a tilt angle range of 0–10°, more rarely exhibiting slightly more tilted conformations. Altogether, these observations suggest that N88 and N611 glycans play a critical role in modulating Env’s propensity to tilt.

**Fig. 3:**
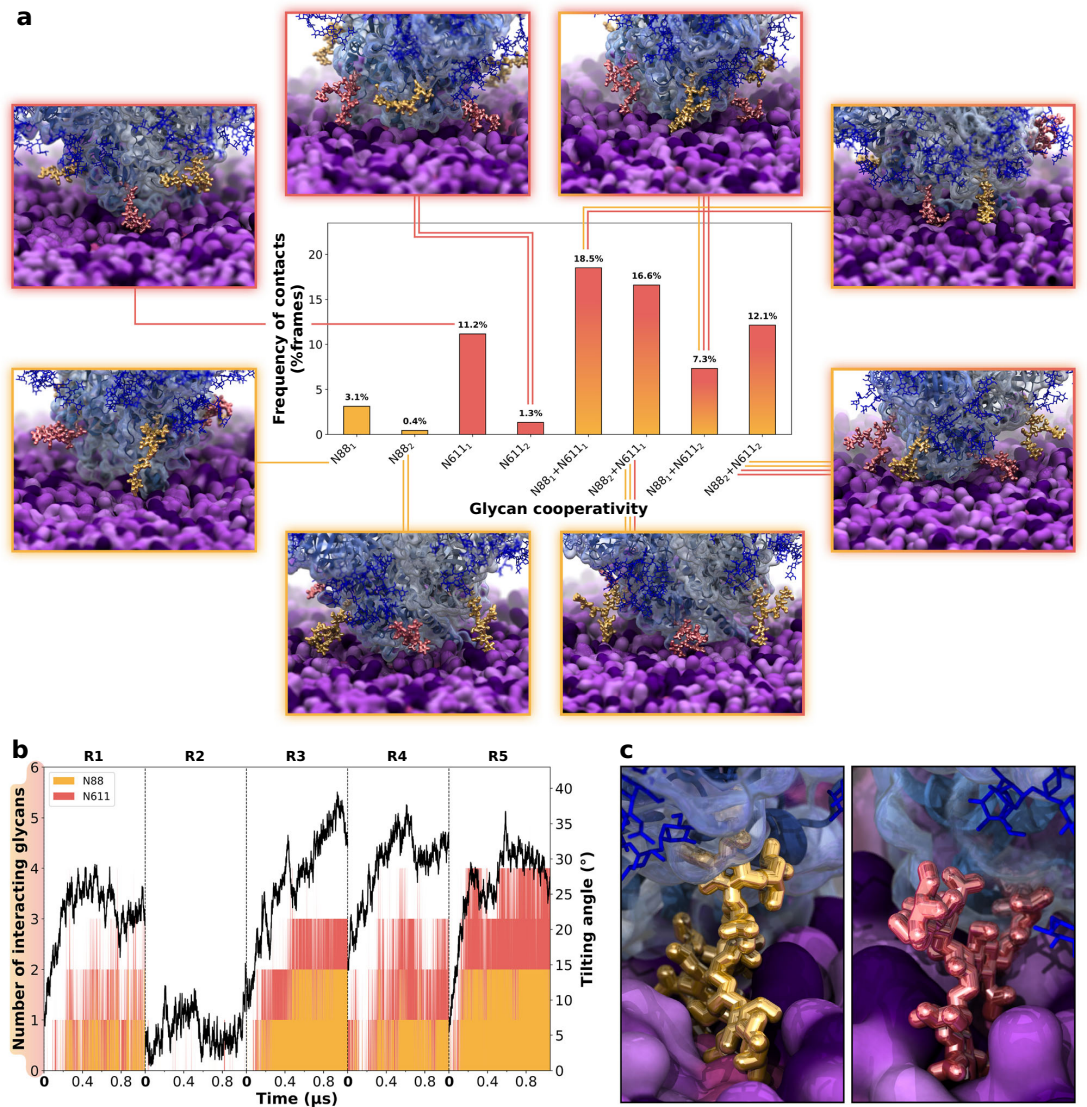
Dissection of interactions between N88/N611 glycans and the viral membrane. **a** The bar plot highlights the frequencies of single (N88_1_ or N611_1_) or combined (N88_2_, N611_2_, N88_1_ + N611_1_, N88_2_ + N611_1_, N88_1_ + N611_2_, N88_2_ + N611_2_) contacts of N88 and N611 glycans with the lipid bilayer accompanied by molecular representations of all interaction combinations surrounding the plot. Contact frequencies were calculated and pooled across all simulations and protomers and, for each category, are expressed as % frames with at least one contact from that category. Env is depicted with a blue surface representation, N88 and N611 glycans are shown with yellow and red sticks, respectively, whereas the membrane is displayed with a purple surface. **b** Time evolution of the number of N88 (yellow lines) and N611 (red lines) glycans interacting with the membrane (left y-axis), along with the corresponding Env tilt angle in degree (black line, right y-axis) across simulation replicas. **c** Molecular representation of glycans N88 (yellow sticks) and N611 (red sticks) inserted into the lipid bilayer, illustrating their anchoring role during Env tilting.

To explore how N88 and N611 glycans mediate Env tilting, we sought to dissect the frequency and nature of their interactions with the membrane (Fig. 3a). Our analysis reveals that these glycans contact the head groups of the lipid molecules in diverse ways: sometimes independently, but more frequently cooperatively, with N88 and N611 simultaneously engaging the membrane. As shown in Fig. 3a, individual interactions exclusively involving only one N88 glycan (N88_1_) or one N611 glycan (N611_1_) exhibit frequencies of 3.1% and 11.2%, respectively, while interactions involving either two N88 glycans or two N611 glycans at the same time are rare (e.g., N88_2_ at 0.4% and N611_2_ at 1.3%). In contrast, for most of the simulation time, N88 and N611 concomitantly interact with the membrane. For example, the combination of one glycan from N88 and one glycan from N611 (N88_1_ + N611_1_) is observed in 18.5% of the frames, the highest frequency among all combinations. Similarly, other cooperative interactions, such as two glycans from N88 and one glycan from N611 (N88_2_ + N611_1_) or one glycan from N88 and two glycans from N611 (N88_1_ + N611_2_), display notable frequencies of 16.6% and 7.3%, respectively. The majority of these contacts are mediated by N-acetylglucosamine (GlcNAc) residues from the N88/N611 glycans and by sphingomyelin (PSM) and phosphatidylcholine (POPC) among the lipids, reflecting their high abundance (Supplementary Fig. 6). In contrast, cholesterol is infrequently contacted despite its high density in the membrane. Overall, these observations emphasize how the synergy between N88 and N611 glycans is integral to the tilting mechanism, possibly affecting the direction and the extent of the transition.

Next, to gain further insights into the mechanism of Env tilting, we tracked the aggregate number of N88/N611 interacting glycans across the three protomers throughout the simulations, with a particular focus on the relationship with Env tilting. As shown in Fig. 3b, at least one glycan from N88 or N611 engages with the lipid bilayer at the onset of Env tilting. As tilting progresses, the number of N88/N611 interacting glycans steadily increases, reaching up to four. Notably, this pattern is absent in simulation R2, where no significant tilting occurs. Furthermore, Fig. 3c provides a molecular visualization of N88 and N611 glycans deeply embedded into the lipid bilayer, suggesting a role as conformational anchors during Env tilting. Transient anchoring, where N88 and N611 insert deeply into the lipid outer leaflet, likely stabilizes the tilted conformation of Env by providing further structural support to the points of contact with the membrane. Altogether, these observations suggest that interactions between N88/N611 glycans and the viral membrane are not only critical for initiating Env tilting but also for stabilizing the tilted conformations.

### Tilting of Env trimer is accommodated by MPER and TMD hinging motions

**Fig. 4:**
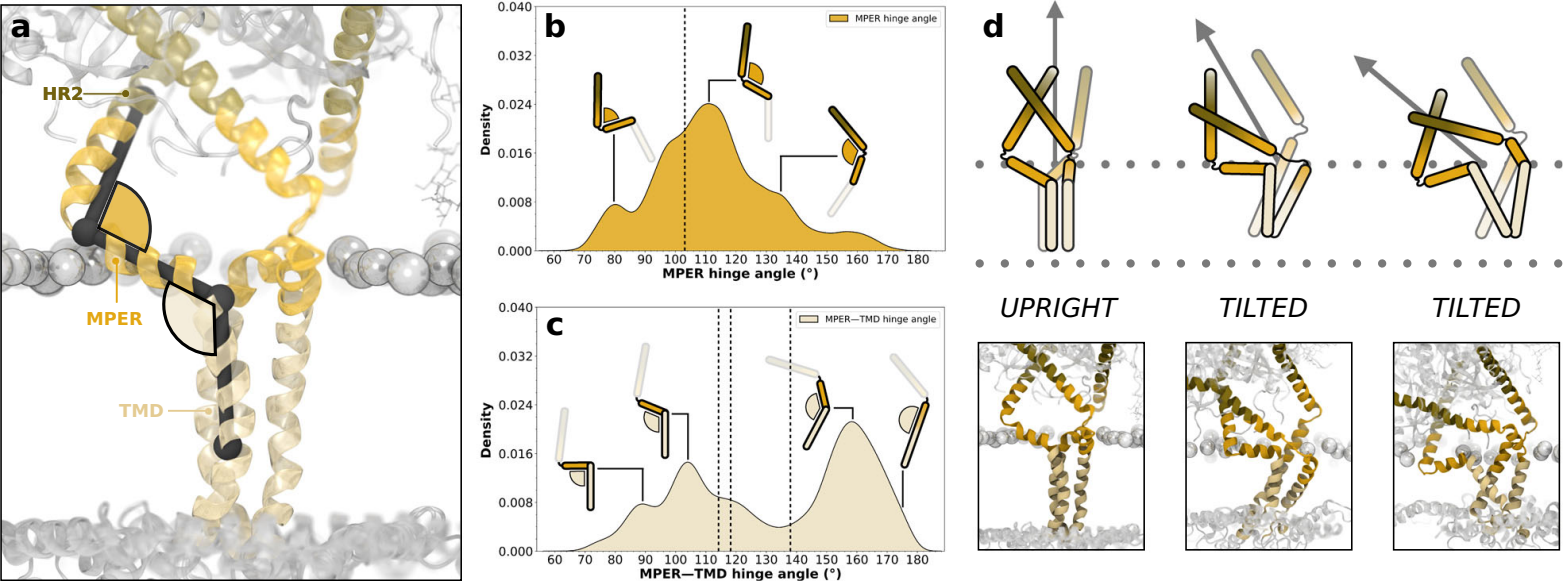
Conformational landscape explored by membrane-proximal external region (MPER) and transmembrane domain (TMD). **a** Schematic representation of the two hinge angles analyzed: MPER hinge angle (orange) and MPER–TMD (beige) hinge angle. **b** Kernel density distribution (y-axis) of the MPER hinge angle (x-axis, degree). **c** Kernel density distribution (y-axis) of the MPER–TMD hinge angle (x-axis, degree). The dashed lines indicate the initial angle values derived from the starting model before simulations. **d** Representative conformations from simulations and relative schematic illustrations showing the upright and tilted states of the Env trimer, highlighting MPER and TMD adaptations as the trimer transitions to tilted orientations relative to the membrane. Source data are provided as a Source Data file.

The MPER (residue 660–683) and the TMD (residue 684–706) are critical structural domains of Env, playing vital roles in membrane anchoring, fusion, viral entry, and structural stabilization of the Env trimer^7,55^. The MPER bears a hinge-like structure, with a linker loop serving as the pivot point between an N-terminal α-helix (spanning the last three helical turns of HR2) and a three-turn C-terminal α-helix positioned at the level of the membrane’s phosphate head groups, together forming a helix–turn–helix motif (Fig. 4a)^56^. This one transitions into the single-pass transmembrane helix, i.e., the TMD, forming an almost continuous helix slightly bent at residue 683 (Fig. 4a). Consequently, this structural organization defines two hinge points, which are illustrated in Fig. 4a: one at the linker loop in the middle of the MPER, hereafter referred to as ‘MPER hinge angle,’ and another at residue 683, marking the transition between the MPER and TMD, hereafter referred to as ‘MPER–TMD hinge angle.’

Using kernel density estimates, we analyzed the distributions of these hinge angles across our simulation replicates to capture the range of conformations sampled (Fig. 4b, c). The distribution of MPER hinge angles shows a broad peak centered around 110°, spanning a range of angle values from 75° to 165° (Fig. 4b). Schematic representations depicted above the distribution plot in Fig. 4b illustrate the most significant conformations sampled by the MPER. At smaller angles ( ~ 85°), the MPER adopts a more tightly bent state. Around the peak of the distribution ( ~ 110°), it maintains a slightly open yet stable configuration, consistent with the initial conformation and preserving the helix–turn–helix architecture. At larger hinge angles ( ~ 140°), the MPER stretches along the linker loop into a more extended state. Differently from the MPER hinge angle, the MPER–TMD hinge angle distribution exhibits a bimodal profile, with peaks at ~105° and ~160°, corresponding to moderately bent and extended conformations where a slight bend persists around residue 683 (Fig. 4c). More compact (~90°) and super-extended (~180°) states, in which the helix is tightly bent or nearly straight, are sampled less frequently (Fig. 4c). Taken together, the observations from both hinge angles indicate that the MPER and TMD preserve their hinge-like architecture with sufficient flexibility to accommodate Env tilting, while structural or energetic constraints limit extreme bending or extension. Remarkably, in the mutant simulations (N88A + N611A), where tilting is reduced, the MPER hinge angle remains narrowly distributed around ~100°, while the MPER–TMD hinge angle persists within the ~110°–140° range, closely reflecting the initial model (Supplementary Fig. 7). This reduced variability compared to the one observed in the base model simulations further supports the hypothesis that hinging motions in the MPER–TMD region are critical structural adaptations required to accommodate Env tilting.

To recapitulate the mechanism by which the MPER and TMD regions accommodate Env tilting, we combined hinge-angle distributions with structural snapshots from simulations representing upright, tilted, and extremely tilted conformations (Fig. 4d). As a result, our observations indicate that the MPER’s and TMD’s concerted transitions between compact, bent, and extended configurations are affected by both the extent of Env tilting and the trimeric spatial arrangement of the protomers. In the upright state (Fig. 4d), represented by our initial model, all three protomers exhibit symmetric hinge angles of ~110° for the MPER and ~110°/ ~ 135° for the MPER–TMD. Upon tilting, asymmetry emerges (Fig. 4d): the protomer in the tilt direction bends further, with MPER hinge angles reduced to ~95°–80° and MPER–TMD hinge angles to ~90°–100°. Conversely, the other two protomers extend, with MPER hinge angles of ~135°–160° and MPER–TMD hinge angles of ~160°–180°. Moreover, in agreement with previous hypotheses suggesting a loosely folded scissoring motion of the TMD helices in their ground state^7^, our simulations reveal a significant mobility of the TMD helices within the membrane. Analysis of the angle of intersection between the TMD helices and the membrane leaflets shows that the TMD can shift from a vertical orientation (~90°) to a tilted orientation (~40°) (Supplementary Fig. 8). Although we do not directly quantify such scissoring motion in more detail, representative snapshots from our simulations demonstrate that the TMD helices orient themselves in a tilted fashion within the lipid bilayer (Fig. 4d, Supplementary Fig. 8), forming a cross-like arrangement with adjacent protomers. We hypothesize that this dynamic reorientation of the TMD helices within the bilayer also plays a key role in accommodating Env tilting.

### Atomic-level overview and analysis of Env’s glycan shield

**Fig. 5:**
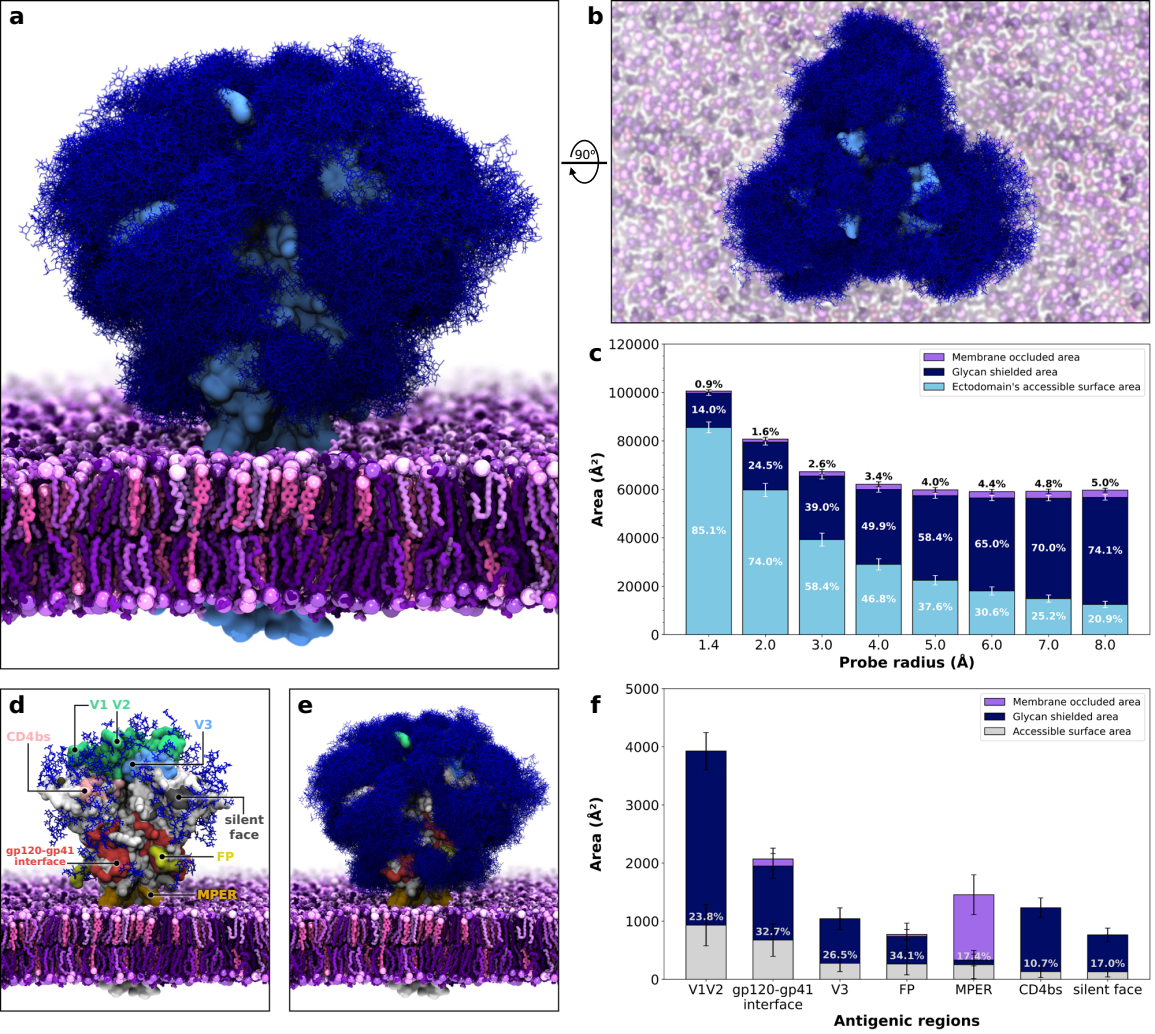
Glycan shield of HIV-1 Env. **a**, **b** Molecular representations of the HIV-1 Env trimer’s glycan shield, shown from a side view (**a**) and a top-down view (**b**). Glycans are shown as a blue mesh ensemble of overlaid conformations sampled during a 1 µs-long simulation. The protein is represented by a cyan surface, while the membrane is depicted in purple, with lipid headgroups represented as van der Waals spheres. **c** Quantification of the accessible surface area (cyan), glycan-shielded area (dark blue), and membrane-occluded area (purple) in Å^2^ across probe radii ranging from 1.4 Å to 8 Å (x-axis), with relative percentages indicated within each stacked bar. For each stacked bar, values were pooled across five independent simulation trajectories (*n* = 5) initiated with distinct velocity seeds. Within each trajectory, surface areas were calculated over 1051 sampled frames. Error bars indicate the standard deviation of the pooled values across all sampled frames. **d** Overview of the HIV-1 Env trimer’s major antigenic regions. The protein is represented with a light gray surface, and the antigenic regions are highlighted in distinct colors: CD4 binding site (CD4bs, pink), V1V2 (green), V3 (cyan), silent face (dark gray), gp120-gp41 interface (red), fusion peptide (FP, yellow), and the membrane-proximal external region (MPER, orange). Glycans are depicted as blue sticks. **e** Alternate representation of the Env trimer as in (**d**), but with glycans shown as a blue mesh ensemble of overlaid conformations sampled during a 1 µs-long simulation. **f** Quantification of the accessible surface area (light gray), glycan-shielded area (dark blue), and membrane-occluded area (purple) for the antigenic regions represented in (**e**, **f**) using a probe radius of 7.2 Å. For each stacked bar, values were pooled across five independent simulation trajectories (*n* = 5) initiated with distinct velocity seeds. Within each trajectory, surface areas were calculated over 1051 sampled frames and three protomers per frame (15,765 total measurements). Error bars indicate the standard deviation of the pooled values across all sampled frames. Source data are provided as a Source Data file.

Albeit many bNAbs require glycan recognition for high-affinity binding, the glycan shield remains one of the most effective strategies used by class I viral fusion glycoproteins—including HIV-1 Env—to thwart the humoral response^16,20,57,58^. Aiming to characterize the glycan shield of Env at the atomic level of detail, we analyzed its spatial distribution and dynamic behavior during our MD simulations. In Fig. 5a, b, we provide previously unappreciated atomic-level views of the glycan shield, revealing its configuration and appearance over the microsecond time scale. Due to their inherent flexibility and multi-antennary, branched structures, N-glycans are poised to rapidly cover a substantial portion of the underlying proteinaceous residues, acting on timescales much shorter than those required for protein conformational changes. We overlaid equally interspersed glycan conformations sampled over a 1 μs-long simulation onto a reference Env conformation, probing the protein for potential sites of vulnerability. The atomic-level representation of the glycan shield reveals that the Env is almost entirely covered, leaving minimal exposure to proteinaceous residues (Fig. 5a, b).

To assess the glycan shield’s effectiveness in occluding the underlying proteinaceous residues, we calculated Env’s accessible surface area (ASA) to spherical probes with radii ranging from 1.4 Å to 8 Å (Fig. 5c, see Methods section for a complete description). A probe with a radius of 1.4 Å is commonly used to describe a water molecule, larger radii (2.0–5.0 Å) are used to represent small molecules, while 7.0–8.0 Å approximate the size of the complementarity-determining regions (CDRs) of an antibody’s variable fragment (Fv)^59^. This analysis demonstrates a striking dependency of ectodomain accessibility on probe size. At the smallest probe radius of 1.4 Å, approximately 85% of the ectodomain remains accessible, reflecting the potential facility of small molecules to penetrate the glycan layer. As the probe radius increases, the extent of glycan shielding becomes gradually more pronounced. For probes with a 3.0 Å radius, accessibility decreases to ~58%. At larger radii (7–8 Å), which approximate the size of an antibody’s CDR, glycans obscure ~70–74% of the ectodomain surface, leaving only ~20–25% accessible (Fig. 5c). These findings highlight the general ability of the glycan shield to function as a dynamic barrier predominantly restricting access to larger molecules, such as antibodies, which bind epitopes on significantly slower timescales (µs to ms) than glycan conformational fluctuations (ps to ns)^40^. Nonetheless, it is important to note that the glycan shield must adapt to allow access to the ectodomain surface during the infection process^60^. Our molecular representation of the glycan shield in Fig. 5a, b shows the presence of multiple vulnerabilities, akin to cracks in the sugary armor. Several antigenic regions on Env have been identified as targets of bNAbs, with epitopes primarily located in the variable regions 1 and 2 (V1/V2)^61–64^, V3^61,65,66^, the CD4 binding site (CD4bs)^67–69^, the silent face^70,71^, the gp120-gp41 interface^25,72,73^, the fusion peptide (FP)^72,74^, and the MPER^56,75–77^ (see Fig. 5d, e and Supplementary Table 8 for a detailed list of bNAbs targeting these regions). To unravel potential sites of vulnerability that could inform immunogen design,^57,58,78^ we measured the extent of occlusion conferred by both the glycan shield and the membrane across several epitopes (Fig. 5f). For this purpose, we only calculated the accessibility to a probe mimicking the size of an antibody’s CDR (radius 7.2 Å). Our findings indicate that the FP is the most accessible antigenic site, with ~34.1% of its surface exposed, followed by the gp120-gp41 interface (32.7%). These regions stand out as the most vulnerable to immune recognition despite the presence of glycans N88 and N611, which participate in the neutralization mechanism required by bNAbs 35O22^73^ and PGT151^72^. Conversely, the CD4bs is the least accessible, with only 10.7% of its surface exposed, making it the most glycan-shielded antigenic region. Notably, the extensive shielding is due to glycans lining the binding site, mostly N276, even though the site itself is not glycosylated. Our findings align with previous reports indicating that the CD4bs is primarily occluded by the glycan shield in the prefusion-closed state, reinforcing the notion that its limited immunogenicity is due to extensive glycan shielding^57^. Albeit V1V2 and V3 loops exhibit high levels of glycan shielding, with only 23.8% and 26.5% accessibility, respectively (Fig. 5f), sites of vulnerability are nevertheless observed at the V2 loop (Fig. 5b, e) and at the base of V3 (Fig. 5e). Additional MD simulations of an Env model incorporating V2 glycans N185e and N185h, absent in our base model (Supplementary Table 9), reveal further decrease in accessibility at both V2 and V3 loops, while the V3 base remains exposed (see Methods, Supplementary Fig. 9). The MPER constitutes another vulnerable region (Fig. 5c, e): although the membrane contributes only ~0.9–5% of the total occluded ectodomain surface (Fig. 5c), it accounts for over 80% of MPER occlusion with a 7.2 Å probe (Fig. 5f). Thus, unlike other regions primarily shielded by glycans, MPER accessibility is restricted largely by its proximity to the lipid bilayer, underscoring structural constraints that shape its immunogenicity.

### Env tilting modulates MPER peptide epitope accessibility

**Fig. 6:**
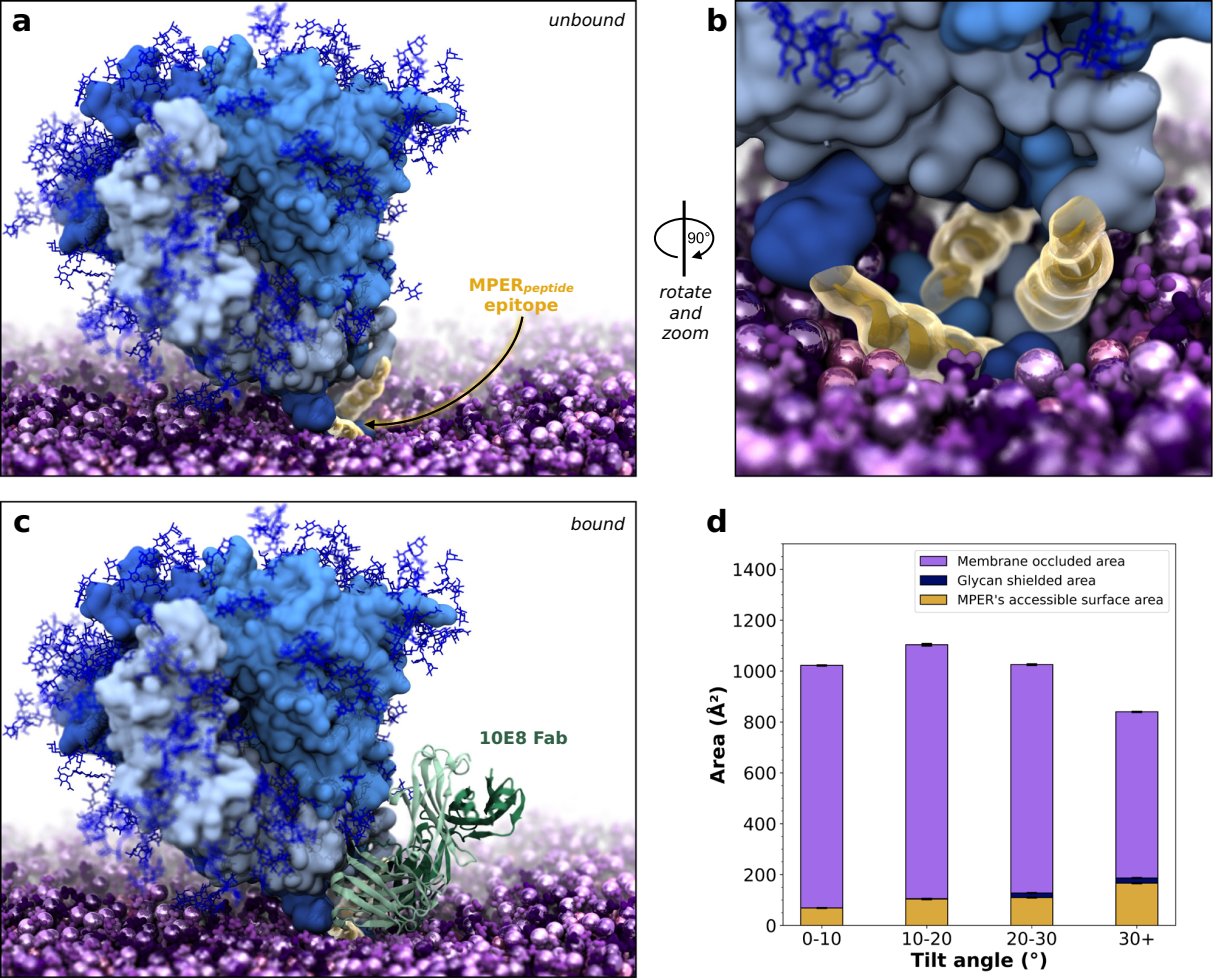
Accessibility of the membrane-proximal external region (MPER) peptide epitope relative to Env tilt angle. **a** Molecular representation of the HIV-1 Env in a tilted conformation shown from a side view, with the MPER peptide epitope (residues 671–683) highlighted in yellow near the viral membrane. N-glycans are depicted as thin, dark blue sticks, while the protein is represented with a light blue surface, with each protomer differentiated by distinct shades. The membrane is depicted in purple, with lipid headgroups represented as van der Waals spheres. **b** Rotated and zoomed-in view of the MPER peptide region (yellow), focusing on its spatial arrangement within the viral membrane (purple), with two MPER peptides positioned closer to the viewpoint and prominently exposed, and the third further away buried in the membrane. **c** The 10E8 Fab (green cartoons) was docked onto the Env by fitting the MPER peptide from the 10E8 Fab–MPER complex (PDB ID: 4G6F^56^) to the most exposed MPER region sampled in our simulations, demonstrating how it fits into the structural pocket formed by tilting-induced rearrangements of the MPER. **d** Quantification of the MPER accessible surface area (yellow), glycan-shielded area (dark blue), and membrane-occluded area (purple) as a function of Env tilt angle (x-axis: 0–10°, 10–20°, 20–30°, >30°). Values were pooled across five independent simulation trajectories initiated with distinct velocity seeds and grouped into four tilt-angle ensembles. For each frame, only the protomer with the maximum MPER accessible surface area was considered. The number of sampled frames contributing to each ensemble was 10,625, 6,085, 23,228, and 12,567, respectively. For each ensemble, mean surface areas were calculated from all sampled frames within that bin. Error bars represent the standard error of the mean (SEM) calculated from the pooled frame-level values in each ensemble. Source data are provided as a Source Data file.

The MPER is a highly conserved domain essential for membrane fusion and a major target for broadly neutralizing antibodies^56,79^. Yet, its proximity to the membrane poses major challenges for effective targeting in vaccine and therapeutic design^56,79–81^. The MPER presents several epitopes distributed along its sequence (residues 660–683), capable of eliciting bNAbs with varying levels of potency. Among these, 2F5 predominantly targets the MPER’s N-terminal α-helix (residues 656–671)^77^, Z13e1 mostly binds the MPER’s hinge point (residues 666–677)^76^, 4E10^75^ and 10E8^56^ primarily recognize the MPER’s C-terminal α-helix (671–683), here referred to as MPER peptide epitope (Fig. 6a-c). One of the broadest and most potent is 10E8^56^, which was found to be particularly sensitive to residues 681–683.

In Fig. 6a, the Env trimer is shown in a tilted state, illustrating how this conformational change affects the spatial positioning of the MPER. As Env tilts, the three MPER peptides become differentially accessible (Fig. 6a, b): the MPER on the tilt-facing side is buried deeper into the membrane and less exposed, whereas those on the opposite side are lifted away from the bilayer and become largely unobstructed. This tilting-induced asymmetry creates a pocket-like niche, potentially enabling MPER-directed antibodies to engage the peptide(s) at a more favorable angle of approach. To test whether this pocket could accommodate antibodies targeting the MPER, we aligned the MPER peptide from the 10E8 Fab–MPER complex (PDB ID: 4G6F^56^) to the corresponding, most exposed MPER region sampled in our simulations (visualization of the alignment is reported in Supplementary Fig. 10). As shown in Fig. 6c, the pocket formed by the tilted Env trimer accommodates the 10E8 Fab, allowing it to bind the MPER peptide epitope without any clashes with the membrane or the rest of the Env glycoprotein. This alignment demonstrates how tilting-induced conformational changes in Env can create structural opportunities for antibody binding, even in regions otherwise heavily occluded by the membrane.

To quantify how Env tilting influences MPER exposure, we clustered MD simulation frames into four tilt ensembles (0–10°, 10–20°, 20–30°, and >30°) and calculated the ASA of MPER epitopes for each (refer to the Methods section for a complete description). A 7.2 Å probe was used to approximate the Fab CDR, and values were filtered to report the most exposed MPER peptide among the three protomers (Fig. 6d). At lower tilt angles (0–10°), the MPER peptide remains predominantly occluded by the viral membrane, with minimal accessibility for potential interactions. As the tilt angle increases, the extent of membrane occlusion decreases significantly, leading to greater MPER exposure at tilt angles above 30° (Fig. 6d). Finally, following the same approach used for the MPER epitope, we probed whether a similar relationship between Env tilting and epitope accessibility was also in place for the gp120-gp41 interface and the CD4bs epitopes (Supplementary Fig. 11). The analysis revealed that, for both epitopes, Env tilting imparts no significant effect on their ASA. In these cases, in contrast to the MPER peptide epitope, the glycan shield remains the dominant contributor to their occlusion. Altogether, these findings emphasize the role of Env plasticity in creating opportunities for antibody binding to otherwise occluded regions, offering insights relevant to immunogen design and small-molecule targeting of the MPER.

## Discussion

In this study, we investigated the conformational dynamics of the HIV-1 Env within its native-like membrane environment using all-atom MD simulations and cryo-ET. Our simulations showed that Env undergoes a pronounced tilting motion relative to the membrane normal. Cryo-ET provides an empirical assessment of Env’s full range of motion, revealing a median tilt angle of 24.3°, in line with the tilt range observed in the simulations. Notably, our findings are consistent with previous structural studies that reported tilting of Env in both its liganded and unliganded states^6,7,39^. Among these, Prasad et al.^6^ provided direct evidence of Env tilting in the unliganded state using cryo-ET, laying the groundwork for understanding this motion. Building on their observations, we now characterize the full range of tilt angles through both simulations and cryo-ET. Richard et al.^39^ investigated Env tilting in its complex with CD4, reporting a median tilt angle of 22.7°, which closely resembles what we report here for Env in the unbound state. Rantalainen et al.^7^ proposed a progressive binding mechanism in which MPER-targeting antibodies, such as 10E8, wedge between the ectodomain and the membrane to induce tilt. While antibody- or receptor-induced tilting remains plausible, our findings clearly show that Env can independently adopt tilted states that expose the MPER for antibody recognition, underscoring tilting as an intrinsic property of the trimer^6,7,39^. Overall, by quantifying Env’s tilting in detail, we reconcile previous observations and provide mechanistic insights into how this motion shapes Env vulnerability.

Although we have taken deliberate and cautious steps to construct a coherent and biologically plausible model of Env, we emphasize that the absence of experimentally resolved coordinates for the full-length structure, particularly across the junction between the ectodomain and the MPER–TMD region, introduces an unavoidable degree of uncertainty. However, our computational results are corroborated by the cryo-ET data presented in this work, lending additional confidence to the validity of our model. Moreover, superimposition with the MPER crystal structure (PDB ID: 4G6F^56^) reveals remarkable structural similarity (Supplementary Fig. 10), further supporting the accuracy of our construct. We note that our simulations do not fully account for the natural curvature and tension of the viral membrane, nor the presence of the underlying matrix (MA) lattice. In this study, Env was embedded in a flat lipid bilayer, which, while closely matching the viral membrane in lipid composition and suitable for studying membrane-proximal interactions, does not fully recapitulate the properties of the HIV-1 viral membrane. In physiological settings, local membrane convexity and MA lattice interactions may accommodate Env tilting motions that extend beyond those observed in our simulations, as captured by cryo-ET data (Fig. 2g). Future extensions involving whole-virion simulations will be needed to incorporate these features and to fully resolve their impact on Env dynamics and tilting.

Our simulations pinpointed two highly conserved N-linked glycans, N88 and N611, which consistently contact the membrane during Env tilting, supporting a role in modulating the trimer’s propensity to tilt. This finding adds to prior evidence highlighting the critical role of N88 and N611 glycans in shaping Env’s antigenicity^72,82,83^. Previous studies demonstrated that glycans at N88 and N611 contribute significantly to the binding of antibodies targeting the gp120-gp41 interface epitope^72,82,83^, with their removal affecting the neutralization potency of such antibodies. Our results further emphasize the multifaceted roles of N88 and N611 glycans, demonstrating their involvement in the modulation of Env’s orientation relative to the membrane, with tilted states showing increased accessibility to MPER-directed antibodies. Although our mutant simulations indicate that Asn to Ala substitution at the N88 and N611 glycosylation sites reduces Env’s tilting propensity, this finding remains computationally derived. Additional functional assays are warranted to corroborate the contribution of N88 and N611 to regulating tilting and the associated immunogenic implications; such validation could be pursued, for example, through neutralization assays with MPER-directed antibodies and biochemical binding assays to quantify antibody binding on intact virions as a proxy for MPER accessibility. Moreover, we note that while our conclusions regarding the role of N88 and N611 in modulating Env tilting are robust within the clade A background and the Env glycoform analyzed here, the presence of additional membrane-proximal gp41 N-linked glycans, such as N618 and N625, may quantitatively affect the magnitude or propensity of Env tilting—a relevant avenue for future investigation. Importantly, tilting itself is an intrinsic property of Env: even in the absence of the N88 and N611 glycans, the structural organization of Env indicates that such transitions are still plausible, albeit potentially less favored on short timescale. In this work, we dissected the precise interactions of N88 and N611 glycans with the lipid bilayer, revealing diverse interaction patterns. Interestingly, these glycans often engaged cooperatively rather than independently. Such cooperative engagement likely stabilizes Env in tilted states by providing additional anchoring points in the lipid bilayer. This dynamic behavior, mediated by hinge regions and glycan–membrane interactions, appears to be a shared feature across class I viral fusion glycoproteins, including SARS-CoV-2 spike^40,41,43,44,46,84^, and influenza hemagglutinin^48,85^. By exploiting conformational flexibility and glycan-mediated stabilization^40,46,86^, viral glycoproteins optimize host engagement, fusion, and immune evasion. These conserved mechanisms are functionally essential and may offer opportunities for therapeutic intervention. N-glycosylation has also been reported to modulate the conformational landscape of non-viral proteins such as GRP94, where site-specific glycosylation alters functional states and ligand-binding preferences^87,88^. Similarly, in IgG1, glycosylation has been shown to influence local structural stability and receptor-binding affinity by modulating the flexibility of key interface regions.^89^ These examples illuminate the broader principle that glycans can act as dynamic regulators of protein structure and function, reinforcing the general relevance of our findings.

Interestingly, tilting appears to be facilitated not only by glycan–membrane interactions involving N88 and N611, but also by the intrinsic plasticity of the MPER and TMD regions, which accommodate the transition through coupled hinging motions (Fig. 4). It is important to note that the hinge angles observed in our simulations diverge from those in the initial model, whose coordinates were mostly derived from the NMR structure (PDB ID: 7LOI^37^). This demonstrates the importance of integrating environmental and structural components into the model. On the other hand, NMR structures or in silico models that exclude the Env ectodomain may be limited in their ability to capture the broad conformational space explored by the MPER and TMD regions. This comparison illustrates how simulations can complement NMR-based approaches by characterizing flexible regions that are otherwise difficult to resolve experimentally. Importantly, this remarkable conformational plasticity has direct implications for antigenicity, as the MPER—unlike other antigenic sites on Env, which are primarily governed by glycan shielding—remains largely occluded due to its proximity to the viral membrane. Our simulations showed that as the ectodomain tilts, the spatial orientation of the MPER peptides changes: those located on the side of the tilt become more deeply buried, while those on the opposite side are lifted away from the membrane, increasing their exposure (Fig. 6). In this tilted conformation, the MPER forms a pocket-like niche that can accommodate the 10E8^56^ Fab without steric clashes (Fig. 6c). Quantitative surface accessibility measurements across tilt-based ensembles further support this mechanism, showing a marked increase in MPER peptide exposure at tilt angles >30°, while the peptide remains largely inaccessible at low tilt angles (0–10°) (Fig. 6d). This sets MPER apart from other epitopes, such as the CD4bs and gp120-gp41 interface, which remained unaffected by tilting and whose accessibility is primarily regulated by glycans. Notably, this finding provides direct mechanistic support for the hypothesis put forward by Prasad et al.^6^, who envisioned that tilting propensity could influence MPER sensitivity to broadly neutralizing antibodies.

Building on this, we also assessed how Env’s glycan shield may contribute to immune evasion by quantifying surface accessibility across different antigenic regions. While prior investigations were limited to water-sized probes (1.4 Å)^90,91^ and performed with different tools such as NACCESS^92^ or GLYCO^91^, here we use an antibody-sized probe designed to reflect accessibility to the CDR of an antibody, following a similar approach to that used by Casalino et al.^40^ for the SARS-CoV-2 spike protein. The dynamic view of the Env’s glycan shield presented here further underscores its remarkable density, particularly in comparison to the SARS-CoV-2 spike protein^40^. While the Env’s glycan shield is broadly effective, our analysis revealed clear epitope-specific differences in the degree of shielding. The FP and the gp120-gp41 interface were the most accessible regions. This result likely reflects the functional importance of these sites in viral entry and is consistent with their known vulnerability to bNAbs. Conversely, the CD4bs was the least accessible epitope. Our findings are consistent with previous reports showing that the CD4bs is primarily occluded by flanking glycans in the prefusion-closed state, reinforcing the notion that its limited immunogenicity is due to extensive glycan masking.^57^ V1V2 and V3 also exhibited high levels of glycan shielding. This is consistent with the neutralization mechanisms of bNAbs targeting these regions, such as PG9^63^ and PGT145^61^ for V1V2, which rely on interactions with glycans N156 and N160, and PGT121^61^ and BG18^65^ for V3, which require contacts with N137 and N332 to engage the glycan supersite epitope. Importantly, glycan N332 is absent in the HIV-1 strain modeled here, suggesting that V3 accessibility may be lower in strains where this glycan is present. In our additional simulations incorporating V2 glycans (N185e and N185h), accessibility of V2 loop is markedly reduced, and, to a lesser extent, that of V3 crown. Nonetheless, a noticeable site of vulnerability persists at the conserved V3 base, indicating that this region, already partially targeted by PGT121^61^ and BG18^65^, may represent an exploitable target even in the prefusion closed state. These findings highlight how even subtle changes in glycan occupancy can markedly reshape the local antigenic landscape and emphasize the need to consider strain-specific glycan composition in immunogen design. On the other hand, the silent face displayed the second-lowest level of accessibility. This is expected, as this region depends on interactions with glycans N262 and N295 for bnAb engagement, as seen with VRC-PG0562^70^. However, given its partial, albeit minimal, exposure, the silent face may represent an underexplored immunogenic region that could become more accessible under specific conformational states or alterations in glycan composition. Within this context, complementary computational approaches, such as energy decomposition frameworks^93,94^ and emerging large language model-based predictors^95^, could be leveraged alongside MD simulations to identify putative epitopes more efficiently.

Taken together, these observations may point to actionable strategies for immunogen design. By stabilizing Env in specific tilted conformations, or mimicking these dynamics in immunogens, it may be possible to increase the exposure of vulnerable regions, such as the MPER, for effective antibody recognition. For example, aptly engineered glycans at position N88 or N611 could be used to tune Env tilting propensity. Such constructs could also reveal druggable pockets, paving the way for small-molecule inhibitors. This strategy may also apply to other class I fusion glycoproteins, such as influenza HA, where tilting-mediated epitope exposure has been reported^48^. Furthermore, the glycan-modulated Env’s tilting dynamics observed here suggests that glycan engineering, through deletion or addition of sites at the membrane-proximal region, could be harnessed to fine-tune trimer stability in its native environment, offering clear potential advantages for structural studies with cryo-ET. In conclusion, these findings emphasize the functional relevance of Env tilting in regulating MPER epitope exposure and underscore the importance of incorporating such dynamics into the rational design of MPER-focused immunogen. Overall, this work exemplifies the power of integrating MD simulations with structural biology techniques to illuminate HIV-1 Env dynamics.

## Methods

### Modeling of the full-length HIV-1 Env glycoprotein

A multi-step modeling protocol, described below, was carried out to build the model of the full-length HIV-1 Env glycoprotein. The ectodomain model (HXB2: residues 31–662) was built based on the crystal structure of BG505 SOSIP.664 trimer solved at 3.5 Å (PDB ID: 5T3Z)^23^. The SOSIP mutations and the furin cleavage site were reversed to match the gene encoding the clade A HIV-1 Env (UniProt accession number: Q2N0S6). The missing gaps of the structure were modeled as disordered loops using Modeller 10.3^96^. A total of 100 independent models of the gp120 and gp41 subunits were generated. The top models, determined based on their scoring metrics, were selected to construct multiple gp160 monomers. Specifically, the gp120-gp41 monomers were reassembled into gp160 monomers and symmetrically combined with each other to generate several models of the ectodomain trimer. These models were further visually inspected, and the model exhibiting the best structural features was selected. Particular attention was given to ensuring that no loops were entangled in knots or clashed with other parts of the structure, ensuring optimal structural integrity.

The MPER, TMD, and CT regions were modeled based on the NMR structure of the entire MPER-TMD-CT region of HIV-1 clade D (PDB ID: 7LOI)^37^. Given that a complete NMR model is not available for clade A, which is the focus of this study, we used i-TASSER to perform homology modeling using the clade D structure as a template and incorporating the clade A sequence^97,98^. Five models of confidence scores of −0.50, −1.04, −1.15, −2.07, and −4.24 were generated from i-TASSER. C-score is typically in the range of [−5,2], where a C-score of higher value signifies a model with high confidence and vice-versa. Model 1, which showed the highest confidence score, was selected. Two cysteines located in the CT of the resulting construct (C764 and C837) were palmitoylated using the lipid-tail functionalization widget available within *Glycan Reader* in CHARMM-GUI^99^.

Since the ectodomain model and the MPER-TMD-CT model were constructed from two independently resolved structures, their direct connection was hindered by misalignment and structural distortions, necessitating additional modeling to achieve an accurate and seamless integration. To address this, we first re-modeled the ectodomain’s HR2 helix up to residue 668 (namely residues 637–668, which encompass part of the MPER’s C-terminal helix, 660–668) using AlphaFold2^100^ and removed the unfolded N-terminal residues (663–668) from the MPER-TMD-CT model. Then, via the re-modeled HR2 helix, the ectodomain was seamlessly connected to the MPER-TMD-CT model, ensuring proper alignment and structural integrity. The connection points were refined through local energy minimization with the autoIMD plugin in VMD^101^. Superimposition of the MPER region in our model with the corresponding MPER region resolved in the crystal structure (PDB ID: 4G6F^56^) revealed remarkable structural similarity, underscoring the high quality of our model (see Supplementary Fig. 10).

### Glycosylation

The HIV-1 Env glycoprotein harbors 23–30 PNGS sites per monomer, which have been shown to be heterogeneously populated across various glycoanalytic studies^9–11^. Therefore, for the scope of this study, a heterogeneous (i.e., not uniform across monomers) site-specific glycoprofile has been derived based on the available glycoanalytic data^9–11^. For the base system, we glycosylated only the PNGS with consistent experimental evidence for occupancy in clade A BG505 viral Env, ensuring biological accuracy and strain-specific relevance^9,10,53,102–105^. This selection resulted in glycosylation at 23 N-linked sites per monomer. A detailed, per-chain description of the site-specific glycoprofile of the HIV-1 Env glycoprotein simulated in this work is provided in Supplementary Tables 1–3. PNGS such as N185e, N185h, N618 and N625 were not glycosylated in our base model. In addition to the base model, we constructed and simulated two Env variants with distinct glycoprofiles: (i) a mutant system in which N88 and N611 were substituted with alanine to delete the respective N-linked glycans, and (ii) a more highly glycosylated construct that includes the N185e and N185h glycans located on the V2 loop (see Supplementary Table 9). Glycans were modeled using the *Glycan Reader & Modeler* tool^106^ available in CHARMM-GUI^99^. We note that the oligosaccharides (GlcNAc-/GlcNAc2-/ManGlcNAc2-) originally solved in the cryo-EM structures have generally been preserved and used as a scaffold wherever feasible for constructing complete glycans. However, moderate adjustments to glycan dihedrals and/or asparagine side chains have been carried out at densely glycosylated sites to eliminate steric clashes and accommodate all the glycans.

### Membrane building

The lipid composition of the membrane patch was selected based on the experimental studies of the HIV-1 plasma membrane compositions^107–110^. The outer and inner leaflet abundances were derived from previous MD simulations and lipidomic data of mammalian cell plasma membranes^111^ (Supplementary Table 4). An asymmetric lipid membrane patch (200 Å × 200 Å) was constructed using CHARMM-GUI input generator^99,112^. The ratio of short-chain/saturated to long-chain/unsaturated phosphatidylcholine (PC) lipids was set to 40:60^108^. These fractions were adjusted to compensate for the simplification of the membrane, which artificially increased the lipid count of the inner leaflet due to bilayer asymmetry.

### System preparation

After functionalization (i.e., glycosylation and palmitoylation) of the HIV-1 Env glycoprotein, protonation states were assigned using PROPKA3^113^ at pH 7.4. The generated models were parameterized using *psfgen* and the CHARMM36m all-atom additive force fields for proteins, lipids, and glycans^114–116^. The systems were solvated with TIP3P^117^ explicit water model in a box, ensuring a minimum distance of 24 Å between the protein and the box edges. Na^+^ and Cl^-^ ions were added at a concentration of 150 mM to neutralize the charge of the system. Solvation and charge neutralization were performed using the *solvate* and *autoionize* plugins within VMD^101^.

### Modeling of the N88A + N611A mutant system

The Env mutant model was created by introducing alanine substitutions at positions N88 and N611, effectively deleting the respective N-linked glycans. These alanine mutations were chosen to eliminate the glycosylation sites without introducing significant structural perturbations to the surrounding regions. Starting from the glycosylated base model embedded in the lipid bilayer, we first removed the N-glycans linked to N88 and N611 and then we introduced the N88A and N611A mutations at the same time across the three protomers using *psfgen* within VMD^101^. Following the same procedure used for the base model, the resulting Env mutant model was embedded into an asymmetric lipid bilayer mimicking the composition of the viral budding site, solvated and charge-neutralized with Na⁺ and Cl^−^ ions at a concentration of 150 mM. The resulting system was then equilibrated and subjected to MD simulations using the same protocol described below for the base model.

### Modeling of the N185e/h occupied Env system

To assess the impact of glycan occupancy at positions N185e and N185h on the overall architecture of the Env glycan shield, we generated a full-length Env model in which these two PNGS were occupied. Glycan compositions for these sites were assigned according to the experimentally derived profiles^105^ (see Supplementary Table 9). Starting from the glycosylated base model, the two glycans were introduced across all three protomers using the *Glycan Reader & Modeler* tool^106^ available in CHARMM-GUI^99^. Given the close spatial proximity of N185e and N185h, the system was subjected to local energy minimization in VMD^101^ to relieve steric clashes and optimize glycan–protein interactions prior to further processing. Following the same procedure used for the base model, the glycosylated Env model was embedded into an asymmetric lipid bilayer mimicking the composition of the viral budding site, solvated, and charge-neutralized with Na⁺ and Cl^−^ ions at a concentration of 150 mM. The resulting system was then equilibrated and subjected to MD simulations using the same protocol described below for the base model.

### Molecular dynamics (MD) simulations

All-atom MD simulations were performed on the Triton Shared Computing Cluster (TSCC) at the San Diego Supercomputer Center (SDSC) using NAMD3^118^. A multi-step approach involving repeated minimization cycles, NVT melting (for the membrane), and NPT equilibration was applied to relax the systems. Initially, the systems were subjected to 10,000 energy minimization steps using the conjugate gradient energy method during which the protein, glycans, lipid heads (P atom for DPPC, POPC, POPE, PSM, and POPS, and O3 atom for CHOL), solvent, and ions were kept fixed. Next, to allow equilibration of the lipid tails (i.e., melting), the temperature was gradually increased from 10 K to 310 K over 0.5 ns (NVT ensemble) using a time step of 1 fs. This was followed by a further 20,000 steps of energy minimization, during which positional harmonic restraints with a force constant of 1.0 (kcal/mol)/Å^2^ were applied to the protein and glycan atoms. A 0.5 ns NPT (isothermal-isobaric) equilibration was then conducted at 2 fs/step using an anisotropic pressure coupling scheme (*useFlexibleCell yes, useConstantArea no*) with the harmonic restraints on protein and glycan retained. Next, all restraints were released, and another 0.5 ns NPT (isothermal-isobaric) equilibration was performed. Lastly, the pressure coupling was switched to a semi-isotropic scheme (*useFlexibleCell yes, useConstantArea yes*), and another 20 ns of NPT equilibration was conducted. Prior to production MD simulations, all the systems were meticulously inspected to ensure no structural artifacts were present. Five replicates of NPT production MD simulations with semi-isotropic pressure coupling were performed for 1.05 µs. It is important to note that all replicates were conducted independently, from their respective minimization and continuing through to the production run. All simulations were performed with an integration time step of 2 fs, utilizing the SHAKE^119^ algorithm to constrain covalent bonds involving all hydrogen atoms. Periodic boundary conditions were employed during the simulations, with the particle-mesh Ewald^120^ method used for long-range electrostatics calculations. A maximum grid spacing of 2 Å was applied. Non-bonded interactions, including van der Waals forces and short-range electrostatics, were handled using a cutoff distance of 12 Å. Control of the temperature and pressure during the simulations was achieved with a Langevin thermostat^121^ (310 K) and a Nosé-Hoover Langevin barostat^122,123^ (1.01325 bar) as implemented in NAMD3. A summary of the simulations performed in this work is provided in Supplementary Table 5.

### RMSD analysis

To assess proper system equilibration, backbone root-mean-square deviation (RMSD) analyses were performed over the first 50 ns of each simulation using MDAnalysis^124^. For each system, five independent replicas were analyzed separately. Prior to analysis, trajectories were aligned to the initial frame using alpha carbon (Cα) atoms as the alignment mask, and solvent and ions were removed. RMSD was calculated over protein backbone atoms using the initial structure of each replica as the reference. Time-series RMSD values were computed throughout the first 50 ns of each simulation. As shown in Supplementary Fig. 12, all replicas exhibited stable RMSD behavior over the first 50 ns of simulation, indicating proper equilibration of simulated systems.

### Tilt angle calculation

The tilt angle of the Env protein relative to the membrane normal was calculated using an in-house *Tcl* script executed within VMD^101^. The tilt angle was defined by three points: (i) the center of mass (COM) of the HR1C protein core (residues 540–565, HXB2: 572–597), (ii) the center of mass (COM) of the Cα atoms of residue 651 (HXB2: 683) across the three protomers (vertex), and (iii) a point along the membrane normal originating from this position. At each simulation frame, vectors connecting the vertex to the core COM and along the membrane normal were constructed, and the tilt angle was calculated as the angle between these vectors using the dot product. Based on the tilt angle, each frame was assigned to a conformational ensemble corresponding to predefined angle ranges (e.g., either 0–10°, 10–20°, 20–30°, or >30°). At the end of this procedure, the four ensembles representing the four tilt angle ranges were generated and used for subsequent collective analysis.

### Glycans-membrane contacts

Glycans-membrane interactions were analyzed using the *measure contacts* command in VMD^101^, supplemented by custom-made *Tcl* scripts. Contacts were defined using a 4.5 Å cutoff distance between glycan and membrane heavy atoms on a per-residue basis. For each glycan, frames from all replicates (R1–R5) were pooled, and the contact frequency was calculated as the percentage of frames exhibiting interactions relative to the total number of frames. For the N88 and N611 glycans, the time evolution of their contacts with the membrane was analyzed, along with an assessment of the cooperativity of their interactions. This analysis evaluated the simultaneous interactions of multiple glycans, either originating from the same site (e.g., two N88 or two N611) or from different sites (e.g., one N88 and one N611).

Next, we sought to dissect the nature of such contacts formed between the Env glycans at N88 and N611 and the lipid molecules using PyContact^125^. PyContact employs a sigmoidal, distance-dependent scoring function that assigns values close to 1 for close contacts and smoothly decays to 0 at the cutoff distance. A cutoff of 4.5 Å was used to define glycan–lipid interactions. For each replicate trajectory (R1–R5), contact scores were first accumulated on a per-residue basis across all frames. These residue-level scores were then pooled together by molecular species across all replicates, grouping either into glycan residues (e.g., Sia, GlcNAc, Man, Fuc, Gal) or lipid residues (e.g., POPC, POPE, DPPC, PSM, CHOL, POPS). Pooled scores were expressed as percent contributions relative to the total contact score, thereby highlighting the relative engagement of each species. For lipids, an additional normalization step was performed to account for differences in leaflet composition: pooled scores were divided by the number of lipid molecules present in the outer leaflet (POPC = 150, POPE = 108, DPPC = 120, PSM = 248, CHOL = 416, POPS = 0). This allowed us to compare lipid-level contributions on a per-species basis, independent of both absolute score magnitude and molecular abundance.

### Conservation analysis of Env

To analyze the evolutionary conservation of gp120 and gp41, we used the Evolutionary Conservation Profiles of Proteins server (Consurf)^126–129^. Homologues were retrieved from the UniProt Reference Clusters database at 90% sequence identity (UNIREF90) using the HMMER algorithm with an E-value cutoff of 0.0001 and a single iteration. The homologous sequences were refined by clustering at 95% sequence identity using the Cluster Database at High Identity with Tolerance algorithm (CD-HIT), retaining the highest-scoring sequence when overlaps exceeded 10%. Homologues with less than 60% coverage of the query sequence were excluded, and up to 300 sequences were sampled for further analysis. The multiple sequence alignments were generated using the Multiple Alignment using Fast Fourier Transform method (MAFFT). A phylogenetic tree was constructed using the Neighbor Joining method (NJ) with maximum likelihood distances. Conservation scores were calculated using a Bayesian approach with the most appropriate amino acid substitution model determined by best fit.

### MPER and TMD hinge angles calculation

The MPER and TMD hinge angles were calculated using a custom *Tcl* script executed within VMD^101^. For each protomer, the MPER hinge angle was defined by three coplanar points: the alpha carbon (Cα) of residue 624, the COM of the Cα atoms of residues 635–639, and the Cα of residue 651. Similarly, for the TMD hinge angle, three coplanar points were defined: the Cα of residue 630, the Cα of residue 651, and the Cα of residue 664. At each simulation frame, vectors were constructed from the central point, and the hinge angles were calculated as the angles between these vectors using the dot product.

### TMD inclination angles calculation

The TMD inclination angle was calculated using a custom *Tcl* script executed within VMD^101^. For each protomer, the angle was defined by three points: (i) the Cα atom of residue 664, (ii) the Cα atom of residue 652 (vertex), and (iii) the projection of residue 664 onto the membrane plane at the z-coordinate of residue 652. At each simulation frame, the inclination angle was computed as the angle between the TMD axis and its projection onto the membrane plane using the dot product.

### Accessible surface area (ASA)

ASA was calculated using the *measure sasa* command in VMD^101^, which utilizes the Shrake and Rupley algorithm^130^, in combination with custom *Tcl* scripts. The ASA was computed using probe radii ranging from 1.4 Å to 8 Å. The glycan-shielded area was determined by subtracting the ASA of the *protein-with-glycans* from the ASA of the *protein-without-glycans*. Similarly, the membrane-occluded area was calculated by subtracting the ASA of the *protein-with-glycans-and-membrane* from the ASA of the *protein-with-glycans*. ASA values were computed at 1 ns intervals throughout the simulations and averaged across all replicas, and the associated variability was quantified by the standard deviation.

### Epitope-specific ASA

The ASA of key antigenic regions of the Env trimer including the MPER (residues 628–651) the gp120-gp41 interface (residues 4–19, 46–56, 59–64, 207–209, 503–511, 580–584, 586–593, 596–606), the CD4bs (residues 333–341, 397–400, 423–430, 435–443), the V1V2 loop (residues 94–168), the V3 loop (residues 266–299), the fusion peptide (FP) (residues 480–494), and the silent face epitope (residues 303–306, 413–416, 260–263, 222, 183–184, 27–29) was computed. We note that the residue numbering provided here corresponds to our Env model, which may differ from the standard HXB2 reference sequence. For accurate mapping to HXB2 refer to Supplementary Fig. 1. ASA values were computed at 1 ns intervals across all simulations following the same methodology described in the previous section. The analysis was performed using a 7.2 Å probe radius approximating the size of the CDR of an antibody’s Fv. The values were averaged across all replicas and the associated variability was quantified by the standard deviation.

### MPER peptide ASA

To evaluate the accessibility of the MPER peptide (residues 671–683), the trajectory frames were clustered into four distinct ensembles based on the degree of Env tilting observed (i.e., 0–10°, 10–20°, 20–30°, and > 30°). These ensembles were then used to calculate the ASA as a function of the Env’s tilt angle, providing insights into how tilting affects the epitope accessibility. For every frame within each ensemble, ASA values were computed separately for each Env’s protomer using a 7.2 Å probe radius, approximating the size of the CDR of an antibody’s Fv. The maximum ASA value was then selected to identify the most accessible protomer. The selected ASA values were averaged within each ensemble. Uncertainty in the mean was estimated as the standard error of the mean, computed from the frame-level distributions. Corresponding glycan-shielded and membrane-occluded areas were calculated for each tilt angle range as described in the previous section.

### Cloning of enveloped virus-like particles (eVLP)

The full-length envelope glycoprotein sequence for BG505 was obtained from UniProt (ABA61515.1) and reverse transcribed using GenScript’s codon optimization tool and optimized for human expression. GenScript cloned this fragment into the pcDNA3.1(+) backbone and upon receipt, this plasmid was expanded by transformation in Stbl3 competent *E. coli* cells. To clone in the signal peptide of gp160, the second BstEII restriction site was mutated, and the peptide sequence was added by Gibson assembly. To improve Env expression in the eVLPs, an endocytosis prevention motif (EPM) and an ESCRT and ALIX-binding region (EABR) sequence were added to the C-terminal side of the Env sequence. This technology is described in Hoffmann et al.^54^.

### eVLP expression and purification

293FT cells (Thermo Fisher, cat. no: R70007) were grown to 80-90% confluency in either T75 or 15 cm dishes, and fresh media were added just prior to transfection of the eVLP plasmid using FuGENE transfection reagent. The transfected cells were incubated at 37 °C and 5% CO_2_ for 72 h. After 72 h, the supernatant was collected and centrifuged at 400 × g for 10 min. The supernatant was filtered through a 0.45 µm syringe to remove excess cell debris that remained after centrifugation and then layered on top of a 5 mL 20% sucrose cushion in an ultracentrifuge tube. Earlier sample preparations were concentrated on a 100 kDa MWCO spin column prior to layering on a sucrose cushion, but this step was part of a less optimal workflow and thus removed in future iterations. The sample was spun at 135,000 × g for 2 h at 4 °C. The supernatant was carefully removed and the pellet re-suspended in 1xPBS. The re-suspended eVLPs were incubated in the ultracentrifuge tube overnight at 4 °C. The following morning, the eVLPs were carefully mixed and transferred to a microtube and treated with DNase for 15 min at room temperature just prior to plunge freezing on a cryo-EM grid. In earlier sample preparations, the sample was rinsed 3 times with 1xPBS after overnight incubation to reduce viscosity. The wash steps were completed by applying the sample to a 100 kDa spin column and spinning it at 10,000 × g at 4 °C. After the final rinse, the column was spun to reduce the final volume of the sample to 20–50 µL and then plunge frozen on a cryo-EM grid.

### Cryo-EM grid preparation of eVLPs

Before sample deposition, copper QUANTIFOIL R 2/1 cryo-EM grids were plasma-cleaned for 60 s using a Pelco plasma cleaner equipped with air at 25 W power. 4 µL of purified eVLPs was applied to the grids and back-blotted using the Leica EM GP2 Automatic Plunge Freezer and then plunge frozen into liquid ethane or a 50/50 ethane propane mix. The blot time varied from 4 to 10 s. Multiple blot times would be used for each eVLP sample to determine the best conditions for that sample preparation.

### Cryo-ET data collection

Initial datasets were collected on a Titan Krios-3 TEM (Thermo Fisher) operating at 300 kV with K3 direct electron detector (Gatan) at a nominal magnification of 64,000x (pixel size 1.341 Å, counting mode). A 70 mm objective aperture was inserted. A Gatan zero-loss imaging filter with a 15 eV-wide slit was used throughout the data collection. The defocus range was −3 to −5 μm. PACE-tomo in serialEM^131^ was used for collecting tilt series automatically. Targets were selected manually to maximize the number of eVLPs that could be collected within each tilt series. For each tilt series, a dose-symmetric scheme was applied with a tilt range of −54° to +54° with an increment of 3°, resulting in 37 tilt images being collected with 8 frames in each tilt. The total dose was 140 e^−^/Å^2^ and the dose rate was set at 15.9 e^−^/Å^2^/s. The protocol describing detailed steps in data collection is available online^132^. The later datasets that were used for subtomogram averaging were collected on a Titan Krios G4 TEM (Thermo Fisher) operating at 300 kV with a Falcon 4i direct electron detector (Thermo Fisher) at a nominal magnification of 81,000x (pixel size 1.561 Å, counting mode). A Selectris X energy filter with a 10 eV-wide slit was used throughout data collection. PACE-tomo was used to collect tilt series following the same conditions described above, with the exception of the dose rate. Falcon4 records electron events at the detector’s physical frame rate (248 Hz), so 224 EER frames were collected per tilt series at a dose rate of 4.4 e^−^/Å^2^/s.

### Tilt series alignment and tomogram reconstruction

Warp^133^ (Linux or Windows) was used for the pre-processing of tomograms before subtomogram analysis. Specifically, motion correction, CTF estimation and gain correction were performed in Warp and then all tilt series were aligned with either AreTomo, or eTomo in IMOD^134,135^ v4.11.25 using patch tracking. The mean residual error (in nm) calculated from eTomo was used to assess the alignment quality for different tilt series. Tomograms with a mean residual error <2 nm were selected for further analysis. Next, the alignment data were fed back to Warp for tomogram reconstruction using weighted back-projection at 10 Å/pxl for data collected on the Krios-3, or 12.488 Å/pxl for data collected on the Krios G4. Tomogram visualization and Env particle picking were performed on deconvoluted tomograms generated from Warp with default settings.

### Env particle picking and initial orientation assignment in Dynamo

IMOD was used to visualize all reconstructed tomograms and manually pick Env subtomograms from VLPs. In total, 261 tomograms were inspected and 6,389 particles were picked. Picking for each VLP was conducted by first placing a point in the center of the VLP using the XYZ view in IMOD as a guide to determine the center in 3-dimensional space, placing a second point on the membrane of the VLP, and then finally points were added for each Env that could be visually identified. The coordinates of selected Envs from each VLP were analyzed in Dynamo in matlab^136–139^ v11509. VLPs are modeled as spheres with manually defined center and radius in Dynamo. The initial orientation of each Env subtomogram are assigned perpendicular to the surface of the VLP model it belongs to. The azimuth angle was randomized to minimize the effect of the missing wedge. The coordinates were then used for further subtomogram analysis in Relion3^140^.

### Subtomogram analysis and three-dimensional model reconstruction

For the 3D reconstruction of Env, a total of 6389 subtomograms were combined and provided to Relion3 following particle extraction with Warp. The subtomograms were binned to 12.488 Å/pxl at this stage. Subtomograms were refined in Relion3 with C1 symmetry first to align the membrane, then followed by a 6-class 3D classification with angular limit to local ranges. To avoid over-fitting, the regularization parameter T was set to 1 instead of default 4 and the resolution E-step was set to 25 Å to disable alignment of high-frequency signals. Good class-averages were chosen for further C1 alignment, and then subsequently unbinned to 6.244 Å/pxl. The unbinned particles were initially aligned using a membrane mask, C1 symmetry, and limits on local angular searches. Further refinement was done using a full Env mask to better align Env, and local searches were limited to in-plane such that Env can rotate on its central axis. Focused angular refinement on either Env or the membrane was performed on this alignment to obtain the values for calculating tilt angles as described below. The final map reported in this study had a resolution of 20 Å as determined by the gold-standard Fourier shell correlation (FSC) using the 0.143 criterion. UCSF ChimeraX^141^ v1.9 was used for all the volume segmentation, figure and movie generation, and automatic rigid-body docking processes.

### Geometric analysis of Env tilt angle relative to VLP surface

After obtaining the Env reconstruction, subtomograms included in the final map were used to calculate their orientation relative to VLP. Envs were assessed for tilting on a per-particle basis. The angle between the Env orientation vector (determined through subtomogram averaging) and a vector perpendicular to the membrane at each respective Env position was determined as the Env tilt angle. To calculate the vector perpendicular to the membrane, focused angular refinement on the membrane region was performed, resulting in a disordered Env ectodomain and a well-defined membrane density. Tilt angle distributions were plotted as a histogram, with 0° indicating no tilt and 90° indicating an orientation parallel to the VLP membrane. A total of six outlier points were removed using the ROUT method with Q = 1%. The outliers values were either close to or greater than 90°, indicating that they were likely misaligned particles.

### Reporting summary

Further information on research design is available in the Nature Portfolio Reporting Summary linked to this article.

## Supplementary information

Supplementary Information

Description of Additional Supplementary File

Supplementary Data 1

Supplementary Movie 1

Supplementary Movie 2

Reporting Summary

Transparent Peer Review file

## Source data

Source Data

## Data Availability

Full MD simulation datasets of the three HIV-1 Env models generated in this study (including production trajectories, initial, equilibrated, and final model coordinates) are available for download on the Amaro Lab database [https://amarolab.ucsd.edu/data.php]. The initial, equilibrated, and final coordinates for all three HIV-1 Env models as well as MD input files are also provided with this article as Supplementary Data 1. Cryo-ET maps have been deposited in the Electron Microscopy Data Bank (EMDB) under accession code EMD-75641. Raw EER tilt-series data and associated metadata files are uploaded to the Electron Microscopy Public Image Archive (EMPIAR) under accession code EMPIAR-13331. The atomic coordinates of the BG505 SOSIP.664 HIV-1 Env trimer crystal structure referenced in this work are available in the Protein Data Bank (PDB) under accession code 5T3Z. The atomic coordinates of the 10E8 Fab in complex with an HIV-1 gp41 peptide crystal structure referenced in this work are available in the Protein Data Bank (PDB) under accession code 4G6F. The atomic coordinates of the HIV-1 gp41 membrane-proximal external region, transmembrane domain, and cytoplasmic tail NMR model referenced in this work are available in the Protein Data Bank (PDB) under accession code 7LOI. Source data are provided with this paper.

## Code availability

This study utilized the standard build of the simulation software NAMD3 (https://www.ks.uiuc.edu/Research/namd/) according to best practices with no special modifications.
