# Supplementary material for: N-Glycans modulate tilting of HIV-1 envelope glycoprotein
Source: Nat Commun. 2026 Apr 15;17:5206. doi: 10.1038/s41467-026-71874-2 (PMC13254402; doi:10.1038/s41467-026-71874-2)
Supplement: Supplementary file 1 — Supplementary Information [file 41467_2026_71874_MOESM1_ESM.pdf]

Supplementary Information

N-Glycans Modulate Tilting of HIV-1 Envelope Glycoprotein

Mohamed Shehata[1§], Lorenzo Casalino[1§], Madeleine Duquette[1§], Siyu Chen[1,2], Alex Flaherty[1], Patrick M. Waller[1], Elizabeth Villa[1,2], Rommie E. Amaro[1]*

[1] Department of Molecular Biology, University of California San Diego, La Jolla, CA, USA.

[2] Howard Hughes Medical Institute, University of California, San Diego, La Jolla, CA, USA.

[§]These authors contributed equally: Mohamed Shehata, Lorenzo Casalino, Madeleine Duquette

*Corresponding author (ramaro@ucsd.edu)

Table of Contents

1. **Supplementary Figures 1 – 12**

2. **Supplementary Tables 1 – 9**

1. Supplementary Figures

```
HXB2                                          MRVKEKYQHLWRWGWRWGTMLLGMLMICSATEKLWVTVYYGVPVWKEATTTLFCASDAKA      60
Full_legnth_env_based_on_uniprot_Q2N0S6       ------------------------------AENLWVTVYYGVPVWKDAETTLFCASDAKA      30
                                                                            :*:*************:* ***********

HXB2                                          YDTEVHNVWATHACVPTDPNPQEVVLVNVTENFNMWKNDMVEQMHEDIISLWDQSLKPCV      120
Full_legnth_env_based_on_uniprot_Q2N0S6       YETEKHNVWATHACVPTDPNPQEIHLENVTEEFNMWKNNMVEQMHTDIISLWDQSLKPCV      90
                                              *:** ******************: * ****:******:****** **************

HXB2                                          KLTPLCVSLKCTDLKNDTNTNSSSGRMIMEKGEIKNCSFNISTSIRGKVQKEYAFFYKLD      180
Full_legnth_env_based_on_uniprot_Q2N0S6       KLTPLCVTLQCTNVTNNITDD--------MRGELKNCSFNMTTELRDKKQKVYSLFYRLD      142
                                              *******:*:**::.*: . :         :**:******::*.:*.* ** *::**:**

HXB2                                          IIPIDN--------DTTSYKLTSCNTSVITQACPKVSFEPIPIHYCAPAGFAILKCNNKT      232
Full_legnth_env_based_on_uniprot_Q2N0S6       VVQINENQGNRSNNSNKEYRLINCNTSAITQACPKVSFEPIPIHYCAPAGFAILKCKDKK      202
                                              :: *::        ....*:* .****.****************************::*.

HXB2                                          FNGTGPCTNVSTVQCTHGIRPVVSTQLLLNGSLAEEEVVIRSVNFTDNAKTIIVQLNTSV      292
Full_legnth_env_based_on_uniprot_Q2N0S6       FNGTGPCPSVSTVQCTHGIKPVVSTQLLLNGSLAEEEVMIRSENITNNAKNILVQFNTPV      262
                                              ******* .**********:******************:*** *:*:***.*:**:** *

HXB2                                          EINCTRPNNNTRKRIRIQRGPGRAFVTIG-KIGNMRQAHCNISRAKWNNTLKQIASKLRE      351
Full_legnth_env_based_on_uniprot_Q2N0S6       QINCTRPNNNTRKSIRI--GPGQAFYATGDIIGDIRQAHCTVSKATWNETLGKVVKQLRK      320
                                              :************ ***  ***:** : *  **::*****.:*:*.**:** ::..:**:

HXB2                                          QFGNNKTIIFKQSSGGDPEIVTHSFNCGGEFFYCNSTQLFNSTWFNSTWSTEGSNNTEGS      411
Full_legnth_env_based_on_uniprot_Q2N0S6       HFGNNTIIRFANSSGGDLEVTTHSFNCGGEFFYCNTSGLFNSTWISNT-SVQGSNSTGSN      379
                                              :****. * * :***** *:.**************:: ******:..* *.:***.* ..

HXB2                                          DTITLPCRIKQIINMWQKVGKAMYAPPISGQIRCSSNITGLLLTRDGGNSNNESEIFRPG      471
Full_legnth_env_based_on_uniprot_Q2N0S6       DSITLPCRIKQIINMWQRIGQAMYAPPIQGVIRCVSNITGLILTRDGGSTNSTTETFRPG      439
                                              *:***************::*:*******.* *** ******:******.:*. :* ****

HXB2                                          GGDMRDNWRSELYKYKVVKIEPLGVAPTKAKRRVVQREKRAVGIGALFLGFLGAAGSTMG      531
Full_legnth_env_based_on_uniprot_Q2N0S6       GGDMRDNWRSELYKYKVVKIEPLGVAPTRAKRRVVGREKRAVGIGAVFLGFLGAAGSTMG      499
                                              ****************************:****** **********:*************

HXB2                                          AASMTLTVQARQLLSGIVQQQNNLLRAIEAQQHLLQLTVWGIKQLQARILAVERYLKDQQ      591
Full_legnth_env_based_on_uniprot_Q2N0S6       AASMTLTVQARNLLSGIVQQQSNLLRAIEAQQHLLKLTVWGIKQLQARVLAVERYLRDQQ      559
                                              ***********:*********.*************:************:*******:***

HXB2                                          LLGIWGCSGKLICTTAVPWNASWSNKSLEQIWNHTTWMEWDREINNYTSLIHSLIEESQN      651
Full_legnth_env_based_on_uniprot_Q2N0S6       LLGIWGCSGKLICTTNVPWNSSWSNRNLSEIWDNMTWLQWDKEISNYTQIIYGLLEESQN      619
                                              *************** ****:****:.*.:**:: **::**:**.***.:*:.*:*****

HXB2                                          QQEKNEQELLELDKWASLWNWFNITNWLWYIKLFIMIVGGLVGLRIVFAVLSIVNRVRQG      711
Full_legnth_env_based_on_uniprot_Q2N0S6       QQEKNEQDLLALDKWASLWNWFDISNWLWYIKIFIMIVGGLIGLRIVFAVLSVIHRVRQG      679
                                              *******:** ***********:*:*******:********:**********:::*****

HXB2                                          YSPLSFQTHLPTPRGPDRPEGIEEEGGERDRDRSIRLVNGSLALIWDDLRSLCLFSYHRL      771
Full_legnth_env_based_on_uniprot_Q2N0S6       YSPLSFQTHTPNPRGLDRPERIEEEDGEQDRGRSTRLVSGFLALAWDDLRSLCLFCYHRL      739
                                              ********* *.*** **** ****.**:**.** ***.* *** **********.****

HXB2                                          RDLLLIVTRIVELLGR-------RGWEALKYWWNLLQYWSQELKNSAVSLLNATAIAVAE      824
Full_legnth_env_based_on_uniprot_Q2N0S6       RDFILIAARIVELLGHSSLKGLRLGWEGLKYLWNLLAYWGRELKISAINLFDTIAIAVAG      799
                                              **::**.:*******:        ***.*** **** **.:*** **:.*::: *****

HXB2                                          GTDRVIEVVQGACRAIRHIPRRIRQGLERILL   856
Full_legnth_env_based_on_uniprot_Q2N0S6       WTDRVIEIGQRLCRAFLHIPRRIRQGFERALL   831
                                               ******: *  ***: *********:** **
```

Supplementary Figure 1. Sequence alignment of the full-length Env model used in this study with the reference HXB2 Env sequence.

FIGURE S2

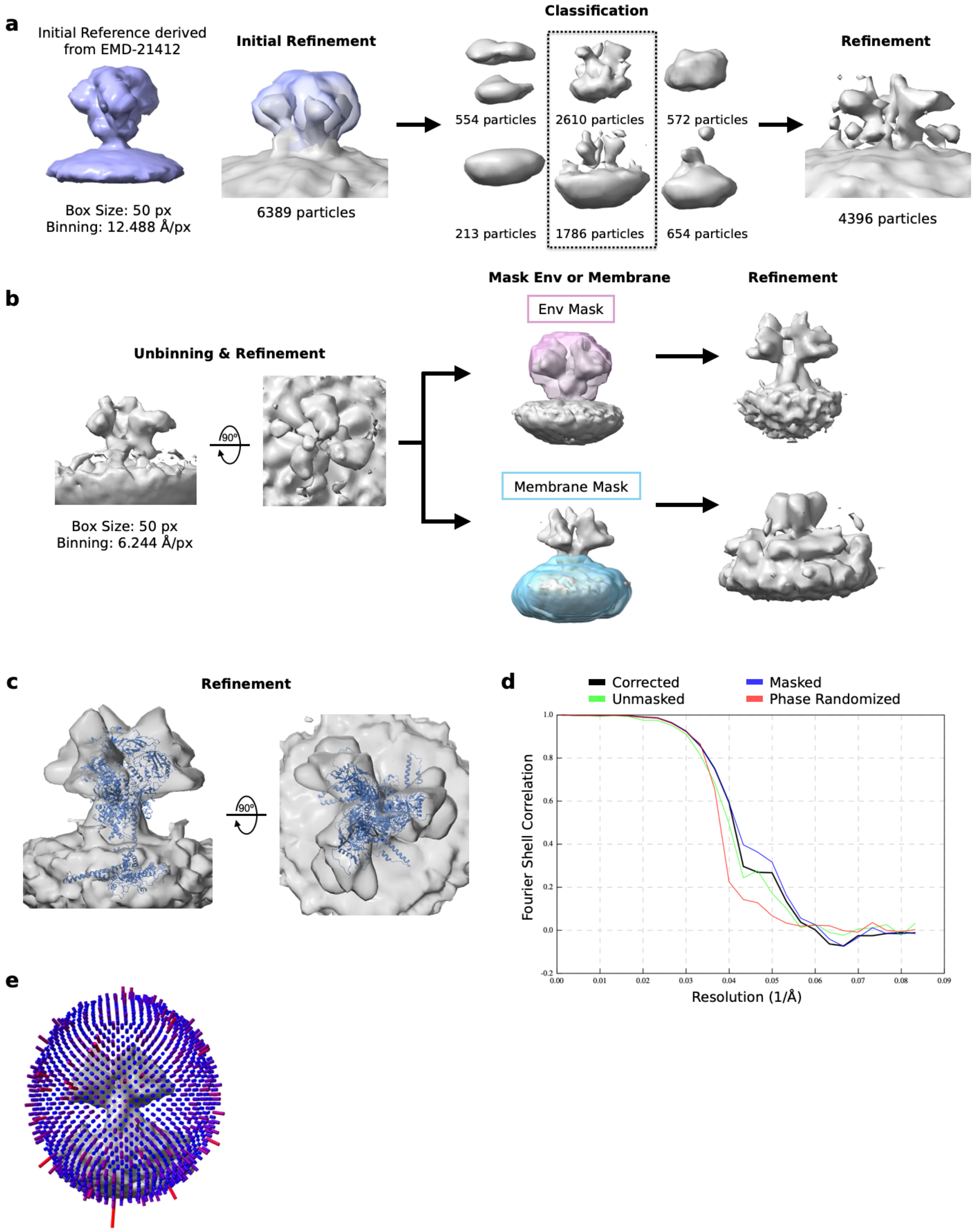

Supplementary Figure 2. (**a**) The initial reference used for refinement in Relion3 was derived from EMD-21412 to roughly align the Env particles. After initial refinement, the particles were classified into six classes to remove junk particles and the most reasonable class was selected for further refinement, as indicated by the box. (**b**) The selected class was unbinned to 6.244 Å/px, further refined, and then mask refined with an Env mask and a membrane mask to generate two separate refinements with an optimally aligned Env or membrane that is used for downstream angle determination. (**c**) Final reconstruction of WT Env with the model from this paper fitted into the density. (**d**) Gold-standard Fourier shell correlation (FSC) curve using the 0.143 criterion. Estimated resolution of 20 Å. (**e**) Angular distribution of the particles used in the reconstruction. C3 symmetry was used in the final reconstruction.

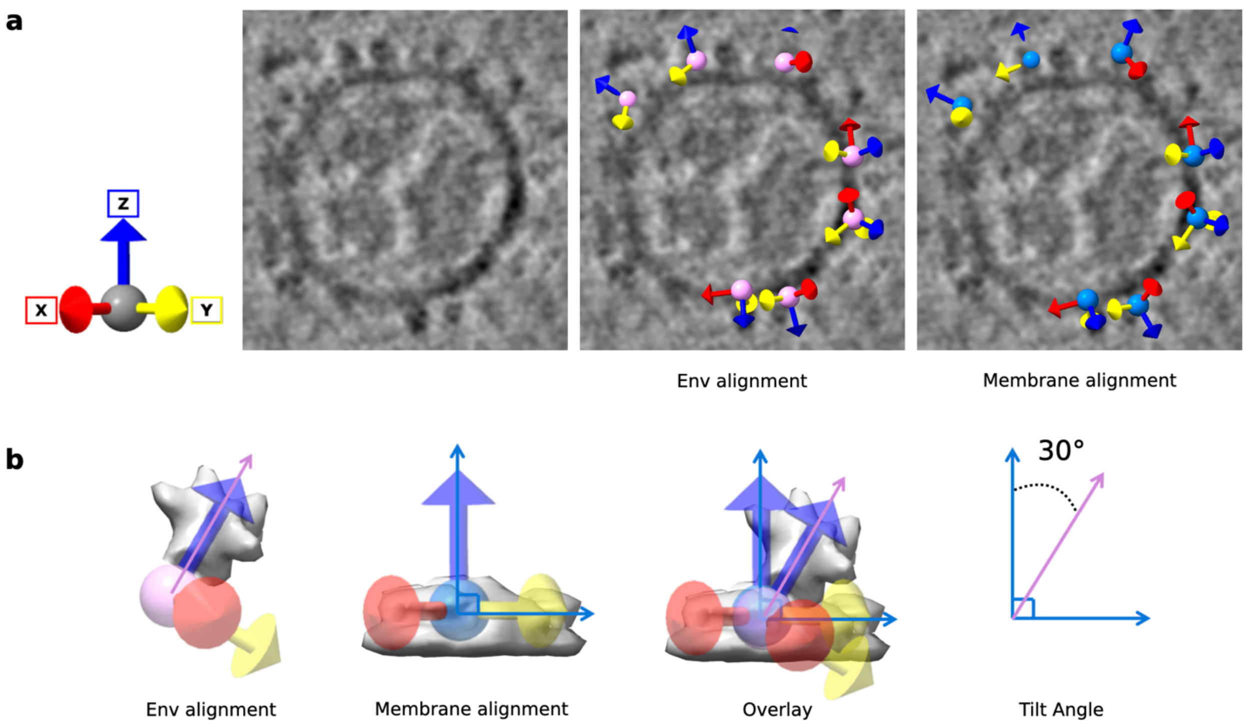

Supplementary Figure 3. (**a**) Particle orientation is depicted with the blue arrow indicating the z-axis, red arrow indicating the x-axis, and the yellow arrow indicating the y-axis. Left: Example tomogram slice shown. Middle: Env-masked and aligned particles, indicated by the pink sphere, overlayed on tomogram slice. Right: Membrane-masked and aligned particles, indicated by the blue sphere, overlayed on the same tomogram slice. (**b**) Simplified schematic of the angle determination for Env tilting relative to the viral membrane based on the alignment of the masked particles.

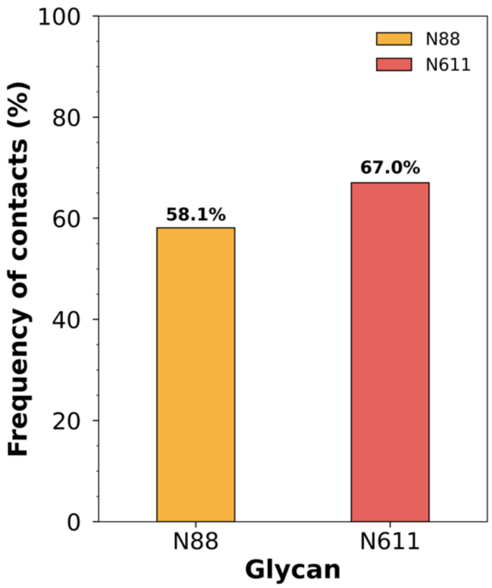

Supplementary Figure 4. Bar plot showing the frequency of contacts between N-glycans N88 (yellow) and N611 (red) with the lipid membrane across all simulations and protomers, expressed as % frames with at least one contact from either is observed. Frames we pooled across five independent MD simulation trajectories. Source data are provided as a Source Data file.

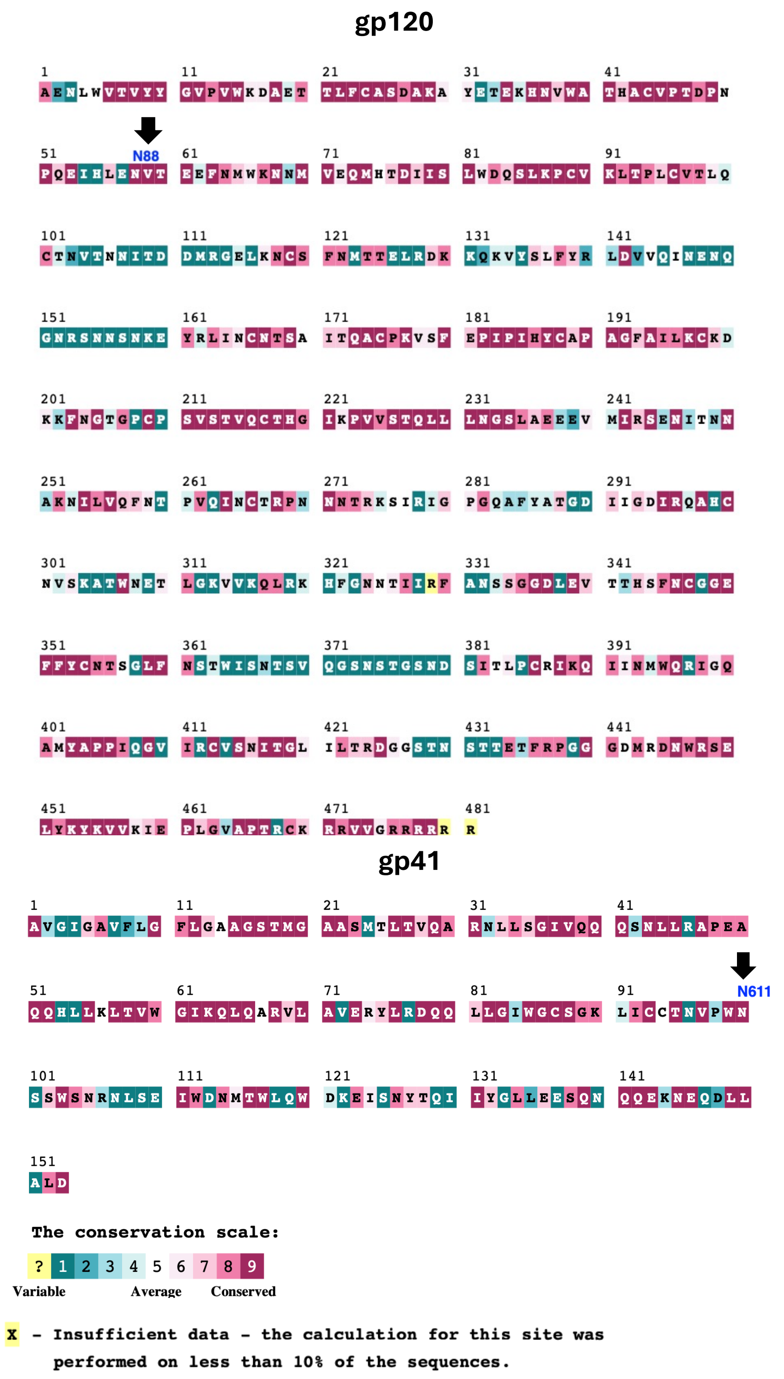

Supplementary Figure 5. gp120 and gp41 sequences highlighting conserved and variable residues across HIV-1 strains.

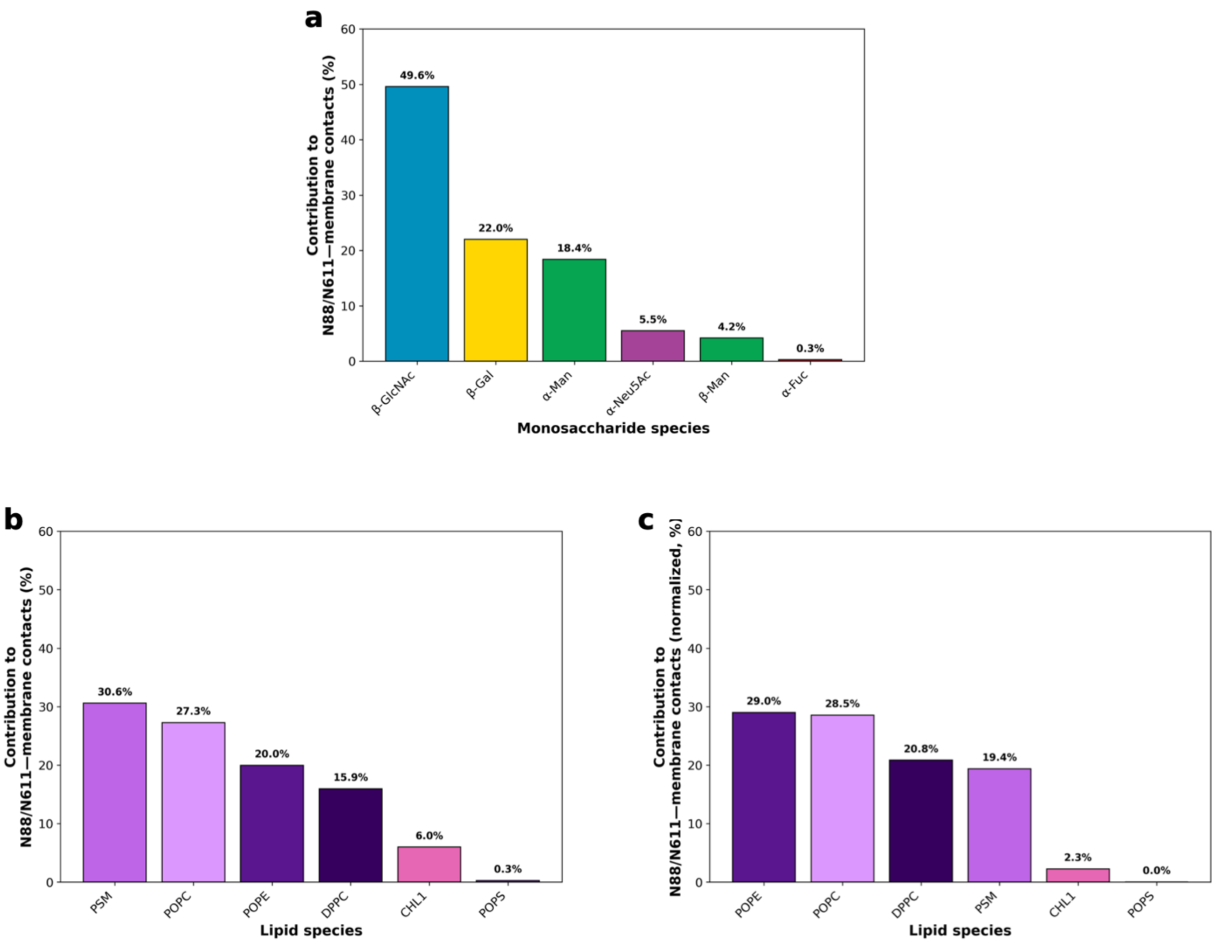

Supplementary Figure 6. Dissection of N88/N611–membrane contacts by molecular species. (**a**) Pooled percent contribution of glycan types to N88/N611–membrane engagement across five independent MD simulation trajectories, colored according to SNFG scheme. (**b**) Pooled percent contribution of lipid classes without abundance normalization. (**c**) Pooled percent contribution of lipid classes normalized by the number of lipid molecules in the outer leaflet (POPC = 150, POPE = 108, DPPC = 120, PSM = 248, CHL1 = 416, POPS = 0). Values above bars indicate the percent contribution of each species. Source data are provided as a Source Data file.

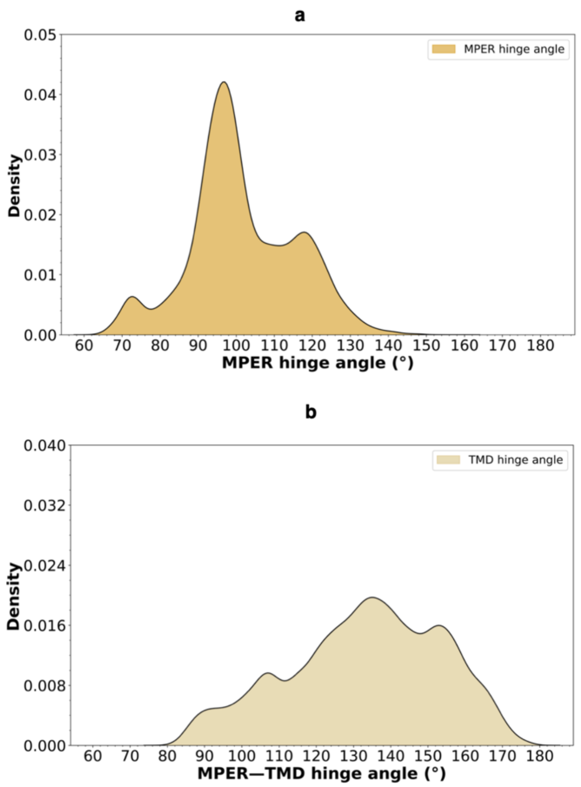

Supplementary Figure 7. Kernel density distributions of membrane-proximal external region (MPER) and membrane-proximal external region-transmembrane domain (MPER–TMD) hinge angles from HIV-1 Env N88A+N611A mutant simulations. (**a**) Distribution of the MPER hinge angle (x-axis, degrees; y-axis, density). (**b**) Distribution of the MPER–TMD hinge angle (x-axis, degrees; y-axis, density). Source data are provided as a Source Data file.

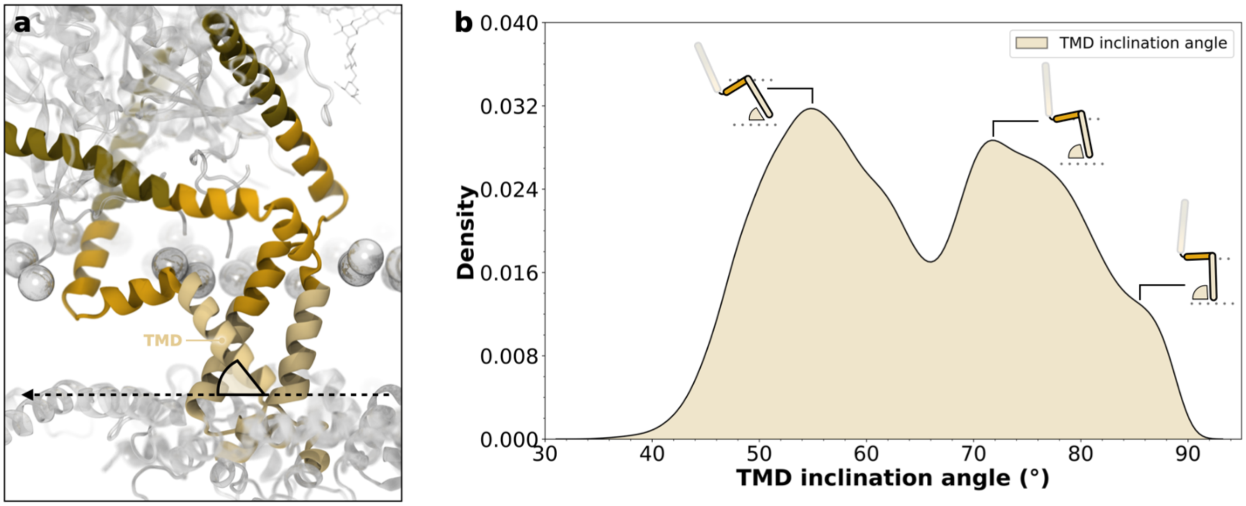

Supplementary Figure 8. (**a**) Structural depiction of the Env trimer's transmembrane domain (TMD) showing the inclination angle relative to the membrane plane. The TMD helices are shown in yellow. The dashed black line indicates the membrane plane, and the angle measurement is illustrated with a black triangle. (**b**) Kernel density estimate of the TMD inclination angle distribution. Source data are provided as a Source Data file.

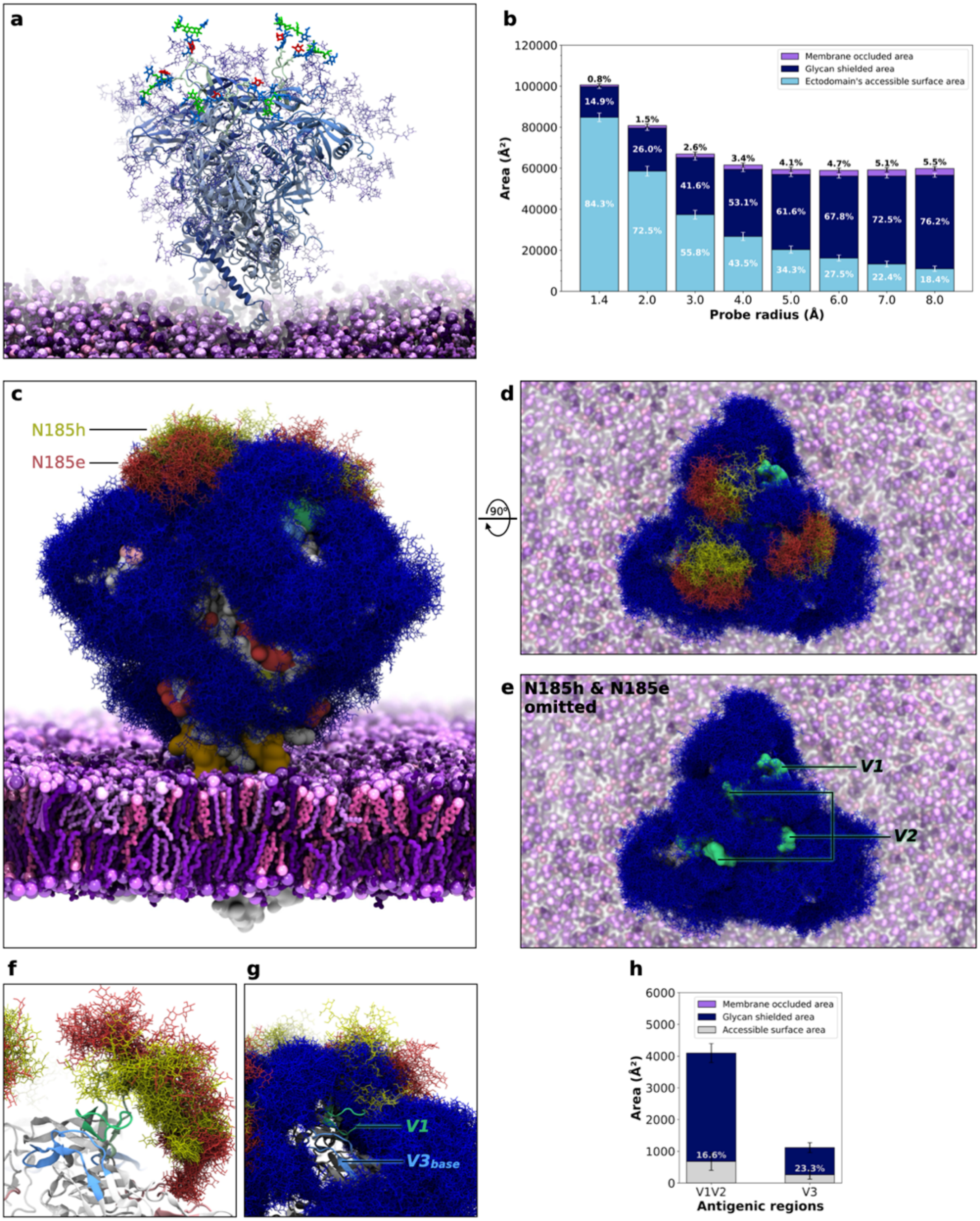

Supplementary Figure 9. Glycan shield of HIV-1 Env including N185e and N185h glycans. (**a**) Molecular representation of the simulated model. N185e/h glycans are colored according to SNFG representation, whereas all the others glycans are shown with thinner blue sticks. (**b**) Quantification of the accessible surface area (cyan), glycan-shielded area (dark blue), and membrane-occluded area (purple) in Å^2 across probe radii ranging from 1.4 Å to 8 Å

(x-axis), with relative percentages indicated within each stacked bar. For each stacked bar, values were pooled across five independent simulation trajectories (n = 5) initiated with distinct velocity seeds. Within each trajectory, surface areas were calculated over 1,051 sampled frames. Error bars indicate the standard deviation of the pooled values across all sampled frames. (**c-e**) Molecular representations of the HIV-1 Env trimer's glycan shield, shown from a side view (**c**) and a top-down views (**d,e**). Glycans are shown as a blue mesh ensemble of overlaid conformations sampled during a 1-µs-long simulation. N185e and N185h N-linked glycans are colored in red and yellow, respectively. The protein is represented by a cyan surface, while the membrane is depicted in purple. (**f, g**) Close-up views of V2 glycans N185e/h glycan shield relative to antigenic regions V1 (green) and V3base (cyan). (**h**) Quantification of Env surface accessibility for the V1V2 and V3 antigenic regions, partitioned into membrane-occluded, glycan-shielded, and accessible fractions using a probe radius of 7.2 Å. For each stacked bar, values were pooled across five independent simulation trajectories (n = 5) initiated with distinct velocity seeds. Within each trajectory, surface areas were calculated over 1,051 sampled frames and three protomers per frame (15,765 total measurements). Error bars indicate the standard deviation of the pooled values across all sampled frames. Source data are provided as a Source Data file.

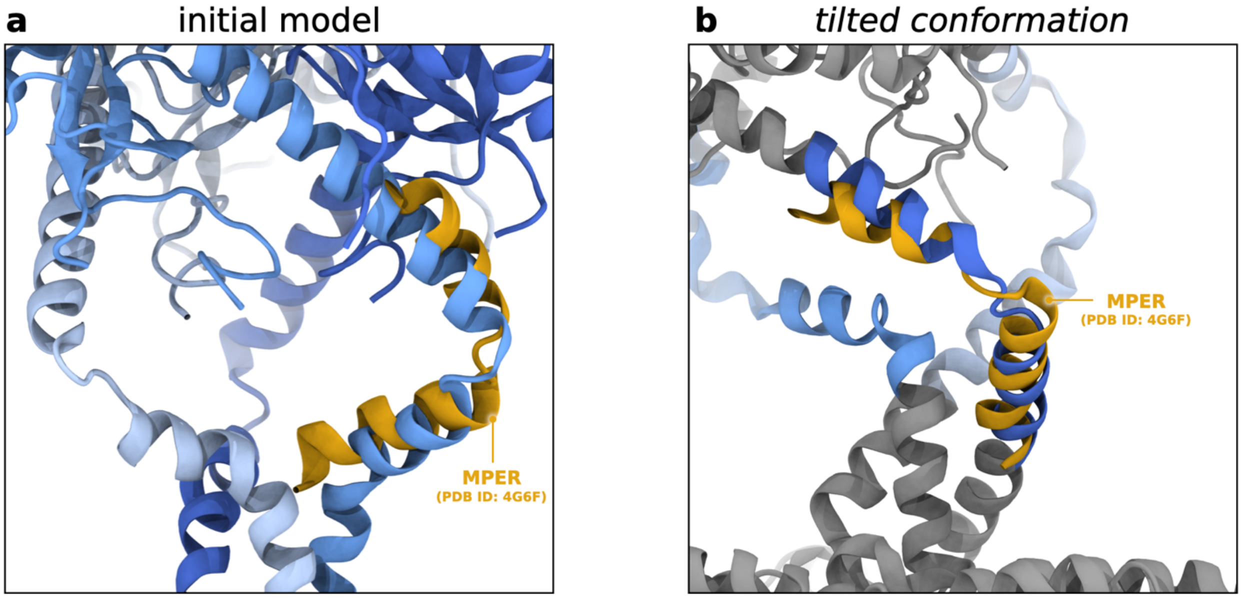

Supplementary Figure 10. Alignment of the membrane-proximal external region (MPER) from the crystal structure of 10E8 Fab in complex with the HIV-1 Env's (MPER) (yellow cartoons, PDB ID: 4G6F[14]) onto the HIV-1 Env's MPER as modeled in this study (blue cartoons). (**a**) Alignment onto the initial model. (**b**) Alignment onto a tilted conformation, resulting in a most exposed conformation of the MPER.

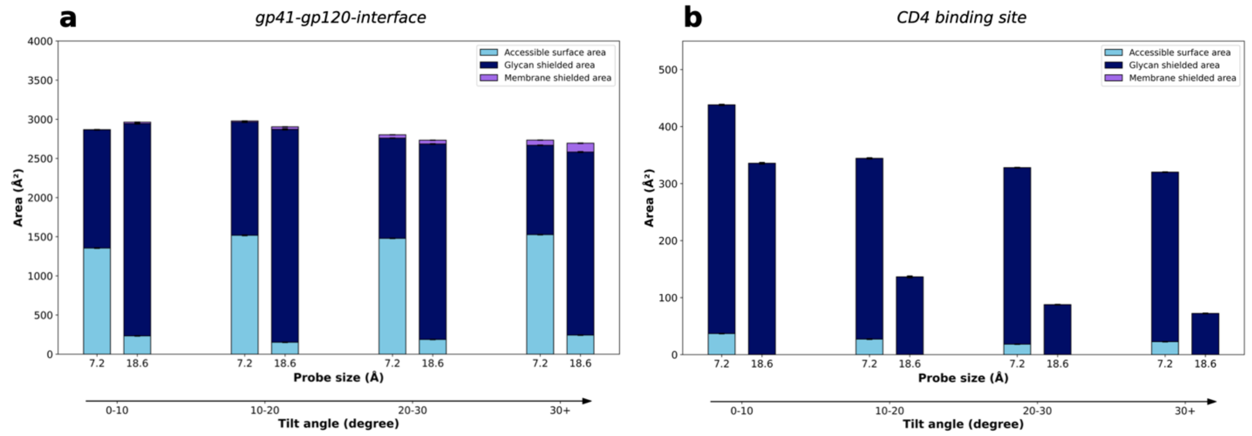

Supplementary Figure 11. Quantification of the membrane-shielded area (purple), glycan-shielded area (dark blue), and accessible surface area (light blue) for the gp41-gp120 interface (**a**) and the CD4 binding site (**b**) across tilt ensembles (0–10°, 10–20°, 20–30°, and 30+°) for probe sizes of 7.2 Å and 18.6 Å. Values were pooled across five independent simulation trajectories initiated with distinct velocity seeds and grouped into four tilt-angle ensembles. For each frame, only the protomer with the maximum accessible surface area for the analyzed epitope was considered. The number of sampled frames contributing to each ensemble was 10,625, 6,085, 23,228, and 12,567, respectively. For each ensemble, mean surface areas were calculated from all sampled frames within that bin. Error bars represent the standard error of the mean (SEM) calculated from the pooled frame-level values in each ensemble. Source data are provided as a Source Data file.

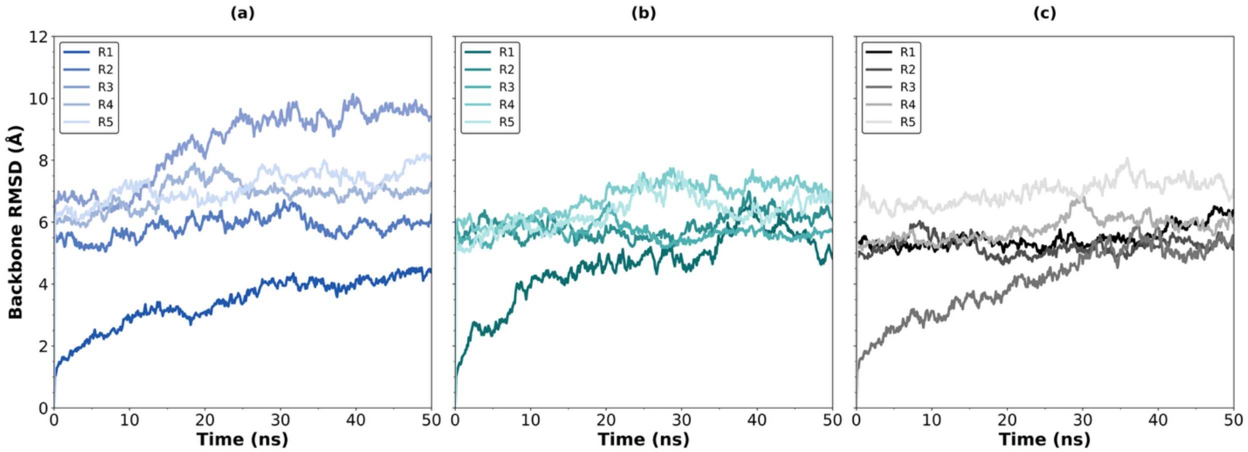

Supplementary Figure 12. Backbone root-mean-square deviation (RMSD) analysis over the first 50 ns of simulation for the three HIV-1 Env full-length models: (a) BG505 Clade A full-length base model, (b) BG505 Clade A full-length base model including N185e and N185h glycans , and (c) BG505 Clade A full-length N88A+N611A mutant model. Source data are provided as a Source Data file.

3. Supplementary Tables

Supplementary Table 1. Glycan compositions for chain A. Glycosylation sites are reported using HXB2 notation.

CHAIN A

#	SITE	TYPE	STRUCTURE	SEQUENCE
G1	N88	FA2	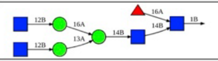	bDGlcNAc(1→2)aDMan(1→6)[bDGlcNAc(1→2)aDMan(1→3)]bDMan(1→4)bDGlcNAc(1→4)<br>[aLFuc(1→6)]bDGlcNAc(1→)PROA-88
G2	N133	FA2	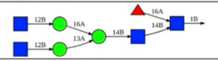	bDGlcNAc(1→2)aDMan(1→6)[bDGlcNAc(1→2)aDMan(1→3)]bDMan(1→4)bDGlcNAc(1→4)<br>[aLFuc(1→6)]bDGlcNAc(1→)PROA-133
G3	N137	FA2G2S2	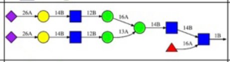	aDNeu5Ac(2→6)bDGal(1→4)bDGlcNAc(1→2)aDMan(1→6)<br>[aDNeu5Ac(2→6)bDGal(1→4)bDGlcNAc(1→2)aDMan(1→3)]bDMan(1→4)bDGlcNAc(1→4)<br>[aLFuc(1→6)]bDGlcNAc(1→)PROA-137
G4	N156	M9	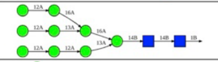	aDMan(1→2)aDMan(1→6)[aDMan(1→2)aDMan(1→3)]aDMan(1→6)<br>[aDMan(1→2)aDMan(1→2)aDMan(1→3)]bDMan(1→4)bDGlcNAc(1→4)bDGlcNAc(1→)PROA-156
G5	N160	M6	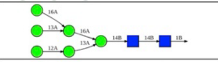	bDGal(1→4)bDGlcNAc(1→2)aDMan(1→3)[aDMan(1→6)<br>[aDMan(1→3)]aDMan(1→6)]bDMan(1→4)bDGlcNAc(1→4)bDGlcNAc(1→)PROA-160
G6	N197	FA2G2S1	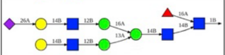	aDNeu5Ac(2→6)bDGal(1→4)bDGlcNAc(1→2)aDMan(1→6)<br>[bDGal(1→4)bDGlcNAc(1→2)aDMan(1→3)]bDMan(1→4)bDGlcNAc(1→4)[aLFuc(1→6)]bDGlcNAc(1→)PROA-197
G7	N234	M9	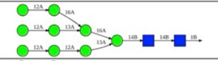	aDMan(1→2)aDMan(1→6)[aDMan(1→2)aDMan(1→3)]aDMan(1→6)<br>[aDMan(1→2)aDMan(1→2)aDMan(1→3)]bDMan(1→4)bDGlcNAc(1→4)bDGlcNAc(1→)PROA-234
G8	N262	M8	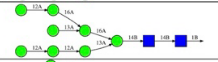	aDMan(1→2)aDMan(1→6)[aDMan(1→3)]aDMan(1→6)<br>[aDMan(1→2)aDMan(1→2)aDMan(1→3)]bDMan(1→4)bDGlcNAc(1→4)bDGlcNAc(1→)PROA-262
G9	N276	Hybrid G1	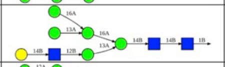	bDGal(1→4)bDGlcNAc(1→2)aDMan(1→3)[aDMan(1→6)<br>[aDMan(1→3)]aDMan(1→6)]bDMan(1→4)bDGlcNAc(1→4)bDGlcNAc(1→)PROA-276
G10	N295	M8	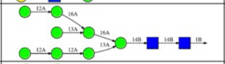	aDMan(1→2)aDMan(1→6)[aDMan(1→3)]aDMan(1→6)<br>[aDMan(1→2)aDMan(1→2)aDMan(1→3)]bDMan(1→4)bDGlcNAc(1→4)bDGlcNAc(1→)PROA-295
G11	N301	M9	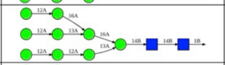	aDMan(1→2)aDMan(1→6)[aDMan(1→2)aDMan(1→3)]aDMan(1→6)<br>[aDMan(1→2)aDMan(1→2)aDMan(1→3)]bDMan(1→4)bDGlcNAc(1→4)bDGlcNAc(1→)PROA-301
G12	N339	M9	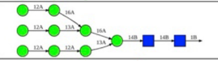	aDMan(1→2)aDMan(1→6)[aDMan(1→2)aDMan(1→3)]aDMan(1→6)<br>[aDMan(1→2)aDMan(1→2)aDMan(1→3)]bDMan(1→4)bDGlcNAc(1→4)bDGlcNAc(1→)PROA-339
G13	N355	FA2	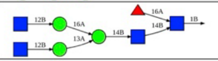	bDGlcNAc(1→2)aDMan(1→6)[bDGlcNAc(1→2)aDMan(1→3)]bDMan(1→4)bDGlcNAc(1→4)<br>[aLFuc(1→6)]bDGlcNAc(1→)PROA-355
G14	N363	M9	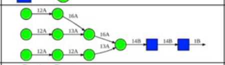	aDMan(1→2)aDMan(1→6)[aDMan(1→2)aDMan(1→3)]aDMan(1→6)<br>[aDMan(1→2)aDMan(1→2)aDMan(1→3)]bDMan(1→4)bDGlcNAc(1→4)bDGlcNAc(1→)PROA-363
G15	N386	M8	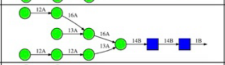	aDMan(1→2)aDMan(1→6)[aDMan(1→3)]aDMan(1→6)<br>[aDMan(1→2)aDMan(1→2)aDMan(1→3)]bDMan(1→4)bDGlcNAc(1→4)bDGlcNAc(1→)PROA-386
G16	N392	M9	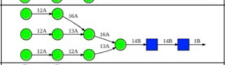	aDMan(1→2)aDMan(1→6)[aDMan(1→2)aDMan(1→3)]aDMan(1→6)<br>[aDMan(1→2)aDMan(1→2)aDMan(1→3)]bDMan(1→4)bDGlcNAc(1→4)bDGlcNAc(1→)PROA-392
G17	N398	M8	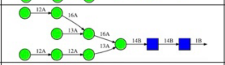	aDMan(1→2)aDMan(1→6)[aDMan(1→3)]aDMan(1→6)<br>[aDMan(1→2)aDMan(1→2)aDMan(1→3)]bDMan(1→4)bDGlcNAc(1→4)bDGlcNAc(1→)PROA-398
G18	N406	FA2G2S1	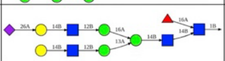	aDNeu5Ac(2→6)bDGal(1→4)bDGlcNAc(1→2)aDMan(1→6)<br>[bDGal(1→4)bDGlcNAc(1→2)aDMan(1→3)]bDMan(1→4)bDGlcNAc(1→4)[aLFuc(1→6)]bDGlcNAc(1→)PROA-406
G19	N411	M8	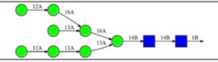	aDMan(1→2)aDMan(1→6)[aDMan(1→3)]aDMan(1→6)<br>[aDMan(1→2)aDMan(1→2)aDMan(1→3)]bDMan(1→4)bDGlcNAc(1→4)bDGlcNAc(1→)PROA-411
G20	N416	M9	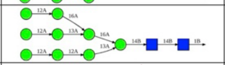	aDMan(1→2)aDMan(1→6)[aDMan(1→2)aDMan(1→3)]aDMan(1→6)<br>[aDMan(1→2)aDMan(1→2)aDMan(1→3)]bDMan(1→4)bDGlcNAc(1→4)bDGlcNAc(1→)PROA-416
G21	N462	FA2	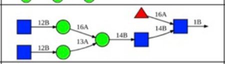	bDGlcNAc(1→2)aDMan(1→6)[bDGlcNAc(1→2)aDMan(1→3)]bDMan(1→4)bDGlcNAc(1→4)<br>[aLFuc(1→6)]bDGlcNAc(1→)PROA-462
G22	N611	A2G2	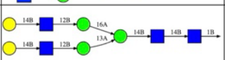	bDGal(1→4)bDGlcNAc(1→2)aDMan(1→6)<br>[bDGal(1→4)bDGlcNAc(1→2)aDMan(1→3)]bDMan(1→4)bDGlcNAc(1→4)bDGlcNAc(1→)PROA-611
G23	N637	FA2G2S1	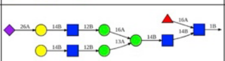	aDNeu5Ac(2→6)bDGal(1→4)bDGlcNAc(1→2)aDMan(1→6)<br>[bDGal(1→4)bDGlcNAc(1→2)aDMan(1→3)]bDMan(1→4)bDGlcNAc(1→4)[aLFuc(1→6)]bDGlcNAc(1→)PROA-637

Supplementary Table 2. Glycan compositions for chain B. Glycosylation sites are reported using HXB2 notation.

CHAIN B

#	SITE	TYPE	STRUCTURE	SEQUENCE
G1	N88	FA2G2S1	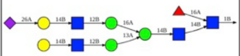	aDNeu5Ac(2→6)bDGal(1→4)bDGlcNAc(1→2)aDMan(1→6)<br>[bDGal(1→4)bDGlcNAc(1→2)aDMan(1→3)]bDMan(1→4)bDGlcNAc(1→4)[aLFuc(1→6)]bDGlcNAc(1→)PROB-88
G2	N133	FA2G2S1	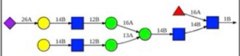	aDNeu5Ac(2→6)bDGal(1→4)bDGlcNAc(1→2)aDMan(1→6)<br>[bDGal(1→4)bDGlcNAc(1→2)aDMan(1→3)]bDMan(1→4)bDGlcNAc(1→4)[aLFuc(1→6)]bDGlcNAc(1→)PROB-133
G3	N137	FA2	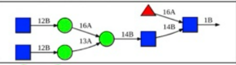	bDGlcNAc(1→2)aDMan(1→6)[bDGlcNAc(1→2)aDMan(1→3)]bDMan(1→4)bDGlcNAc(1→4)<br>[aLFuc(1→6)]bDGlcNAc(1→)PROB-137
G4	N156	M8	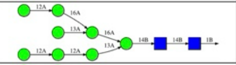	aDMan(1→2)aDMan(1→6)[aDMan(1→3)]aDMan(1→6)<br>[aDMan(1→2)aDMan(1→2)aDMan(1→3)]bDMan(1→4)bDGlcNAc(1→4)bDGlcNAc(1→)PROB-156
G5	N160	M8	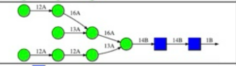	aDMan(1→2)aDMan(1→6)[aDMan(1→3)]aDMan(1→6)<br>[aDMan(1→2)aDMan(1→2)aDMan(1→3)]bDMan(1→4)bDGlcNAc(1→4)bDGlcNAc(1→)PROB-160
G6	N197	FA3	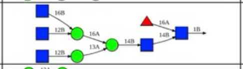	bDGlcNAc(1→6)[bDGlcNAc(1→2)]aDMan(1→6)[bDGlcNAc(1→2)aDMan(1→3)]bDMan(1→4)bDGlcNAc(1→4)<br>[aLFuc(1→6)]bDGlcNAc(1→)PROB-197
G7	N234	M9	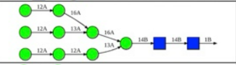	aDMan(1→2)aDMan(1→6)[aDMan(1→2)aDMan(1→3)]aDMan(1→6)<br>[aDMan(1→2)aDMan(1→2)aDMan(1→3)]bDMan(1→4)bDGlcNAc(1→4)bDGlcNAc(1→)PROB-234
G8	N262	M8	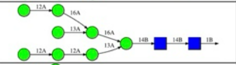	aDMan(1→2)aDMan(1→6)[aDMan(1→3)]aDMan(1→6)<br>[aDMan(1→2)aDMan(1→2)aDMan(1→3)]bDMan(1→4)bDGlcNAc(1→4)bDGlcNAc(1→)PROB-262
G9	N276	Hybrid G1	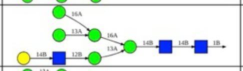	bDGal(1→4)bDGlcNAc(1→2)aDMan(1→3)[aDMan(1→6)<br>[aDMan(1→3)]aDMan(1→6)]bDMan(1→4)bDGlcNAc(1→4)bDGlcNAc(1→)PROB-276
G10	N295	M8	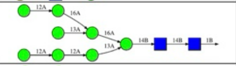	aDMan(1→2)aDMan(1→6)[aDMan(1→3)]aDMan(1→6)<br>[aDMan(1→2)aDMan(1→2)aDMan(1→3)]bDMan(1→4)bDGlcNAc(1→4)bDGlcNAc(1→)PROB-295
G11	N301	M9	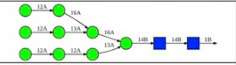	aDMan(1→2)aDMan(1→6)[aDMan(1→2)aDMan(1→3)]aDMan(1→6)<br>[aDMan(1→2)aDMan(1→2)aDMan(1→3)]bDMan(1→4)bDGlcNAc(1→4)bDGlcNAc(1→)PROB-301
G12	N339	M9	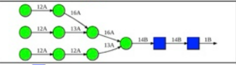	aDMan(1→2)aDMan(1→6)[aDMan(1→2)aDMan(1→3)]aDMan(1→6)<br>[aDMan(1→2)aDMan(1→2)aDMan(1→3)]bDMan(1→4)bDGlcNAc(1→4)bDGlcNAc(1→)PROB-339
G13	N355	FA3	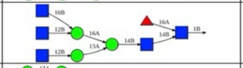	bDGlcNAc(1→6)[bDGlcNAc(1→2)]aDMan(1→6)[bDGlcNAc(1→2)aDMan(1→3)]bDMan(1→4)bDGlcNAc(1→4)<br>[aLFuc(1→6)]bDGlcNAc(1→)PROB-355
G14	N363	M8	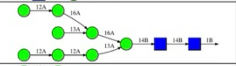	aDMan(1→2)aDMan(1→6)[aDMan(1→3)]aDMan(1→6)<br>[aDMan(1→2)aDMan(1→2)aDMan(1→3)]bDMan(1→4)bDGlcNAc(1→4)bDGlcNAc(1→)PROB-363
G15	N386	M8	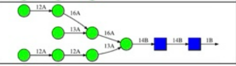	aDMan(1→2)aDMan(1→6)[aDMan(1→3)]aDMan(1→6)<br>[aDMan(1→2)aDMan(1→2)aDMan(1→3)]bDMan(1→4)bDGlcNAc(1→4)bDGlcNAc(1→)PROB-386
G16	N392	M9	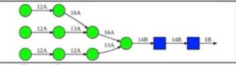	aDMan(1→2)aDMan(1→6)[aDMan(1→2)aDMan(1→3)]aDMan(1→6)<br>[aDMan(1→2)aDMan(1→2)aDMan(1→3)]bDMan(1→4)bDGlcNAc(1→4)bDGlcNAc(1→)PROB-392
G17	N398	M8	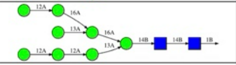	aDMan(1→2)aDMan(1→6)[aDMan(1→3)]aDMan(1→6)<br>[aDMan(1→2)aDMan(1→2)aDMan(1→3)]bDMan(1→4)bDGlcNAc(1→4)bDGlcNAc(1→)PROB-398
G18	N406	FA3G3	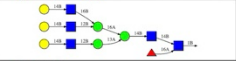	bDGal(1→4)bDGlcNAc(1→6)[bDGal(1→4)bDGlcNAc(1→2)]aDMan(1→6)<br>[bDGal(1→4)bDGlcNAc(1→2)aDMan(1→3)]bDMan(1→4)bDGlcNAc(1→4)[aLFuc(1→6)]bDGlcNAc(1→)PROB-406
G19	N411	M8	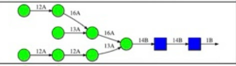	aDMan(1→2)aDMan(1→6)[aDMan(1→3)]aDMan(1→6)<br>[aDMan(1→2)aDMan(1→2)aDMan(1→3)]bDMan(1→4)bDGlcNAc(1→4)bDGlcNAc(1→)PROB-411
G20	N416	M9	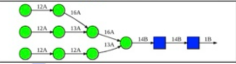	aDMan(1→2)aDMan(1→6)[aDMan(1→2)aDMan(1→3)]aDMan(1→6)<br>[aDMan(1→2)aDMan(1→2)aDMan(1→3)]bDMan(1→4)bDGlcNAc(1→4)bDGlcNAc(1→)PROB-416
G21	N462	FA3	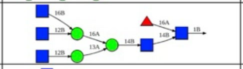	bDGlcNAc(1→6)[bDGlcNAc(1→2)]aDMan(1→6)[bDGlcNAc(1→2)aDMan(1→3)]bDMan(1→4)bDGlcNAc(1→4)<br>[aLFuc(1→6)]bDGlcNAc(1→)PROB-462
G22	N611	A3	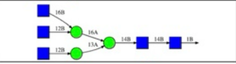	bDGlcNAc(1→6)[bDGlcNAc(1→2)]aDMan(1→6)<br>[bDGlcNAc(1→2)aDMan(1→3)]bDMan(1→4)bDGlcNAc(1→4)bDGlcNAc(1→)PROB-611
G23	N637	FA2	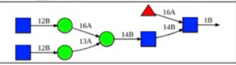	bDGlcNAc(1→2)aDMan(1→6)[bDGlcNAc(1→2)aDMan(1→3)]bDMan(1→4)bDGlcNAc(1→4)<br>[aLFuc(1→6)]bDGlcNAc(1→)PROB-637

Supplementary Table 3. Glycan compositions for chain C. Glycosylation sites are reported using HXB2 notation.

CHAIN C

#	SITE	TYPE	STRUCTURE	SEQUENCE
G1	N88	A2G2	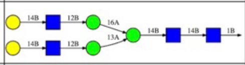	bDGal(1→4)bDGlcNAc(1→2)aDMan(1→6)<br>[bDGal(1→4)bDGlcNAc(1→2)aDMan(1→3)]bDMan(1→4)bDGlcNAc(1→4)bDGlcNAc(1→)PROC-88
G2	N133	A2G2	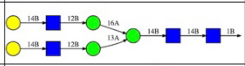	bDGal(1→4)bDGlcNAc(1→2)aDMan(1→6)<br>[bDGal(1→4)bDGlcNAc(1→2)aDMan(1→3)]bDMan(1→4)bDGlcNAc(1→4)bDGlcNAc(1→)PROC-133
G3	N137	FA2	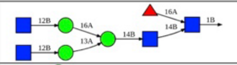	bDGlcNAc(1→2)aDMan(1→6)[bDGlcNAc(1→2)aDMan(1→3)]bDMan(1→4)bDGlcNAc(1→4)<br>[aLFuc(1→6)]bDGlcNAc(1→)PROC-137
G4	N156	M7	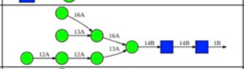	aDMan(1→2)aDMan(1→2)aDMan(1→3)[aDMan(1→6)<br>[aDMan(1→3)]aDMan(1→6)]bDMan(1→4)bDGlcNAc(1→4)bDGlcNAc(1→)PROC-156
G5	N160	M7	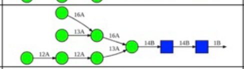	aDMan(1→2)aDMan(1→2)aDMan(1→3)[aDMan(1→6)<br>[aDMan(1→3)]aDMan(1→6)]bDMan(1→4)bDGlcNAc(1→4)bDGlcNAc(1→)PROC-160
G6	N197	FA2G2S2	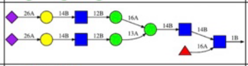	aDNeu5Ac(2→6)bDGal(1→4)bDGlcNAc(1→2)aDMan(1→6)<br>[aDNeu5Ac(2→6)bDGal(1→4)bDGlcNAc(1→2)aDMan(1→3)]bDMan(1→4)bDGlcNAc(1→4)<br>[aLFuc(1→6)]bDGlcNAc(1→)PROC-197
G7	N234	M9	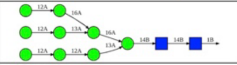	aDMan(1→2)aDMan(1→6)[aDMan(1→2)aDMan(1→3)]aDMan(1→6)<br>[aDMan(1→2)aDMan(1→2)aDMan(1→3)]bDMan(1→4)bDGlcNAc(1→4)bDGlcNAc(1→)PROC-234
G8	N262	M8	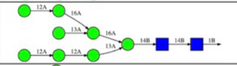	aDMan(1→2)aDMan(1→6)[aDMan(1→3)]aDMan(1→6)<br>[aDMan(1→2)aDMan(1→2)aDMan(1→3)]bDMan(1→4)bDGlcNAc(1→4)bDGlcNAc(1→)PROC-262
G9	N276	Hybrid G1	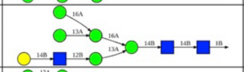	bDGal(1→4)bDGlcNAc(1→2)aDMan(1→3)[aDMan(1→6)<br>[aDMan(1→3)]aDMan(1→6)]bDMan(1→4)bDGlcNAc(1→4)bDGlcNAc(1→)PROC-276
G10	N295	M8	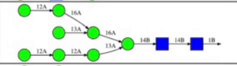	aDMan(1→2)aDMan(1→6)[aDMan(1→3)]aDMan(1→6)<br>[aDMan(1→2)aDMan(1→2)aDMan(1→3)]bDMan(1→4)bDGlcNAc(1→4)bDGlcNAc(1→)PROC-295
G11	N301	M8	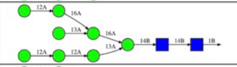	aDMan(1→2)aDMan(1→6)[aDMan(1→3)]aDMan(1→6)<br>[aDMan(1→2)aDMan(1→2)aDMan(1→3)]bDMan(1→4)bDGlcNAc(1→4)bDGlcNAc(1→)PROC-301
G12	N339	M8	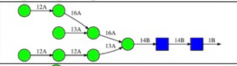	aDMan(1→2)aDMan(1→6)[aDMan(1→3)]aDMan(1→6)<br>[aDMan(1→2)aDMan(1→2)aDMan(1→3)]bDMan(1→4)bDGlcNAc(1→4)bDGlcNAc(1→)PROC-339
G13	N355	Hybrid G1	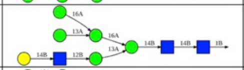	bDGal(1→4)bDGlcNAc(1→2)aDMan(1→3)[aDMan(1→6)<br>[aDMan(1→3)]aDMan(1→6)]bDMan(1→4)bDGlcNAc(1→4)bDGlcNAc(1→)PROC-355
G14	N363	M8	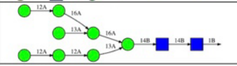	aDMan(1→2)aDMan(1→6)[aDMan(1→3)]aDMan(1→6)<br>[aDMan(1→2)aDMan(1→2)aDMan(1→3)]bDMan(1→4)bDGlcNAc(1→4)bDGlcNAc(1→)PROC-363
G15	N386	M8	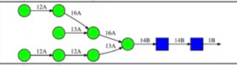	aDMan(1→2)aDMan(1→6)[aDMan(1→3)]aDMan(1→6)<br>[aDMan(1→2)aDMan(1→2)aDMan(1→3)]bDMan(1→4)bDGlcNAc(1→4)bDGlcNAc(1→)PROC-386
G16	N392	M8	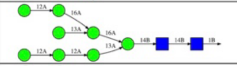	aDMan(1→2)aDMan(1→6)[aDMan(1→3)]aDMan(1→6)<br>[aDMan(1→2)aDMan(1→2)aDMan(1→3)]bDMan(1→4)bDGlcNAc(1→4)bDGlcNAc(1→)PROC-392
G17	N398	M9	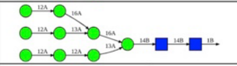	aDMan(1→2)aDMan(1→6)[aDMan(1→2)aDMan(1→3)]aDMan(1→6)<br>[aDMan(1→2)aDMan(1→2)aDMan(1→3)]bDMan(1→4)bDGlcNAc(1→4)bDGlcNAc(1→)PROC-398
G18	N406	FA2	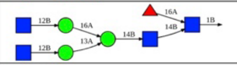	bDGlcNAc(1→2)aDMan(1→6)[bDGlcNAc(1→2)aDMan(1→3)]bDMan(1→4)bDGlcNAc(1→4)<br>[aLFuc(1→6)]bDGlcNAc(1→)PROC-406
G19	N411	M9	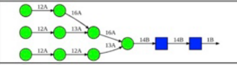	aDMan(1→2)aDMan(1→6)[aDMan(1→2)aDMan(1→3)]aDMan(1→6)<br>[aDMan(1→2)aDMan(1→2)aDMan(1→3)]bDMan(1→4)bDGlcNAc(1→4)bDGlcNAc(1→)PROC-411
G20	N416	M8	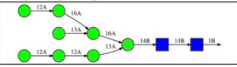	aDMan(1→2)aDMan(1→6)[aDMan(1→3)]aDMan(1→6)<br>[aDMan(1→2)aDMan(1→2)aDMan(1→3)]bDMan(1→4)bDGlcNAc(1→4)bDGlcNAc(1→)PROC-416
G21	N462	FA2G2S2	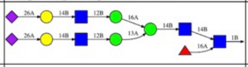	aDNeu5Ac(2→6)bDGal(1→4)bDGlcNAc(1→2)aDMan(1→6)<br>[aDNeu5Ac(2→6)bDGal(1→4)bDGlcNAc(1→2)aDMan(1→3)]bDMan(1→4)bDGlcNAc(1→4)<br>[aLFuc(1→6)]bDGlcNAc(1→)PROC-462
G22	N611	A2G2S2	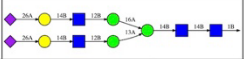	aDNeu5Ac(2→6)bDGal(1→4)bDGlcNAc(1→2)aDMan(1→6)<br>[aDNeu5Ac(2→6)bDGal(1→4)bDGlcNAc(1→2)aDMan(1→3)]bDMan(1→4)bDGlcNAc(1→4)bDGlcNAc(1→)PROC-611
G23	N637	FA2	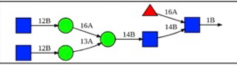	bDGlcNAc(1→2)aDMan(1→6)[bDGlcNAc(1→2)aDMan(1→3)]bDMan(1→4)bDGlcNAc(1→4)<br>[aLFuc(1→6)]bDGlcNAc(1→)PROC-637

Supplementary Table 4. Membrane lipid composition.

Lipid Category	Lipid	Percent Abundance	Outer Leaflet Fraction	Outer Leaflet Count	Inner Leaflet Fraction	Inner Leaflet Count
PC	DPPC	6.32	0.75	120	0.25	30
	POPC	9.48	0.75	150	0.25	46
PE	POPE	21.40	0.25	108	0.75	320
PS	POPS	13.72	0.00	0	1.00	260
SM	PSM	16.51	0.75	248	0.25	82
Chl	CHOL	32.56	0.60	416	0.40	260

Supplementary Table 5. Details of Simulated Systems.

System	Box dimensions (Å x Å x Å)	Total # atoms	Salt Concentration (M)	R1 (ns)	R2 (ns)	R3 (ns)	R4 (ns)	R5 (ns)
Full length Env	242 Å x 242 Å x 252 Å	1297075	0.15	1050	1050	1050	1050	1050
N88A_N611A Env	242 Å x 242 Å x 252 Å	1297278	0.15	1050	1050	1050	1050	1050
Full length Env (N185e and N185h glycosylated)	242 Å x 242 Å x 252 Å	1297007	0.15	1050	1050	1050	1050	1050

Supplementary Table 6. Cryo-ET data collection, refinement and validation statistics

	#1 name (EMDB-75641)
**Data collection and processing**	
Magnification	81000
Voltage (kV)	300
Electron exposure (e–/Å^2)	136
Defocus range (μm)	-3 to -5
Pixel size (Å)	1.561
Symmetry imposed	C3
Initial particle images (no.)	6389
Final particle images (no.)	4396
Map resolution (Å)	20
FSC threshold	0.143
Map resolution range (Å)	N/A
**Refinement**	
Initial model used (PDB code)	MD simulation output from this paper. Only docked in.
Model resolution (Å)	N/A
FSC threshold	
Model resolution range (Å)	N/A
Map sharpening *B* factor (Å^2)	N/A
Model composition	N/A
Non-hydrogen atoms	
Protein residues	
Ligands	
B factors (Å^2)	N/A
Protein	
Ligand	
R.m.s. deviations	N/A
Bond lengths (Å)	
Bond angles (°)	
Validation	N/A
MolProbity score	
Clashscore	
Poor rotamers (%)	
Ramachandran plot	N/A
Favored (%)	
Allowed (%)	
Disallowed (%)	

Supplementary Table 7. Frequency of glycan–membrane contacts.

Glycan/Chain	R1	R2	R3	R4	R5	Average Frequency
**N88/A**	**0**	**0**	**0**	**60.46**	**87.32**	**29.56**
**N88/B**	**32.71**	**0.1**	**51.82**	**11.24**	**82.83**	**35.74**
**N88/C**	**25.07**	**0.08**	**84.66**	**0**	**0.02**	**21.96**
N133/A	0	0	0	0	0	0
N133/B	0	0	0	0	0	0
N133/C	0	0	0	0	0	0
N137/A	0	0	0	0	0	0
N137/B	0	0	0	0	0	0
N137/C	0	0	0	0	0	0
N156/A	0	0	0	0	0	0
N156/B	0	0	0	0	0	0
N156/C	0	0	0	0	0	0
N160/A	0	0	0	0	0	0
N160/B	0	0	0	0	0	0
N160/C	0	0	0	0	0	0
N197/A	0	0	0	0	0	0
N197/B	0	0	0	0	0	0
N197/C	0	0	0	0	0	0
N234/A	0	0	0	3.39	50.05	10.69
N234/B	0.03	0	42.42	0	0	8.49
N234/C	0	0	0.02	0	0	0.004
N262/A	0	0	0	0	0	0
N262/B	0	0	0	0	0	0
N262/C	0	0	0	0	0	0
N276/A	0	0	0	1.3	3.37	0.93
N276/B	0	0	16.31	0	0	3.26
N276/C	0	0	0	0	0	0
N295/A	0	0	0	14.94	0.51	3.09
N295/B	0	0	0	0	0.21	0.04
N295/C	0	0	30.57	0	0	6.11
N301/A	0	0	0	0	0	0
N301/B	0	0	0	0	0	0
N301/C	0	0	0	0	0	0
N339/A	0	0	0	0.06	0	0.01
N339/B	0	0	0	0	0	0
N339/C	0	0	0	0	0	0

N355/A	0	0	0	33.38	19.91	10.66
N355/B	0	0	0	0	0	0
N355/C	0	0	28.55	0	0	5.71
N363/A	0	0	0	0	0	0
N363/B	0	0	0	0	0	0
N363/C	0	0	0	0	0	0
N386/A	0	0	0	0	0	0
N386/B	0	0	0	0	0	0
N386/C	0	0	0	0	0	0
N392/A	0	0	0	0	0	0
N392/B	0	0	0	0	0	0
N392/C	0	0	0	0	0	0
N398/A	0	0	0	29.42	0.84	6.05
N398/B	0	0	1.97	0	0	0.39
N398/C	0	0	10.27	0	0	2.05
N406/A	0	0	0	0	0	0
N406/B	0	0	0	0	0	0
N406/C	0	0	25.54	0	0	5.11
N411/A	0	0	0	2.5	0	0.5
N411/B	0	0	0	0	0	0
N411/C	0	0	26.21	0	0	5.24
N416/A	0	0	0	0.01	0	0
N416/B	0	0	0	0	0	0
N416/C	0	0	12.58	0	0	2.52
N462/A	0	0	0	1.1	1.54	0.53
N462/B	0	0	0	0	0	0
N462/C	0	0	0.7	0	0	0.14
**N611/A**	**2.19**	**0.96**	**0.13**	**73.3**	**92.73**	**33.86**
**N611/B**	**66.24**	**0.63**	**89.43**	**0**	**0.01**	**31.26**
**N611/C**	**0.48**	**0.56**	**1.3**	**48.39**	**62.72**	**22.69**
N637/A	0	0	0	39.23	9.99	9.84
N637/B	0.17	0	41.71	0	0	8.38
N637/C	0	0	0	0	0	0

Supplementary Table 8. HIV-1 Env's major immunogenic regions. Residue masks are provided using the UniProt numbering scheme, which is consistent with the residue numbering of the Env model simulated in this study. For conversion to HXB2 notation, please refer to the alignment in Figure S1. Glycans are reported using HXB2 notation.

Epitope	Antibody	Residue masks	Reference
**gp120-gp41 interface**	PGT151	46–56, 480–494, 503–511<br>glycan N611	56
	8ANC195	14–19, 59–64, 207–209, 580–584, 596–606	57
	35O22	59–64, 207–209, 586–593, 596–603<br>glycan N88	58
**V1V2**	PGT145	94–96, 121–124, 127–131,<br>3 x glycan N160	59
	PGDM1400	94–96, 121–124, 127–131,<br>3 x glycan N160	60
	PG9	127–135,<br>glycan N156, glycan N160	61
	PG16	127–135, 151–157,<br>glycan N156, glycan N160	62
**V3**	PGT121<br>(V3base glycan supersite +V1)	292–299 and V1's 104–107,<br>glycan N332, glycan N137	59
	BG18 (V3base glycan supersite +V1)	292–299,<br>glycan N332, glycan N137	63
	M4008_1<br>(V3crown)	271–285, 291–293	64
**CD4 binding site**	VRC01	333–341, 397–400, 423–430, 435–44,<br>glycan N276	65
	CH103	333–341, 397–400, 423–430, 435–443,<br>glycan N276	66
	N6	333–341, 397–400, 423–430, 435–443,<br>glycan N276	67
**Fusion Peptide**	N123-VRC34.01	480–494 (HXB2: 512–526)	68
	PGT151	480–494 (HXB2: 512–526)	56
**Silent Face**	VRC-PG05	260–263, 303–306, 413–416,<br>glycan N262, glycan N295, glycan N448	69
	SF12	27–29, 183–184, 222, 260–263, 413–416,<br>glycan N262, glycan N295, glycan N448	70
**MPER**	10E8	639–651 (HXB2: 671–683)	14
	4E10	639–651 (HXB2: 671–683)	71
	Z13e1	634–645 (HXB2: 666–677)	72
	2F5	624–639 (HXB2: 656–671)	73

Supplementary Table 9. Glycan compositions at N185e and N185h glycosylation sites.

#	SITE	Chain	TYPE	STRUCTURE	SEQUENCE
GAE	N185e	A	FA2	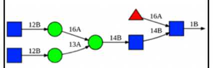	bDGlcNAc(1→2)aDMan(1→6)[bDGlcNAc(1→2)aDMan(1→3)]bDMan(1→4)bDGlcNAc(1→4)<br>[aLFuc(1→6)]bDGlcNAc(1→)PROC-137
GBE	N185e	B	FA2	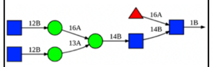	bDGlcNAc(1→2)aDMan(1→6)[bDGlcNAc(1→2)aDMan(1→3)]bDMan(1→4)bDGlcNAc(1→4)<br>[aLFuc(1→6)]bDGlcNAc(1→)PROC-137
GCE	N185e	C	FA3	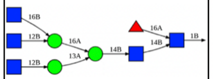	bDGlcNAc(1→6)[bDGlcNAc(1→2)]aDMan(1→6)<br>[bDGlcNAc(1→2)aDMan(1→3)]bDMan(1→4)bDGlcNAc(1→4)<br>[aLFuc(1→6)]bDGlcNAc(1→)PROB-197
GAH	N185h	A	FA2	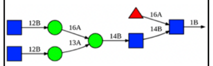	bDGlcNAc(1→2)aDMan(1→6)[bDGlcNAc(1→2)aDMan(1→3)]bDMan(1→4)bDGlcNAc(1→4)<br>[aLFuc(1→6)]bDGlcNAc(1→)PROC-137
GBH	N185h	B	FA3	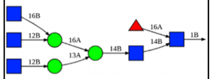	bDGlcNAc(1→6)[bDGlcNAc(1→2)]aDMan(1→6)<br>[bDGlcNAc(1→2)aDMan(1→3)]bDMan(1→4)bDGlcNAc(1→4)<br>[aLFuc(1→6)]bDGlcNAc(1→)PROB-197
GCH	N185h	C	FA2	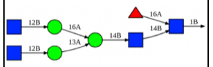	bDGlcNAc(1→2)aDMan(1→6)[bDGlcNAc(1→2)aDMan(1→3)]bDMan(1→4)bDGlcNAc(1→4)<br>[aLFuc(1→6)]bDGlcNAc(1→)PROC-137
