# Supplementary material for: N-Glycans modulate tilting of HIV-1 envelope glycoprotein
Source: Nat Commun. 2026 Apr 15;17:5206. doi: 10.1038/s41467-026-71874-2 (PMC13254402; doi:10.1038/s41467-026-71874-2)
Supplement: Supplementary file 2 — Description of Additional Supplementary File [file 41467_2026_71874_MOESM2_ESM.pdf]

The Description of Additional Supplementary Files

Supplementary Data 1. Archive of MD input files and PDB files containing the initial, equilibrated and final coordinates for all three HIV-1 Env models.

Supplementary Movie 1. All-atom MD simulation of the full-length glycosylated HIV-1 Env trimer embedded in a biologically relevant lipid bilayer. ~1 µs of simulation is shown, illustrating significant tilting of the Env trimer relative to the membrane normal. N-linked glycans are depicted as dynamic blue meshes, the protein backbone is shown in light blue, and the viral membrane is represented with purple van der Waals (vdW) spheres.

Supplementary Movie 2. All-atom MD simulation of the glycosylated HIV-1 Env mutant trimer (N88A+N611A) embedded in a biologically relevant lipid bilayer. ~1 µs of simulation is shown, highlighting the reduced tilting motion of the mutant trimer compared to the wild type. The Env trimer adopts a more upright and less dynamic conformation. N-linked glycans are depicted as dynamic blue meshes, the protein backbone is shown in light blue, and the viral membrane is represented with purple van der Waals (vdW) spheres.
