# Supplementary figures and images for: N-Glycans modulate tilting of HIV-1 envelope glycoprotein
Source: Nat Commun. 2026 Apr 15;17:5206. doi: 10.1038/s41467-026-71874-2 (PMC13254402; doi:10.1038/s41467-026-71874-2)

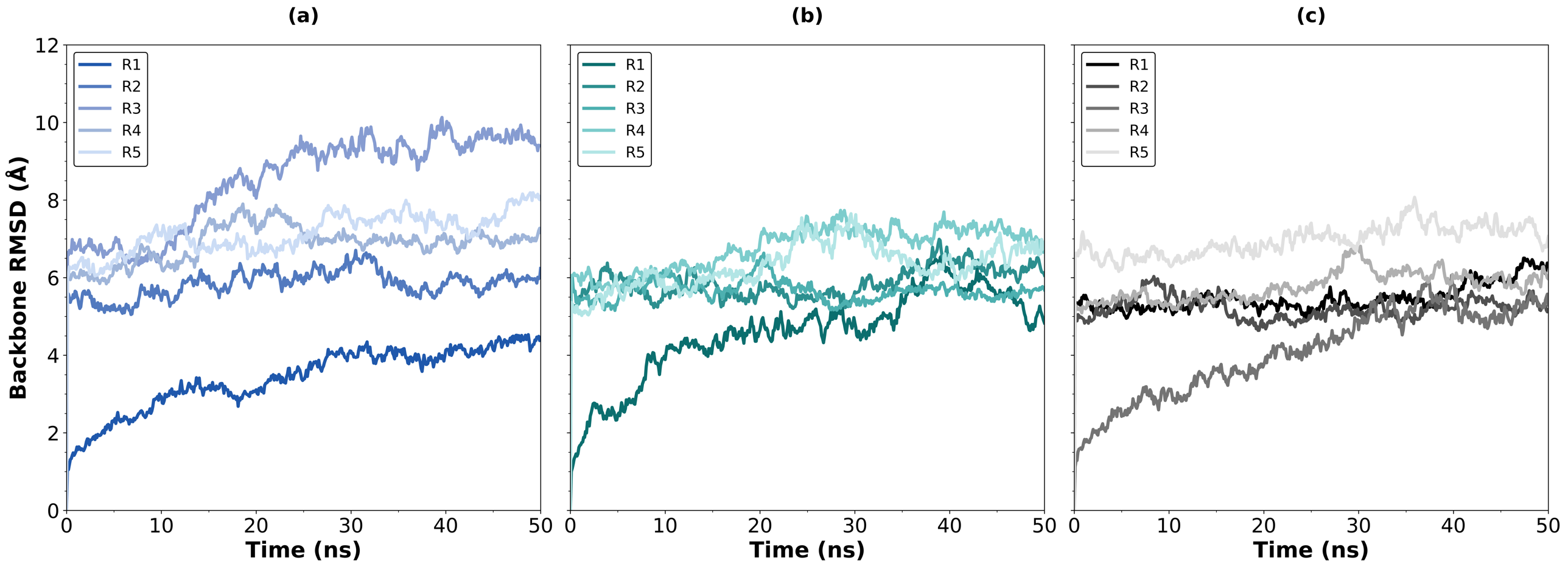

Supplement: Supplementary file 8 — Source Data [file 41467_2026_71874_MOESM8_ESM.zip › source_data/2_Supplementary_Figures/7_FigS12/RMSD_first_50ns_three_panels_elegant.png]
