# Supplementary material for: N-Glycans modulate tilting of HIV-1 envelope glycoprotein
Source: Nat Commun. 2026 Apr 15;17:5206. doi: 10.1038/s41467-026-71874-2 (PMC13254402; doi:10.1038/s41467-026-71874-2)
Supplement: Supplementary file 7 — Transparent Peer Review file [file 41467_2026_71874_MOESM7_ESM.pdf]

nature portfolio

Peer Review File

N-Glycans Modulate Tilting of HIV-1 Envelope Glycoprotein

Corresponding Author: Professor Rommie Amaro

This file contains all reviewer reports in order by version, followed by all author rebuttals in order by version.

Version 0:

Reviewer comments:

Reviewer #1

(Remarks to the Author)
Shehata et al. construct and simulate a full-length, fully glycosylated model of the HIV-1 Env trimer in the context of membrane. In specific, they construct an Env trimer with modeled membrane-interactive region, add glycosylation at 69 sites (23 per protomer), add cysteine palmitoylation, and embed the model in an all-atom membrane bilayer. They then perform microsecond-long MD simulations (with 5 independent replicas) quantifying motions, especially trimer tilt angle, as they find substantial tilts of ~30 degrees in most cases. They use cryo-ET on eVLPs to experimentally visualize Env tilting. They also quantify glycan-membrane contacts, finding glycans 88 and 611 to dominate. Using kernel density estimates, they analyze the distribution of MPER and MPER-TMD hinge angles. Lastly, they assess the glycan shield effectiveness in occluding underlying protein residues, as well as the ability of Env tilting to expose the MPER.

Overall, a very nice paper demonstrating by molecular dynamics and by electron microscope that tilting of the HIV-1 Env trimer in membranes is more pronounced than previously anticipated.

One substantial issue: the initial model. On line 124, the manuscript states that the model includes the ectodomain (residues 631-662) from PDB ID: 5T3A (reference 23). Likely there is a typo, and this should be (residues 31-662). The more critical issue is that the PDB ID selected, 5T3A has a mistake in the register of its final helix (off by 1 residue). This mistake was first incorporated into the 4TVP structure (Pancera et al. 2014 – the first Env trimer structure with gp41 coordinates – and propagated through several other structures until it was corrected in the Stewart-Jones et al. structures that were published in Cell 2016. The critical point is that the alignment of sequence and structure are incorrect in this critical region. The authors should either correct the model and repeat the simulations or - if that is not possible - then the authors should at least comment on how the incorrect model impacts their results, especially the critical MPER and MPER-TMD hinge angles as well as the tilting overall.

Also, for some reason they've only added two of the four conserved gp41 glycans, leaving out the conserved N-linked glycans at residues 618 and 625. While Cao et al. (cited ref. 10) were unable to resolve these two glycans in viral Env, they did resolve in viral Env (from JR-FL and B41, so they are likely complex glycans observed elsewhere), and these two glycans should be added to any fully glycosylated Env trimer.

The gp41 glycosylation is critical as the authors make a point of tilt being controlled by 88/611 glycans, and it makes one wonder what result they would have if they had also added the conserved glycans that are at residues 618 and 625.

In light of the issues with the model, the initial and final PDBs should be provided as supplemental files so that they can be analyzed by the field if further questions arise (if space is an issue, it would be fine to provide coordinates for only protein/glycan portions of the model, leaving out the solvent/membrane).

Other issues the authors should address:

1. Please cite Lee et al., 2024 Comput Struct Biotechnol J., as a prior study that used molecular dynamics to investigate a fully glycosylated HIV-1 Env trimer.
2. Please change "Conformational Plasticity" in title and text to "Tilting in Membrane"; the major alteration in structure is the relative tilt of the Env trimer, not its altered folding/conformation. There are other studies such as the Lee et al. 2024, which address altered folding/conformation, so good to separate these studies and to clarify for readers that it is mainly the tilt that is altered, not the folding of the Env protomer.

3. The authors should comment on the impact of the two missing gp41 glycans at 618/625 on their results.
4. The implications of tilt and MPER-based neutralization are fascinating; it was previously reported by Rantalainen et al., Cell Reports that MPER targeting antibodies tilt the Env trimer in relation to the bilayer; please cite this prior structural study carried out in lipid nanodiscs.
5. I like the attempt to assess the glycan shield's effectiveness. Similar studies have been carried out including by GLYCO (Lee et al., 2022 Bioinformatics) with epitope exposure calculated in Chuang et al., 2019 Structure. These prior investigations should be cited.
6. References 62 and 85 are the same.
7. The authors should also comment on the impact to the glycan shield of leaving off glycans 185e and 185h.
8. There has been some debate on "state 1" identified by smFRET by Mothes and colleagues; the authors should comment on whether or not a tilted Env conformation might account for "state 1".

Reviewer #2

(Remarks to the Author)
The paper by Shehata and colleagues reports a very interesting and timely study of the multiplicity of effects that glycans have on protein functions, dynamics, and immune-recognition mechanisms.
Specifically, the authors study the Envelope protein of HIV in its fully glycosylated and membrane embedded form at atomistic detail.
One very interesting aspect of this paper is that, while the systems and simulations are highly complex, the authors make an effort to reconnect their results to experimental information.
In this context, the inclusion of CryoET data is very interesting, and shows how simulations and experiments should converge to provide biologically relevant information.
The system is challenging and the simulations are carried out in a technically sound way. I would move some of the details on Glycosylation modeling to the main text instead of leaving that in the SI.
Overall, the paper is very well conducted and well written. This group has already given significant contributions in this area and this is certainly another great example.
The interesting part of the paper is that they show the multiple personalities of sugars, not only as shield but also as conformational modulators and interactors with the membrane.
The visualization of the Ab-binding epitope is interesting and shows that these approaches could actually be used in structural vaccinology.

I suggest that the authors make an additional effort to make their discussion and overall concepts more general: They could expand the discussion to other cases where glycans are shown to modulate the dynamics also of non-viral proteins, with an impact on their ability to bind drugs, for instance.
In terms of the epitope discovery/ab-binding part, the authors could also discuss some of the limitations of their approach (MD simulations may be demanding in terms of time and computing time requests) and whether it would be amenable to integration with other methods for epitope prediction.

Overall, I think this is an excellent paper that may attract attention from different communities, not necessarily only the ones linked to simulations.

Reviewer #3

(Remarks to the Author)
I co-reviewed this manuscript with one of the reviewers who provided the listed reports. This is part of the Nature Communications initiative to facilitate training in peer review and to provide appropriate recognition for Early Career Researchers who co-review manuscripts.

Reviewer #4

(Remarks to the Author)
Sheeta et al. present a study combining microsecond-long, all-atom MD simulations with cryo-ET to investigate the conformational flexibility of the full-length, fully glycosylated HIV-1 Env glycoprotein within virus-like particles (VLPs) in a native membrane context. A key finding is the identification of tilting motions of Env relative to the membrane and the proposed functional role of two N-linked glycans (N88 and N611) in modulating this flexibility. The combined computational and experimental approach provides valuable insights into Env's structural plasticity and its potential implications for immune recognition. The technical execution is strong. However, the distinct contribution of the study is substantially reduced by prior work, particularly the study by Prasad et al. (Cell, 2022). More critically, there is a significant shortcoming in the current simulation system. Several studies have reported that the C-terminal tail (CT) of HIV-1 Env GP41 interacts with the matrix protein (MA), and this interaction is likely to influence the tilting motion of Env. Recently, the mature MA lattice structure was resolved by the Briggs group. Therefore, it is imperative to incorporate the MA lattice into the molecular dynamics (MD) simulation system to more accurately reflect the biological context.

Major points
p.1 While cryo-ET was performed, no experimental perturbations such as analyses of glycan deletion mutants were carried out to directly assess the individual or combined roles of N88 and N611 in Env tilting. Without such targeted controls, the proposed functional relevance of these glycans remains a plausible but unverified interpretation. On the other hand,

although N611A and N88A mutants were reportedly generated (according to the supplementary methods), their subtomograms were pooled with wild-type data solely to increase particle numbers, without independent analysis. This approach weakens the central claim that N88 and N611 modulate Env tilting, as it lacks direct experimental validation. If these glycans do affect tilting, mixing mutant and WT particles could bias the structural averages and tilt distributions, thereby compromising the reliability of the conclusions.

p.2 GP41 CT of Env interacts with the MA lattice beneath the membrane, which likely affects the Env dynamics and tilting. The simulation system should include the MA lattice to be closer to the native condition.

p.3 Oversimplified membrane model in MD simulations. The MD simulations model the membrane as flat and tensionless, whereas authentic HIV-1 membranes are expected to be highly curved and heterogeneous. Such membrane properties could significantly impact Env tilting dynamics. Although the manuscript briefly mentions curvature, it underestimates its potential effects. A more thorough discussion of the limitations of the current membrane model, and an explanation of how membrane curvature and tension might alter the results, would be highly valuable.

Minor points
p.3. The manuscript repeats background information (e.g., glycan shielding, immune evasion) in several sections. For example, statements such as, "Understanding the strategies that viruses employ to evade the host immune response is crucial for designing effective vaccines. Among these, the glycan shield stands out as one of the most effective mechanisms utilized by class I viral fusion glycoproteins, including HIV-1 Env, to thwart the humoral response. Approximately half of the molecular weight of Env is derived from N-glycans, which are strategically distributed across the ectodomain," appear multiple times or convey overlapping content. The authors should carefully revise the manuscript to eliminate such redundancies and improve the overall clarity, and readability of the text.

p.4. Include a clear discussion of limitations and outline what experimental work would be necessary to validate the proposed mechanism.

p.5. The authors state that “This discovery builds on prior evidence highlighting the critical role of N88 and N611 glycans in shaping Env’s antigenicity.” However, to be considered a discovery, such a claim requires direct experimental validation.

Version 1:

Reviewer comments:

Reviewer #1

(Remarks to the Author)
In their manuscript entitled, N-Glycans Modulate Tilting of HIV-1 1 Envelope Glycoprotein, Shehata et al. analyze a fully glycosylated full-length model of the HIV-1 Env trimer and find substantial tilting.

In my prior review of the manuscript, I noted that there might be inaccuracies in the model related to the fact that many historical Env trimers did not define accurately the final C-terminal residues of the 664 model, such as PDB 5T3Z, which served as an initial template for their molecular dynamics. The authors have done a decent job providing caveats to their model. However, the discussion of the model construct and analyze is unclear: do the starting coordinates have the incorrect C-terminus of 4TVP and many other models including PDB 5T3Z, or the corrected C-terminus of the latter structures (e.g., from the Stewart-Jones Cell 2016 manuscript)? I accept that modeling of trimers is still being experimentally resolved and am okay with the authors’ caveats and model; however, they should make clear to readers the register of the C-terminal region around reside 664 and to which PDB it most closely aligns. (Likewise, in their Supplementary Fig. 10, the authors should add a few residues numbers on aligned peptide and model; it looks like there is decent overall 2nd structural similarity, but authors need to clarify if sequence register is preserved, which residue numbering should help to address.)

In terms of the glycans on the model, the authors state in their rebuttal that “A key source was indeed Cao et al. (2018) (https://doi.org/10.1038/s41467-018-06121-4), who compared glycan occupancy across multiple Env clades and constructs. Importantly, in the BG505 viral Env, they found that glycosylation sites N618 and N625 were not occupied, as shown in their Figure 2b.”

But this is not correct.

In specific, when looking at Cao et al. Figure 2b, four BG505 constructs are shown, three membrane associated (BG505 dCT N332, BG505 dCT T332, and BG505 viral Env) and one soluble (BG505 SOSIP.664). The two dCT are membrane associated, but missing the intracellular region. Notable, glycans N618 and N625 are fully present on both of the dCT versions, and for the BG505 viral Env, no bar graph is provided. In the legend to Fig. 2b, Cao eta l. state: “… no bar graph indicates the site was not observed in LC/MS.” This means that there is no data on the site, but not that they are unoccupied. For example, on the soluble BG505 construct, there is substantial non-occupancy shown in the LC/MS data (that is, non-occupancy and no data are two very different results).

Importantly, this means that contrary to what the Shehata et al. state in their rebuttal, Cao et al. provide evidence that glycans 618 and 625 are glycosylated on membrane-bound Env trimer (2 out of 3 samples) and for the 3rd sample, they did not

provide any data on whether these two glycans were occupied or not.

The authors do highlight a number of other references with soluble BG505 Env trimer. However, a major finding of the Cao 2018 paper is that the primary difference between soluble and membrane-bound trimers relates to the occupancy of gp41 glycans, with the supposition that in the soluble context, translation is different, and this alters gp41 glycosylation. Thus, these other references all cite soluble BG505 constructs, which are not relevant to the discussion of gp41 in the membrane-bound context.

Bottom line: Cao et al. were able to see glycans N618 and N625 on virion-associated trimers from other clades, as well as on two membrane-associated samples from BG505 though not a third, for which Cao et al. provide no data.

Shehata et al. really should including glycans N618 and N625 on their model.

The problem is that Shehata et al. highlight as a major point of their analysis two other glycans, N88 and N611, as playing a critical role in modulating Env tilting. Similar to glycans N88 and N611, glycans N618 and N625 are located on the membrane-proximal region of Env trimer and would therefore be expected to influence membrane interactions/tilting.

I'm okay with some inaccuracies in the model (e.g. 664 register), but if Shehata et al. are highlighting the impact of two glycans – they really need to make sure that they have the glycosylation for the 5 gp41 glycans on the membrane-proximal region correct, if they want to make conclusions about the impact of glycosylation on tilting.

Reviewer #2

(Remarks to the Author)
This is a very strong paper with a technically elegant approach encompassing cryoEM and comp biology.
I was already very supportive of it in the first submission.
Now I see that the authors have run further analyses and went to great lengths to improve their paper in response to the other reviewers.
I am strongly in favor of the publication of this paper.

Reviewer #3

(Remarks to the Author)
I co-reviewed this manuscript with one of the reviewers who provided the listed reports. This is part of the Nature Communications initiative to facilitate training in peer review and to provide appropriate recognition for Early Career Researchers who co-review manuscripts.

Reviewer #4

(Remarks to the Author)
The authors have submitted a carefully revised manuscript that addresses some of the points raised in the previous review. They clearly explain how their work differs from Prasad et al. (Cell, 2022), include five new microsecond-scale MD simulations of the N88A/N611A mutant, and re-analyze the cryo-ET data using wild-type-only particles. These additions strengthen the mechanistic interpretation of the study. The limitations related to the absence of the MA lattice and membrane curvature are now acknowledged and discussed in greater detail. However, the experimental validation of the glycan effects remains lacking, which is a significant gap given the central focus of the manuscript.
While the manuscript is technically sound, the study relies entirely on simulations without experimental validation. As such, the central claim remains hypothetical. Although the additional simulations strengthen the hypothesis to some extent, without experimental support, the impact of the findings remains limited.

Version 2:

Reviewer comments:

Reviewer #1

(Remarks to the Author)
In "N-Glycans Modulate Tilting of HIV-1 Envelope Glycoprotein", Shehata et al. describe a microsecond-long, all-atom molecular dynamics simulations of a full-length, glycosylated Env glycoprotein embedded in a biologically relevant lipid bilayer. They find substantial tilting, an interesting result – and that this tilting depends on two membrane-proximal N-linked glycans at residues N88 and N611.

However, of the five conserved glycans on gp41, Shehata et al. removed two, at sites N618 and N625. These glycans tend to be underglycosylated on soluble HIV-1 Env, but on virion associated HIV-1 Env, their glycosylation was unclear, as Cao et al. 2018 were unable to obtain data on them – although Cao et al. did see that N618 and N625 were present in all other clades of HIV-1 that they examined.

I'm okay with publishing the current paper in which N618 and N625 are missing, but the authors need to make clear to the readers that these two glycans were removed from the molecular dynamics – and these glycans are known to be present on all other clades. Bottom line: it is critical for the authors need to make clear to readers that these gp41 glycans (N618 and N625 - which are present in all other non-A clades) were missing in the molecular dynamics.

Currently, the only place in the manuscript that this is noted is buried in the methods (lines 585-589), which will not be seen by most readers.

It is critical for the authors to note in the first paragraph of the results that the simulation is missing two of the five gp41 glycans – that these glycans, at N618 and N625, are present in all other non-clade A strains of HIV-1 – and thus these results may be specific to clade A BG505, missing these two conserved glycans.

Also, in the discussion, the authors should highlight for readers the absence of these two glycans, and the likely impact that their presence might have on the tilting that they observed.

Reviewer #3

(Remarks to the Author)
I co-reviewed this manuscript with one of the reviewers who provided the listed reports. This is part of the Nature Communications initiative to facilitate training in peer review and to provide appropriate recognition for Early Career Researchers who co-review manuscripts.

Reviewer #4

(Remarks to the Author)
"I appreciate the authors' clarification that glycan-specific functional experiments, especially at sites such as N88 and N611, would represent a substantial and independent line of work and are beyond the scope of this manuscript. That said, I would like to stress that such experiments will ultimately be essential for placing the proposed glycan-mediated mechanism on firm experimental and functional footing."

The current cryo-ET data support the global tilting behaviour of Env, but due to the relatively low resolution of the subtomogram averages, they cannot resolve individual glycans or detailed MPER/TMD conformations. As a result, the specific roles of N88 and N611 still remain simulation-derived and hypothesis-generating, rather than functionally demonstrated.

Version 3:

Reviewer comments:

Reviewer #1

(Remarks to the Author)
The authors have responded robustly to my last set of comments, with clear caveat in the first paragraph to the results highlighting the absence of glycans N618 and N625 along with added discussion relating to the presence or absence of these two glycans. With these added edits, readers are now appropriately informed of the limitations of the clade a BG505 model.

Reviewer #3

(Remarks to the Author)
I co-reviewed this manuscript with one of the reviewers who provided the listed reports. This is part of the Nature Communications initiative to facilitate training in peer review and to provide appropriate recognition for Early Career Researchers who co-review manuscripts.

Reviewer #4

(Remarks to the Author)
The authors have addressed my previous comments by noting that glycan-specific functional experiments, particularly at sites such as N88 and N611, are beyond the scope of the present study and are deferred to future work. While the data support the observed global Env tilting behaviour, the proposed glycan-mediated mechanisms remain, as previously noted, largely based on simulations. Overall, the authors' acknowledgement of the limitations of MD simulations and the need for future experimental validation helps to place the mechanistic conclusions in an appropriate context.

Open Access This Peer Review File is licensed under a Creative Commons Attribution 4.0 International License, which permits use, sharing, adaptation, distribution and reproduction in any medium or format, as long as you give appropriate credit to the original author(s) and the source, provide a link to the Creative Commons license, and indicate if changes were made.
In cases where reviewers are anonymous, credit should be given to 'Anonymous Referee' and the source.
The images or other third party material in this Peer Review File are included in the article's Creative Commons license, unless indicated otherwise in a credit line to the material. If material is not included in the article's Creative Commons license and your intended use is not permitted by statutory regulation or exceeds the permitted use, you will need to obtain permission directly from the copyright holder.
To view a copy of this license, visit https://creativecommons.org/licenses/by/4.0/

Point by point Response to Reviewers

Reviewer 1

- *Shehata et al. construct and simulate a full-length, fully glycosylated model of the HIV-1 Env trimer in the context of membrane. In specific, they construct an Env trimer with modeled membrane-interactive region, add glycosylation at 69 sites (23 per protomer), add cysteine palmitoylation, and embed the model in an all-atom membrane bilayer. They then perform microsecond-long MD simulations (with 5 independent replicas) quantifying motions, especially trimer tilt angle, as they find substantial tilts of ~30 degrees in most cases. They use cryo-ET on eVLPs to experimentally visualize Env tilting. They also quantify glycan-membrane contacts, finding glycans 88 and 611 to dominate. Using kernel density estimates, they analyze the distribution of MPER and MPER-TMD hinge angles. Lastly, they assess the glycan shield effectiveness in occluding underlying protein residues, as well as the ability of Env tilting to expose the MPER.*

  Overall, a very nice paper demonstrating by molecular dynamics and by electron microscope that tilting of the HIV-1 Env trimer in membranes is more pronounced than previously anticipated.

Response: We thank the reviewer for the positive feedback.

- *One substantial issue: the initial model. On line 124, the manuscript states that the model includes the ectodomain (residues 631-662) from PDB ID: 5T3A (reference 23). Likely there is a typo, and this should be (residues 31-662).*

Response: We acknowledge a typographical error in the manuscript regarding the residue numbering: the ectodomain range should indeed be residues 31–662, not 631–662. This has now been corrected in the revised version of our manuscript. However, we would like to clarify that the PDB ID is correctly reported as 5T3Z, which is a high-resolution crystal structure of the BG505 SOSIP.664 trimer (Gristick et al., 2016; https://doi.org/10.1038/nsmb.3291). It appears the reviewer may have inadvertently cited "5T3A," which is not related to HIV Env.

- *The more critical issue is that the PDB ID selected, 5T3A has a mistake in the register of its final helix (off by 1 residue). This mistake was first incorporated into the 4TVP structure (Pancera et al. 2014 – the first Env trimer structure with gp41 coordinates – and propagated*

through several other structures until it was corrected in the Stewart-Jones et al. structures that were published in Cell 2016. The critical point is that the alignment of sequence and structure are incorrect in this critical region. The authors should either correct the model and repeat the simulations or - if that is not possible - then the authors should at least comment on how the incorrect model impacts their results, especially the critical MPER and MPER-TMD hinge angles as well as the tilting overall.

Response: We appreciate the reviewer's focus on this region of the model, as the junction between the ectodomain and membrane-proximal segments is one of the most technically complex and biologically important components of our full-length Env model. Achieving a coherent, physically realistic connection between these domains is far from a trivial task and requires careful modeling to avoid register mismatches, geometric distortions, and unphysical conformations. One of the central challenges in modeling full-length Env is that the final helix of the ectodomain and the initial helix of the TMD have never been experimentally resolved together in the context of the full-length trimer. These regions have only been characterized separately in distinct experimental constructs: SOSIP-based ectodomains truncated before the membrane, and MPER-TMD segments solved independently in membrane mimetics. As a result, the atomic coordinates available from these fragments are not directly compatible for seamless assembly. As the reviewer rightly points out, attempting to directly merge these coordinates would result in discontinuities, steric clashes, and artificial kinks in the backbone—issues that are only exacerbated if register mismatches are already present in the underlying structural data.

Informed by this context, and after consultation with some of the authors of the original 5T3Z structure, we opted for a structure-guided integrative modeling approach that avoids inheriting these known artifacts. We would like to clarify that this procedure is already fully detailed in the *Full-length model assembly* paragraph in the Supplementary Information, which may help address the reviewer's concerns. Briefly, rather than depending only on the available raw PDB coordinates for this region, we generated the connecting HR2 helical segment (residue 637–668, Uniprot residue numbering) de novo using AlphaFold2, guided solely by the full-length clade A sequence. To maintain consistency and eliminate redundancy, we first removed the N-terminal residues from the MPER-TMD-CT model and the C-terminal residues from the ectodomain model, then we connected the two regions via the AlphaFold2-predicted helical segment. The resulting junction was further refined by local energy minimization in VMD. This approach allowed us to construct a seamless and biologically plausible bridge between the ectodomain and MPER-TMD-CT regions without inheriting any geometric artifacts or register mismatch from previously solved fragments. Superimposition of the MPER region in our model with the corresponding MPER region resolved in the crystal structure (PDB ID: 4G6F) revealed remarkable structural similarity, underscoring the high quality of our model (see below and in Fig. S10, which was already present in the initial submission as Fig. S7).

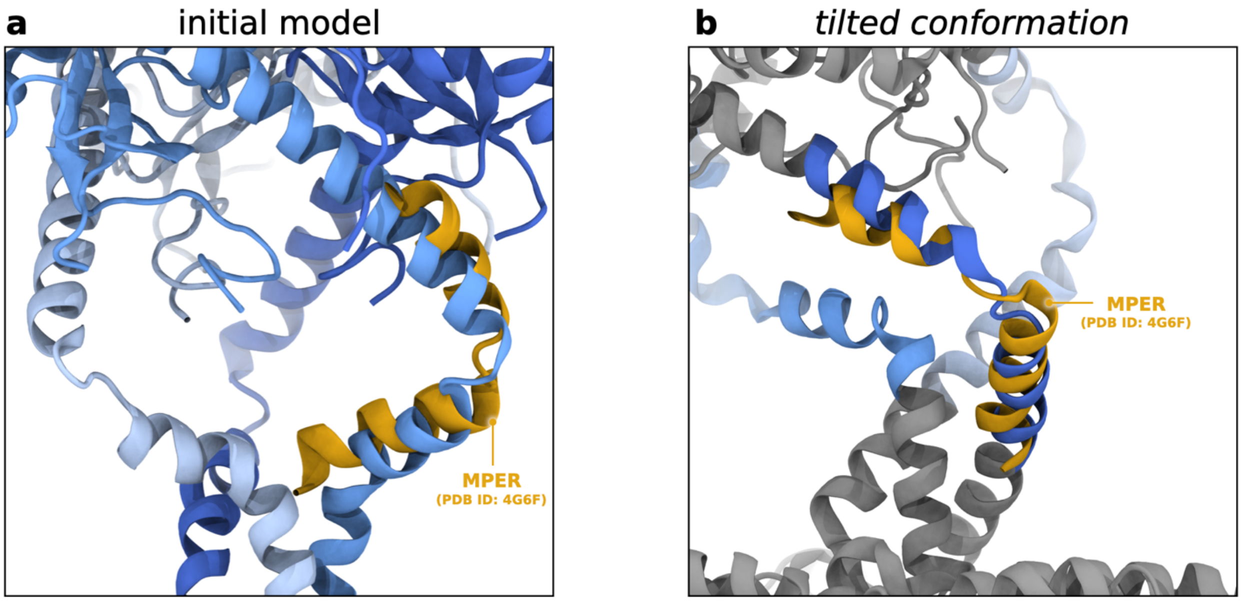

Supplementary Figure 10. Alignment of the MPER region from the crystal structure of 10E8 Fab in complex with the HIV-1 Env's MPER (yellow cartoons, PDB ID: 4G6F[14]) onto the HIV-1 Env's MPER as modeled in this study (blue cartoons). (**a**) Alignment onto the initial model. (**b**) Alignment onto a tilted conformation, resulting in a most exposed conformation of the MPER.

In summary, we respectfully clarify that our model was not constructed by directly simulating or concatenating raw PDB fragments. Instead, we applied an integrative modeling strategy that combined homology modeling and de novo structure prediction, followed by energy minimization, to ensure sequence continuity, structural coherence, and an energetically favorable conformation. This approach yielded a full-length Env model appropriate for molecular dynamics simulations.

Although we have taken steps to construct a coherent and biologically plausible model, we are aware that the absence of experimentally resolved coordinates for the full-length Env, particularly across the connecting segment between the ectodomain and the MPER-TMD, introduces an unavoidable degree of uncertainty. The reviewer's point is well taken, and in response, we have now explicitly acknowledged this limitation in the *Discussion* section of the revised version of the manuscript (see lines 447-454 on page 19). Nevertheless, our computational results showing Env tilting are corroborated by cryo-ET experiments and align well with previously proposed mechanisms of MPER-TMD plasticity, lending additional confidence to the validity of our model.

- *"Also, for some reason they've only added two of the four conserved gp41 glycans, leaving out the conserved N-linked glycans at residues 618 and 625. While Cao et al. (cited ref. 10) were unable to resolve these two glycans in viral Env, they did resolve in viral Env (from JR-FL and*

B41, so they are likely complex glycans observed elsewhere), and these two glycans should be added to any fully glycosylated Env trimer…………..."

Response: We appreciate the reviewer's attention to this detail, which gives us the opportunity to clarify the rationale behind our glycan modeling choices. Our study focuses specifically on modeling the native *clade A* BG505 HIV-1 Env trimer (UniProt accession number: Q2N0S6) as stated in our *Methods* section, and accordingly, we built our full-length, glycosylated model based on the BG505 SOSIP.664 trimer, a well-characterized clade A Env trimer. For structural modeling, we used the high-resolution crystal structure deposited as PDB 5T3Z, described by Gristick et al. (https://doi.org/10.1038/nsmb.3291) as a template. To approximate the native viral form, we reversed the stabilizing SOSIP modifications, restoring the wild-type sequence.

Glycosylation patterns across HIV-1 Env trimers vary significantly between strains and clades. To ensure an accurate representation of the glycosylation state, we based our model's glycoprofile on experimental data from *clade A* viral BG505 Env, rather than assuming site occupancy patterns from other clades. A key source was indeed Cao et al. (2018) (https://doi.org/10.1038/s41467-018-06121-4), who compared glycan occupancy across multiple Env clades and constructs. Importantly, in the BG505 viral Env, they found that glycosylation sites N618 and N625 were not occupied, as shown in their Figure 2b. This result directly informed our decision to exclude these sites from our model.

While gp41 typically includes five canonical N-linked glycosylation sites (N611, N616, N618, N625, and N637), their occupancy is known to vary by strain, construct, and expression system**.** The reviewer correctly notes that JR-FL and B41 Envs exhibit occupancy at N618 and N625; however, these are *clade B* strains, and their glycan profiles should not be generalized to *clade A*. Our model reflects the experimentally observed glycosylation of BG505, a *clade A* isolate, ensuring clade-specific and biologically relevant accuracy.

Several studies have independently documented the variable nature of gp41 glycosylation, particularly at sites N618 and N625, reinforcing that their occupancy cannot be assumed across constructs or clades. For example, Cao et al. (2017) (https://doi.org/10.1038/ncomms14954) analyzed soluble BG505 SOSIP.664 trimers and found that while N611 and N637 were consistently occupied**,** N618 and N625 were only partially glycosylated. Expanding on this, Behrens et al. (2016) (https://doi.org/10.1016/j.celrep.2016.02.058) reported that N625 glycan occupancy was markedly lower than other gp41 sites, with glycopeptides detected only at trace levels and insufficient for confident MS/MS verification. They also noted that N611 and N618 are likely positioned near the MPER, and that in the context of full-length Env, shielding effects from the MPER and the virion or cell membrane may further constrain glycan processing at these sites. This spatial arrangement may help explain why N618 is often under-occupied in native viral environment, likely due to steric hindrance from neighboring glycans and the membrane that limits efficient processing at this site. Furthermore, Depetris et al. (2012)

(https://doi.org/10.1074/jbc.M112.371898) observed that in cleaved SOSIP.681 trimers, only three of four canonical gp41 glycosites were consistently occupied**,** with partial glycosylation at N611 and N616 and variable presence at N625. Supporting this variability strains, Go et al. (2009) (https://doi.org/10.1021/pr9002728) showed that N625 was completely unutilized in a consensus clade C construct (C.CON), and inconsistently glycosylated in wild-type clade C Envs. Finally, Go et al. (2011) (https://doi.org/10.1128/jvi.05053-11) demonstrated that although N625 was fully occupied in one transmitted/founder *clade C* Env (C.1086), adjacent sites N611 and N616 remained only partially glycosylated, further emphasizing that even conserved PNGS motifs in gp41 exhibit variable occupancy depending on the viral background and construct design.

Taken together, these studies underscore that N618 and N625 are not consistently occupied across all HIV-1 strains, and most critically, they are largely absent in *clade A* viral BG505 Env glycoprofile**.** Including them in our model would introduce glycan features not supported by structural or biochemical evidence. We therefore respectfully maintain that the exclusion of N618 and N625 is scientifically sound, strain-specific, and experimentally validated. In the revised version of the manuscript, we have clarified this rationale. Specifically, we now explain in the *Methods* section that the PNGS glycosylated in our model reflect those with consistent experimental support for occupancy in the context of clade A BG505 Env (see lines 585-593 on page 24). Moreover, we removed the word *fully* from "fully glycosylated model" to avoid potential misinterpretation. We now refer to the model as "Full-length, glycosylated model of the HIV-1 Env trimer." Finally, we have incorporated all the relevant references discussed in this response.

- *Please cite Lee et al., 2024 Comput Struct Biotechnol J., as a prior study that used molecular dynamics to investigate a fully glycosylated HIV-1 Env trimer.*

Response: We thank the reviewer for pointing out this recently published study. We have incorporated a citation to *Lee et al., 2024 (Comput Struct Biotechnol J.)* in the *Introduction* of the revised manuscript (see lines 79-80 on page 4), acknowledging it as an important prior example of molecular dynamics applied to a fully glycosylated HIV-1 Env trimer.

- *Please change "Conformational Plasticity" in title and text to "Tilting in Membrane"; the major alteration in structure is the relative tilt of the Env trimer, not its altered folding/conformation. There are other studies such as the Lee et al. 2024, which address altered folding/conformation, so good to separate these studies and to clarify for readers that it is mainly the tilt that is altered, not the folding of the Env protomer.*

Response: We thank the reviewer for the suggestion to revise the term “conformational plasticity” both in the title and in the text. We agree it is important to distinguish our work from studies focused on protomer refolding or misassembly, such as Lee et al. (2024). In the *Introduction* of the revised version of the manuscript, where we now mention the contribution by Lee et al. (2024), we have remarked that the simulations performed therein were used to study large-scale conformational changes of HIV-1 Env. According to the reviewer’s comment, we have now changed the title to *N-linked glycans modulate tilting of HIV-1 Envelope glycoprotein.* We have also changed several instances throughout the main text where we referred to glycans as ‘modulators of conformational dynamics’ to ‘modulators of Env’s tilting dynamics’.

- *The authors should comment on the impact of the two missing gp41 glycans at 618/625 on their results.*

Response: We thank the reviewer for raising this point. As addressed in our previous response, we have now explained in the revised Methods section the rationale behind not including glycans at N618 and N625 in our model. This modeling decision is based on published glycoproteomics data indicating these sites are unoccupied in the BG505 clade A virion Env. In addition, in the *Methods* section of the revised manuscript (see lines 585-593 on page 24) we now explicitly comment on the variable nature of gp41 glycan occupancy across strains and constructs, which further supports the context-specific modeling of these sites.

- *The implications of tilt and MPER-based neutralization are fascinating; it was previously reported by Rantalainen et al., Cell Reports that MPER targeting antibodies tilt the Env trimer in relation to the bilayer; please cite this prior structural study carried out in lipid nanodiscs.*

Response: We appreciate the reviewer’s emphasis on the study by Rantalainen et al. (*Cell Reports*), which we fully recognize as a landmark contribution to understanding Env tilting and MPER-targeted neutralization in a membrane context. In fact, we already cited this work multiple times throughout the manuscript, as it forms a central part of the conceptual and structural framework underpinning our study. Our findings align closely with the model proposed by Rantalainen et al. for MPER-targeting neutralization in lipid bilayer assemblies, including the suggested orientations of HR1, HR2, MPER, and TMD as illustrated in Figure 6 of their study. Consistent with their observations, our simulations reveal that native Env spontaneously samples a range of functional states, including tilted conformations, even in the absence of antibody binding. This highlights the intrinsic conformational flexibility of the MPER-TMD region and supports the progressive binding model proposed by Rantalainen et al. We have further emphasized this agreement in the

Discussion section of the revised version of the manuscript to underscore the strong consistency between our results and their proposed mechanism (see lines 439-446 on page 19).

- I *like the attempt to assess the glycan shield's effectiveness. Similar studies have been carried out including by GLYCO (Lee et al., 2022 Bioinformatics) with epitope exposure calculated in Chuang et al., 2019 Structure. These prior investigations should be cited.*

Response: We thank the reviewer for this helpful suggestion. We agree that the studies by Lee et al. (2022, Bioinformatics) and Chuang et al. (2019, Structure) represent important prior efforts in quantitatively assessing glycan shield effectiveness and epitope exposure. We have now cited both studies in the *Discussion* section of the revised version of the manuscript (see lines 520-524 on page 22) and have referenced them in the context of our own analysis to appropriately acknowledge their contributions to this area of research.

- *References 62 and 85 are the same.*

Response: We thank the reviewer for catching this repetition, which we have corrected in the revised version of the manuscript.

- *The authors should also comment on the impact to the glycan shield of leaving off glycans 185e and 185h.*

Response: We sincerely appreciate the reviewer's comment regarding glycans 185e and 185h. This is indeed an important point, and we are grateful to the reviewer for bringing it to our attention, as it warrants clarification and discussion. These sites are located within the highly flexible and structurally unresolved V2 loop, and are particularly challenging to characterize in terms of glycosylation. Notably, both sites occur within the motif **N**RS**N**NS, which places them in extremely close proximity. This makes it particularly difficult to resolve them individually by protease digestion and mass spectrometry, potentially contributing to their ambiguous reporting in experimental datasets. In the mass spectrometry dataset we used to guide our model (Struwe et al., *Cell Rep.* 2018), the corresponding N190 and N190c sites were reported with relatively low occupancy in soluble BG505 constructs, but exhibited higher occupancy in the membrane-associated, virion-derived form. This variability is further supported by Cao et al., *Nat. Comms.* 2017, who found these sites to be only partially occupied in soluble BG505 trimers. Similarly, in Cao et al., *Nat. Comms.* 2018, 185e and 185h were also found to be partially unoccupied in virion-derived BG505 Env, indicating that incomplete glycosylation at these positions is not limited to recombinant constructs, but may be possible in a virion-derived context. In addition, Derking et

al. *Cell Rep.* 2021 (https://doi.org/10.1016/j.celrep.2021.108933) showed that occupancy at these sites could only be increased by converting such sequons to NxT, suggesting that inefficient glycosylation may be influenced by sequence context (NxS instead of NxT) as well as expression system. Given the lack of consistent and robust characterization across systems, as well as the inherent uncertainty associated with de novo modeling of the V2 loop, we made a pragmatic decision to omit glycans 185e (190) and 185h (190c) from our base model. We have now included a statement in the *Methods* section to clarify this modeling choice (see lines 585-593 on page 24).

However, to further address the reviewer's comment we have generated and simulated an updated Env model where glycans 185e and 185h were incorporated as FA2/FA3 according to Derking et al. *Cell Rep.* 2021. We believe that this supplemental Env model, which captures a more heavily shielded V2 apex, may be of great use to the community. Modeling details are presented in the Supplementary Information. Results from this new set of five microsecond-long replicate simulations are instead presented in the main text: V2 shielding is reduced, with partial effects extending to the V3 crown, while the V3 base remains accessible. These findings are discussed in the *Results* section (see lines 365-368 on page 16), further elaborated in the *Discussion* (see lines 538-544 on page 22), and illustrated in Supplementary Figure 8.

- *There has been some debate on "state 1" identified by smFRET by Mothes and colleagues; the authors should comment on whether or not a tilted Env conformation might account for "state 1".*

Response: As pointed out by the reviewer, the structural interpretation of smFRET-defined "state 1," as described in Lu et al., Cell 2019 and earlier work by the Mothes lab, remains an area of active discussion. State 1 is generally considered the closed, ground-state conformation of Env on the virion surface, but its precise structural correlate remains unknown. As Lu et al. emphasize, state 1 is the predominant conformation present on native Env, and is also preferentially recognized by many broadly neutralizing antibodies (bnAbs). Despite its functional and immunological importance, state 1 has yet to be structurally resolved. smFRET studies reveal that native, membrane-anchored BG505 Env predominantly occupies state 1, while soluble SOSIP.664 constructs, despite sampling all three smFRET states, are biased toward state 2, a more open intermediate. Lu et al. further showed that deletion of the cytoplasmic tail had little effect on the conformational landscape, whereas binding of PGT151 shifted Env from a state-1-like to a state-2-like conformation. They also demonstrated that SOSIP.664 trimers fail to re-equilibrate toward state 1 even in the presence of allosteric inhibitors such as BMS-378806 or its more potent analog BMS-626529, unless used in large excess. In contrast, native Env readily shifts into or is stabilized in state 1 in response to multiple bnAbs (e.g., PG9, 3BNC117, 10-1074). Importantly, Lu et al. conclude that existing high-resolution structures of Env most closely resemble state 2, which is only transiently populated on the virion. State 1 remains structurally uncharacterized, yet is

biologically critical and likely underrepresented in available structural models. These findings strongly motivate efforts to explore state 1 through alternative approaches, including membrane-embedded models and dynamic simulations.

Our simulations of membrane-embedded BG505 Env capture spontaneous and persistent tilting of the ectodomain relative to the membrane. Although tilting does not directly affect the spatial relationship between the regions typically labeled in smFRET experiments, it nonetheless reflects a relevant dynamic mode of the native Env trimer. While we recognize that such flexibility could, in principle, contribute to the broader conformational landscape captured by smFRET, extreme caution is warranted, as definitive conclusions would require targeted experimental validation beyond the scope of the present work. Accordingly, we have not added any discussion of this point in the revised manuscript, as this would be too speculative to be commented on and beyond the scope of the present work.

Reviewer 2

- The paper by Shehata and colleagues reports a very interesting and timely study of the multiplicity of effects that glycans have on protein functions, dynamics, and immune-recognition mechanisms. Specifically, the authors study the Envelope protein of HIV in its fully glycosylated and membrane embedded form at an atomistic detail. One very interesting aspect of this paper is that, while the systems and simulations are highly complex, the authors make an effort to reconnect their results o experimental information. In this context, the inclusion of CryoET data is very interesting, and shows how simulations and experiments should converge to provide biologically relevant information. The system is challenging and the simulations are carried out in a technically sound way. I would move some of the details on Glycosylation modeling to the main text instead of leaving that in the SI. Overall, the paper is very well conducted and well written. This group has already given significant contributions in this area and this is certainly another great example. The interesting part of the paper is that they show the multiple personalities of sugars, not only as shield but also as conformational modulators and interactors with the membrane. The visualization of the Ab-binding epitope is interesting and shows that these approaches could actually be used in structural vaccinology.

Response: We thank the reviewer for the positive feedback.

- I suggest that the authors make an additional effort to make their discussion and overall concepts more general: They could expand the discussion to other cases where glycans are shown to modulate the dynamics also of non-viral proteins, with an impact on their ability to bind drugs, for instance.

Response: We appreciate the reviewer's suggestion and have expanded the *Discussion* section of the revised manuscript to include non-viral examples where N-glycosylation modulates protein dynamics with functional consequences (see lines 488-494 on page 21). For instance, in GRP94, site-specific glycosylation reshapes the conformational ensemble and influences ligand binding (Castelli et al. *Structure* 2023, Magni et al. *Chemistry – A European Journal* 2024), or in IgG1, where glycosylation modulates local structural stability and receptor affinity (Subedi et al. *Structure* 2015). These findings highlight the broader principle that glycans regulate structural plasticity and molecular recognition across diverse protein systems, extending the relevance of our observations beyond HIV-1 Env.

- In terms of the epitope discovery/ab-binding part, the authors could also discuss some of the limitations of their approach (MD simulations may be demanding in terms of time and computing time requests) and whether it would be amenable to integration with other methods for epitope prediction.

Response: We thank the reviewer for this thoughtful suggestion. In response, we have expanded the discussion to highlight how complementary computational strategies, such as energy decomposition frameworks and emerging large language model-based predictors, could be integrated to enhance the efficiency and scalability of epitope discovery. This addition has been incorporated into the revised *Discussion* (see lines 549-551 on page 23).

Reviewer 4:

- *The technical execution is strong. However, the distinct contribution of the study is substantially reduced by prior work, particularly the study by Prasad et al. (Cell, 2022).*

Response: We thank the reviewer for acknowledging the technical strength of our study. We also appreciate the reference to the excellent work by Prasad et al. (Cell, 2022), which we have cited multiple times throughout the manuscript and regard as an important foundation in the field. That study made key contributions to understanding Env variability in situ through cryo-electron tomography (cryo-ET), particularly regarding positioning on the Gag lattice, stalk flexibility, and glycan heterogeneity.

That said, our study is distinct both in scope and in resolution. While Prasad et al. elegantly revealed structural variation at the nanometer scale, our approach focuses on the atomistic dynamics of a full-length, fully glycosylated Env trimer embedded in a lipid bilayer, something not accessible through cryo-ET alone. Specifically, our simulations capture microsecond-timescale motions of the MPER-TMD region and reveal key glycan-membrane contacts that shape the dynamic behavior of the glycan shield. By modeling Env in explicit membrane and solvent, we uncover how these membrane-proximal dynamics influence epitope accessibility, particularly in the MPER, a target of broadly neutralizing antibodies. We also characterize the structural flexibility and energetic landscape that govern transitions between shielded and exposed states. These insights provide mechanistic understanding that complements cryo-ET observations.

We agree that tilt is observed in both studies, but our work captures the molecular determinants of tilt, including hinge flexibility and glycan interactions, at a resolution that enables testable mechanistic hypotheses and downstream modeling. Notably, the fact that our simulations reproduce the tilting behavior observed by cryo-ET underscores both the predictive strength and structural fidelity of our full-length Env model. In that sense, we believe the two papers are not overlapping but complementary, together offering a multiscale view of Env plasticity and accessibility.

This distinction is explicitly highlighted in the *Discussion* section (see lines 435-437 on page 19, and lines 516-518 on page 21) to help readers appreciate how the two approaches address similar biological questions from fundamentally different angles, with our MD-based framework providing mechanistic insight and dynamic resolution that reinforces and extends experimental observations.

- *"p.1 While cryo-ET was performed, no experimental perturbations such as analyses of glycan deletion mutants were carried out to directly assess the individual or combined roles of N88 and N611 in Env tilting. Without such targeted controls, the proposed functional relevance of these glycans remains a plausible but unverified interpretation. On the other hand, although N611A and N88A mutants were reportedly generated (according to the supplementary methods), their subtomograms were pooled with wild-type data solely to increase particle numbers, without independent analysis. This approach weakens the central claim that N88 and N611 modulate Env tilting, as it lacks direct experimental validation. If these glycans do affect tilting, mixing mutant and WT particles could bias the structural averages and tilt distributions, thereby compromising the reliability of the conclusions."*

Response: We thank the reviewer for this thoughtful and constructive comment. The central finding of our study is that the N-linked glycans at N88 and N611 facilitate Env tilting relative to the viral membrane. Our data indicate that these glycans act early in the tilting process by transiently interacting with the membrane, thereby steering the protein towards more tilted orientations. We agree that direct experimental perturbations, such as analyzing glycan-deletion mutants, would provide valuable additional validation. Cryo-ET, however, captures an ensemble of Env conformations at the moment of vitrification, reflecting the distribution of states from upright to tilted. Both WT and glycan-deletion mutants are therefore expected to show tilting in cryo-ET, but subtle differences in tilting propensity are difficult to resolve with this approach. By contrast, our simulations probe the nanosecond-to-microsecond regime where glycan-mediated effects are initiated, allowing us to quantify how the presence of N88 and N611 increases the likelihood of tilting transitions. To support this observation, we have now included five additional microsecond-scale MD simulations of the double N88A/N611A mutant where the respective N-linked glycans are effectively deleted (modeling details are described in the Supplementary Information). These new simulations reveal that in the absence of both glycans, Env remains predominantly upright with a markedly reduced propensity to tilt. These results, now presented and discussed in the revised version of the manuscript (see lines 188-194 on page 9, lines 272-277 on page 13, and lines 471-479 on page 20) provide further direct mechanistic support for our hypothesis that N88 and N611 act cooperatively to increase propensity of Env tilting transitions.

We also appreciate the reviewer's concern about pooling mutant and WT subtomograms. In the original submission, a small subset of mutant particles was combined with WT (base system) data to increase particle numbers, which could indeed slightly bias tilt distributions. In the revised manuscript, we have collected additional cryo-ET data for WT (base system), enabling fully independent WT (base system) analyses without pooling. All cryo-ET results now reflect WT-only datasets (lines 154-177 on page 8 in the main text, Figure 2 in the main text, sections 1.3, 1.4 in the Supplementary Information, and Figures S2-S3), and the conclusions remain unchanged: Env exhibits a broad tilt-angle distribution consistent with the dynamic tilting captured in MD simulations.

Finally, we acknowledge that functional assays of glycan-deletion constructs would further strengthen the conclusions. However, such experiments fall beyond the capacity of the present study but would be valuable to include in a future follow-up study. We now explicitly highlight this limitation in the *Discussion* and outline it as an important avenue for future work (see lines 471-479 on page 20).

- p.2 GP41 CT of Env interacts with the MA lattice beneath the membrane, which likely affects the Env dynamics and tilting. The simulation system should include the MA lattice to be closer to the native condition.

Response: We agree with the reviewer that interactions between the gp41 cytoplasmic tail (CT) and the underlying MA lattice are an important aspect of the native virion environment and could influence Env dynamics and tilting. However, explicitly including the MA lattice is beyond the scope of the present study. Our current focus was on resolving Env's conformational variability in a native-like membrane context at atomistic detail, which already represents a substantial system size and computational challenge.

We acknowledge this as an intrinsic limitation of the present work, along with the lack of full virion curvature. Both aspects could be addressed in future efforts, for example through cell-scale or whole-virion simulations, which may allow for a more complete treatment of Env–MA interactions. We have added a statement in the *Discussion* section of the revised manuscript to clarify this point and to outline possible future directions (see lines 454-462 on page 19).

- p.3 Oversimplified membrane model in MD simulations. The MD simulations model the membrane as flat and tensionless, whereas authentic HIV-1 membranes are expected to be highly curved and heterogeneous. Such membrane properties could significantly impact Env tilting dynamics. Although the manuscript briefly mentions curvature, it underestimates its potential effects. A more thorough discussion of the limitations of the current membrane model, and an explanation of how membrane curvature and tension might alter the results, would be highly valuable.

Response: We thank the reviewer for raising this important point. We fully agree that the authentic HIV-1 envelope is highly curved and heterogeneous, and that these membrane properties may influence Env dynamics in ways that are not fully captured by our current simulations. In our study, we modeled Env in an asymmetric but flat and tensionless bilayer designed to reproduce the lipid composition of the viral budding site. This setup provides an experimentally grounded environment for investigating membrane-proximal interactions, but it does not recapitulate the full

complexity of the viral envelope, including local curvature, tension, and the presence of the underlying MA lattice.

In the revised manuscript, we have expanded our *Discussion* to address this important point in greater detail (see lines 454-462 on page 19). Specifically, we note that curvature could increase the conformational space available for Env motion, potentially allowing larger tilt angles than those sampled in a flat bilayer. Likewise, local membrane tension and heterogeneity might modulate the energetic landscape of tilting transitions by altering the depth and frequency of glycan–membrane contacts. These considerations may help explain why our cryo-ET analyses of Env in virus-like particles revealed a somewhat broader tilt distribution than observed in simulations.

Importantly, the experimental and computational approaches complement one another: cryo-ET captures the broad distribution of Env orientations in the native viral membrane, while MD simulations provide atomistic insights into the molecular mechanism underlying these motions. Together, they demonstrate that Env tilting is an intrinsic property facilitated by N88 and N611 glycans.

- Minor points:
  p.3. The manuscript repeats background information (e.g., glycan shielding, immune evasion) in several sections. For example, statements such as, "Understanding the strategies that viruses employ to evade the host immune response is crucial for designing effective vaccines. Among these, the glycan shield stands out as one of the most effective mechanisms utilized by class I viral fusion glycoproteins, including HIV-1 Env, to thwart the humoral response. Approximately half of the molecular weight of Env is derived from N-glycans, which are strategically distributed across the ectodomain," appear multiple times or convey overlapping content. The authors should carefully revise the manuscript to eliminate such redundancies and improve the overall clarity, and readability of the text.

Response: We thank the reviewer for this helpful comment. We have streamlined the manuscript to minimize redundancies as much as possible, especially in the glycan shield section, ensuring that background information is presented only where most appropriate. In particular, general background material has largely been consolidated into the *Introduction*, while the *Results* now focus more on presenting our findings. Finally, we have introduced a separate *Discussion* section, where we place our results in the context of prior literature, highlight the limitations of our study, and outline potential implications for immunogen design and future work. We believe this restructuring has improved clarity and readability.

- p.4. Include a clear discussion of limitations and outline what experimental work would be necessary to validate the proposed mechanism.

Response: We thank the reviewer for this suggestion. In the revised manuscript we have added an extended *Discussion* section where we now explicitly note the limitations of our simulations and outline potential experimental strategies to validate the proposed mechanism (see lines 454-462 on page 19, lines 471-479 on page 20).

- p.5. The authors state that “This discovery builds on prior evidence highlighting the critical role of N88 and N611 glycans in shaping Env’s antigenicity.” However, to be considered a discovery, such a claim requires direct experimental validation.

Response: The reviewer’s point is well taken. Although our cryo-ET data independently corroborate the presence of Env tilting—a central component of the mechanism we propose—we acknowledge that our primary findings regarding the roles of the N88 and N611 glycans are based on all-atom simulations. Accordingly, in the revised manuscript, we have softened our wording to “*This finding adds to prior evidence highlighting the critical role of N88 and N611 glycans in shaping Env’s antigenicity.”*

Point by point Response to Reviewers

Reviewer 1

- *In their manuscript entitled, N-Glycans Modulate Tilting of HIV-1 1 Envelope Glycoprotein, Shehata et al. analyze a fully glycosylated full-length model of the HIV-1 Env trimer and find substantial tilting.*
- *In my prior review of the manuscript, I noted that there might be inaccuracies in the model related to the fact that many historical Env trimers did not define accurately the final C-terminal residues of the 664 model, such as PDB 5T3Z, which served as an initial template for their molecular dynamics. The authors have done a decent job providing caveats to their model. However, the discussion of the model construct and analyze is unclear: do the starting coordinates have the incorrect C-terminus of 4TVP and many other models including PDB 5T3Z, or the corrected C-terminus of the latter structures (e.g., from the Stewart-Jones Cell 2016 manuscript)? I accept that modeling of trimers is still being experimentally resolved and am okay with the authors' caveats and model; however, they should make clear to readers the register of the C-terminal region around reside 664 and to which PDB it most closely aligns. (Likewise, in their Supplementary Fig. 10, the authors should add a few residues numbers on aligned peptide and model; it looks like there is decent overall 2nd structural similarity, but authors need to clarify if sequence register is preserved, which residue numbering should help to address.)*

Response: We appreciate the reviewer's continued attention to the structural accuracy of our model and the opportunity to clarify this point further. Based on our analysis, we remain confident that a register error in this region is not present.

The ectodomain's HR2 helix (residues 637–668), which includes part of the MPER, was modeled *de novo*, meaning that its atomic configuration was generated directly from the amino-acid sequence rather than adapted from any prior structural coordinates. In *de novo* modeling, residue placement, secondary-structure formation, and backbone geometry are dictated by sequence-derived physical constraints and energetic optimization, rather than by templated alignment to an experimental structure. As a result, the HR2 segment in our construct does not inherit any register mismatches, geometric distortions, or coordinate biases from prior models. Instead, it provides a physically coherent and intentionally constructed bridge between the ectodomain and MPER–TMD–CT regions, addressing a longstanding gap in full-length Env modeling.

As noted previously, superimposition of the MPER region in our model with the corresponding MPER peptide resolved in the crystal structure (PDB ID: 4G6F) shows strong structural agreement (Fig. S10), supporting the accuracy and experimental consistency of our approach. In addition, structural alignment with PDB ID: 5FYL—the same structure from the Stewart-Jones Cell 2016 manuscript strongly endorsed by the Reviewer—confirms that no caveats or register shifts are

present. We include below an image of this alignment, with residue 664 highlighted in both structures (5FYL in orange; our model in translucent cyan).

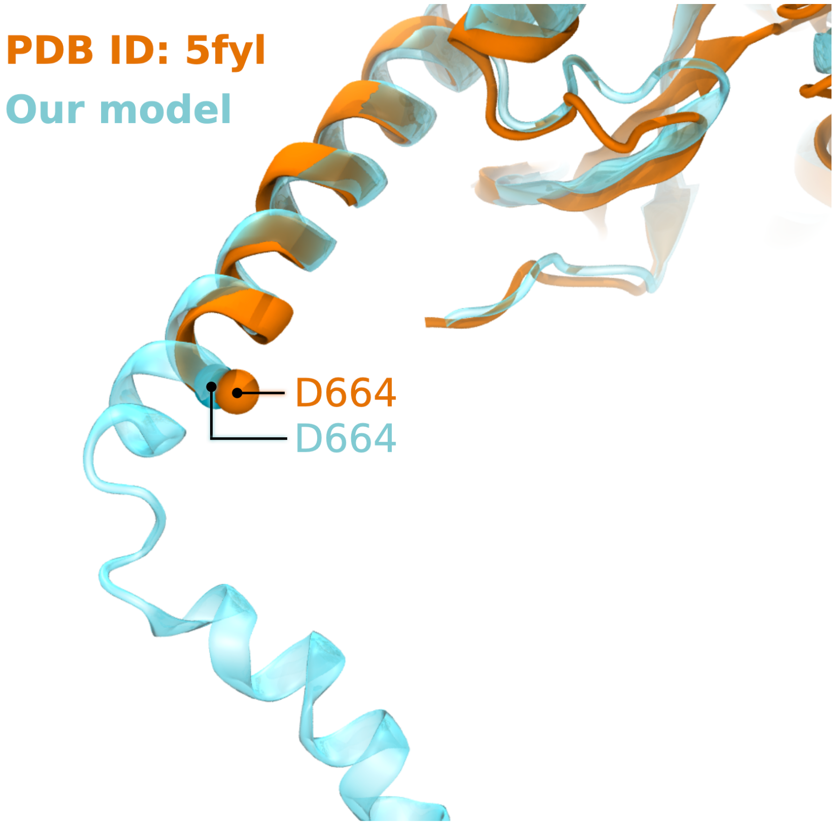

- *In terms of the glycans on the model, the authors state in their rebuttal that "A key source was indeed Cao et al. (2018) (https://doi.org/10.1038/s41467-018-06121-4), who compared glycan occupancy across multiple Env clades and constructs. Importantly, in the BG505 viral Env, they found that glycosylation sites N618 and N625 were not occupied, as shown in their Figure 2b."*
- *But this is not correct.*
- *In specific, when looking at Cao et al. Figure 2b, four BG505 constructs are shown, three membrane associated (BG505 dCT N332, BG505 dCT T332, and BG505 viral Env) and one soluble (BG505 SOSIP.664). The two dCT are membrane associated, but missing the intracellular region. Notable, glycans N618 and N625 are fully present on both of the dCT versions, and for the BG505 viral Env, no bar graph is provided. In the legend to Fig. 2b, Cao eta l. state: "… no bar graph indicates the site was not observed in LC/MS." This means that there is no data on the site, but not that they are unoccupied. For example, on the soluble BG505 construct, there is substantial non-occupancy shown in the LC/MS data (that is, non-occupancy and no data are two very different results).*

- *Importantly, this means that contrary to what the Shehata et al. state in their rebuttal, Cao et al. provide evidence that glycans 618 and 625 are glycosylated on membrane-bound Env trimer (2 out of 3 samples) and for the 3rd sample, they did not provide any data on whether these two glycans were occupied or not.*

Response:

We appreciate the reviewer's thoughtful comments regarding the interpretation of the Cao et al. (2018) dataset and the glycan assignments at sites N618 and N625. We have carefully re-examined the relevant data in light of this feedback and would like to clarify why our decision not to include these glycans in the base model remains scientifically well supported.

As the reviewer notes, in Figure 2b of Cao et al. (2018), the authors state that "sites with numbers and no bar graph indicate the site was not observed in LC/MS." For the BG505 full-length, virion-associated Env construct—the form directly relevant to our study—both N618 and N625 are labeled but lack corresponding bars. This indicates that peptides containing these sequons were not detected in the LC/MS dataset, and thus glycan occupancy at these positions could not be determined. In the absence of direct experimental evidence supporting glycosylation at N618 and N625 in the native, full-length Env, we did not include these glycans in our base model.

In contrast, in the same paper, Cao et al. reported glycan occupancy at these sites only in BG505-ΔCT constructs, which lack the cytoplasmic tail. Removal of the CT substantially alters Env's membrane association, intracellular trafficking, and glycan maturation in the Golgi, leading to glycosylation patterns that differ from those of the intact, virion-associated trimer modeled in our work. For this reason, ΔCT constructs are not representative of our system and do not provide an appropriate experimental basis for including glycans at these positions.

- *The authors do highlight a number of other references with soluble BG505 Env trimer. However, a major finding of the Cao 2018 paper is that the primary difference between soluble and membrane-bound trimers relates to the occupancy of gp41 glycans, with the supposition that in the soluble context, translation is different, and this alters gp41 glycosylation. Thus, these other references all cite soluble BG505 constructs, which are not relevant to the discussion of gp41 in the membrane-bound context.*

  Bottom line: Cao et al. were able to see glycans N618 and N625 on virion-associated trimers from other clades, as well as on two membrane-associated samples from BG505 though not a third, for which Cao et al. provide no data.

Response:

We appreciate the reviewer's careful consideration of the glycosylation evidence and the citations to both the BG505-ΔCT constructs and other clades analyzed in Cao et al. (2018). To clarify our interpretation, we would like to explain how these datasets relate to the specific system modeled in our study.

The reviewer notes that ΔCT constructs may support glycosylation at N618 and N625. At the same time, they correctly point out that soluble trimers are not appropriate for assessing gp41 glycosylation because they lack membrane-proximal features that strongly influence glycan processing. We fully agree with this latter point. Importantly, the same caveat applies, though in a different way, to the ΔCT constructs: although they remain membrane-associated, truncation of the cytoplasmic tail substantially alters Env membrane embedding, intracellular trafficking, and glycan maturation within the Golgi. As a result, ΔCT glycosylation patterns cannot be assumed to reflect those of the native, full-length Env.

Similarly, while Cao et al. (2018) provide valuable information across multiple viral clades, these data are not representative of the BG505 clade A Env examined in our work. Given the well-established clade-specific differences in glycosylation and Env architecture, these datasets cannot be directly extrapolated to our system.

Taken together, both the ΔCT constructs and constructs from other clades differ meaningfully from the full-length BG505 clade A Env that forms the basis of our model. Therefore, evidence of N618 and N625 occupancy in those systems does not provide a reliable experimental basis for modifying our modeling approach.

- *Shehata et al. really should including glycans N618 and N625 on their model.*
- *The problem is that Shehata et al. highlight as a major point of their analysis two other glycans, N88 and N611, as playing a critical role in modulating Env tilting. Similar to glycans N88 and N611, glycans N618 and N625 are located on the membrane-proximal region of Env trimer and would therefore be expected to influence membrane interactions/tilting.*
- *I'm okay with some inaccuracies in the model (e.g. 664 register), but if Shehata et al. are highlighting the impact of two glycans – they really need to make sure that they have the glycosylation for the 5 gp41 glycans on the membrane-proximal region correct, if they want to make conclusions about the impact of glycosylation on tilting.*

Response: We appreciate the opportunity to clarify our modeling decisions. Our full-length, glycosylated HIV-1 Env model embedded in a native-like membrane is constructed using the most comprehensive structural and analytic data currently available. Every aspect of the modeling, including residue numbering, full-length assembly, sequence alignment, glycan site assignment, and glycan composition, is described in detail in the Supplementary Information, ensuring complete transparency and reproducibility. Notably, we will also publish the full structural model so that others can build on it, varying glycosylation patterns, composition, or any number of seemingly infinite combinations. This process replicates what we did with the SARS-CoV-2 spike protein: our initial model (Casalino et al, ACS Central Science, 2020) has over 1000 citations to date, and many groups around the world (both academic and industrial) have used it as a basis for further exploration and refinement. We anticipate that the same will be the case with our work here.

In this work, we focused on what we found to be the most physiologically relevant configuration—our WT base system—complemented by the N88A/N611A mutant simulations added during the first round of review. These additional simulations provide strong mechanistic support for our conclusions regarding the structural and functional roles of the conserved N88 and N611 glycans in modulating Env tilting within the membrane. In this respect, we again thank Reviewer 1 for suggesting a revision of the title during the first review round, which we implemented to better reflect the outcomes of our study. Finally, a third system—the WT+N185e/h glycoform—was also built as per the reviewer's round 1 request to investigate the shielding role of the apex glycans. Collectively, this is a substantial set of models, simulations, experiments, and resources which can be further manipulated by others after publication.

Reviewer 2

- *This is a very strong paper with a technically elegant approach encompassing cryoEM and comp biology. I was already very supportive of it in the first submission. Now I see that the authors have run further analyses and went to great lengths to improve their paper in response to the other reviewers. I am strongly in favor of the publication of this paper*

Response: We sincerely thank the reviewer for their positive assessment of our work after revisions. We appreciate their strong endorsement and are pleased that the reviewer finds the study technically sound and ready for publication.

Reviewer 4

- *The authors have submitted a carefully revised manuscript that addresses some of the points raised in the previous review. They clearly explain how their work differs from Prasad et al. (Cell, 2022), include five new microsecond-scale MD simulations of the N88A/N611A mutant, and re-analyze the cryo-ET data using wild-type-only particles. These additions strengthen the mechanistic interpretation of the study.*

Response: We thank the reviewer for their thoughtful evaluation and for recognizing the substantial revisions made to the manuscript. We appreciate their acknowledgment of the clearer distinction from Prasad et al. (Cell, 2022), the addition of the five new microsecond-scale N88A/N611A simulations, and the re-analysis of the cryo-ET data using wild-type-only particles. We are glad that these additions strengthen the mechanistic interpretation and support our conclusions. We also thank the reviewer for noting the expanded discussion of limitations related to the absence of the MA lattice and native membrane curvature, and we appreciate their recognition that these points are now clearly articulated.

- *The limitations related to the absence of the MA lattice and membrane curvature are now acknowledged and discussed in greater detail. However, the experimental validation of the glycan effects remains lacking, which is a significant gap given the central focus of the manuscript. While the manuscript is technically sound, the study relies entirely on simulations without experimental validation. As such, the central claim remains hypothetical. Although the additional simulations strengthen the hypothesis to some extent, without experimental support, the impact of the findings remains limited.*

Response: We thank the reviewer for raising this important point regarding experimental validation and for the opportunity to clarify how experimental data informs and supports our study. Our work does include direct experimental validation through cryo-ET, which independently corroborates the core dynamic features observed in our simulations. Both MD and cryo-ET independently show that Env samples a broad range of orientations relative to the membrane, with closely matching tilt angle distributions. This behavior is also consistent with previous structural studies reporting Env tilting in both liganded and unliganded states. This agreement confirms that our model captures physiologically relevant Env tilting and that the overall dynamic landscape captured by the simulations is well aligned with the behavior observed experimentally in situ.

We agree with the reviewer that additional functional experiments—particularly those probing the specific roles of individual glycans—would further deepen understanding in this area. As Reviewer 4 noted in the first round, such studies represent a substantial and independent line of investigation. Further, they fall outside the scope of the present work, which is centered on constructing and experimentally contextualizing the first full-length atomic model of HIV-1 Env in its native membrane environment, and analyzing its dynamics to understand the role of glycans in its mechanism of recognition of host and immune evasion. We hope our provided model and manuscript will enable and motivate future experimental and computational studies addressing the glycan-specific questions raised, and much more.

Point by point Response to Reviewers

Reviewer 1

- *In "N-Glycans Modulate Tilting of HIV-1 Envelope Glycoprotein", Shehata et al. describe a microsecond-long, all-atom molecular dynamics simulations of a full-length, glycosylated Env glycoprotein embedded in a biologically relevant lipid bilayer. They find substantial tilting, an interesting result – and that this tilting depends on two membrane-proximal N-linked glycans at residues N88 and N611.*

  However, of the five conserved glycans on gp41, Shehata et al. removed two, at sites N618 and N625. These glycans tend to be underglycosylated on soluble HIV-1 Env, but on virion associated HIV-1 Env, their glycosylation was unclear, as Cao et al. 2018 were unable to obtain data on them – although Cao et al. did see that N618 and N625 were present in all other clades of HIV-1 that they examined.

  I'm okay with publishing the current paper in which N618 and N625 are missing, but the authors need to make clear to the readers that these two glycans were removed from the molecular dynamics – and these glycans are known to be present on all other clades. Bottom line: it is critical for the authors need to make clear to readers that these gp41 glycans (N618 and N625 - which are present in all other non-A clades) were missing in the molecular dynamics.

  Currently, the only place in the manuscript that this is noted is buried in the methods (lines 585-589), which will not be seen by most readers.

  It is critical for the authors to note in the first paragraph of the results that the simulation is missing two of the five gp41 glycans – that these glycans, at N618 and N625, are present in all other non-clade A strains of HIV-1 – and thus these results may be specific to clade A BG505, missing these two conserved glycans.

  Also, in the discussion, the authors should highlight for readers the absence of these two glycans, and the likely impact that their presence might have on the tilting that they observed.

Response: We thank the reviewer for their careful reading of our manuscript and for highlighting the importance of gp41 glycosylation at positions N618 and N625. We fully agree that the presence or absence of these glycans should be more clearly communicated to readers in the manuscript.

As suggested, we have now explicitly stated in the first paragraph of the Results section that the BG505 clade A Env model used in our simulations lacks the N618 and N625 N-linked glycans (see page 6, lines 160-165). We further clarify that these two gp41 glycans are present in other clades or Env constructs different from ours, consistent with available sequence and glycoanalytic data reported in Cao et al. 2018.

In addition, we have expanded the Discussion section to explicitly address the absence of N618 and N625 in our simulations and stated that their presence could influence Env–membrane interactions and potentially affect tilting dynamics (see page 21, lines 487-491). We emphasize that, while our conclusions regarding the role of N88 and N611 in modulating Env tilting are robust within the clade A background and the specific glycoform modeled here, the presence of additional gp41 N-linked glycans in other strains may quantitatively alter the magnitude or propensity of tilting, a question that warrants future investigation.

We believe these additions substantially improve transparency and appropriately contextualize the scope and generalizability of our findings.

Reviewer 4

- *I appreciate the authors' clarification that glycan-specific functional experiments, especially at sites such as N88 and N611, would represent a substantial and independent line of work and are beyond the scope of this manuscript. That said, I would like to stress that such experiments will ultimately be essential for placing the proposed glycan-mediated mechanism on firm experimental and functional footing.*

Response: We thank the reviewer for this thoughtful and balanced assessment. We fully agree that glycan-specific functional experiments, particularly at sites such as N88 and N611, will ultimately be essential to place the proposed glycan-mediated mechanism on firm experimental footing, and we view such studies as an important direction for future investigation.

- *The current cryo-ET data support the global tilting behaviour of Env, but due to the relatively low resolution of the subtomogram averages, they cannot resolve individual glycans or detailed MPER/TMD conformations. As a result, the specific roles of N88 and N611 still remain simulation-derived and hypothesis-generating, rather than functionally demonstrated.*

Response: We agree that our cryo-ET data robustly support the existence and extent of Env tilting in a native membrane context, and yet that the current resolution of subtomogram averages does not permit direct visualization of individual glycans or detailed MPER/TMD conformations. We also fully acknowledge and appreciate that the specific mechanistic role proposed for N88 and N611 is computationally derived from all-atom MD simulations rather than directly resolved experimentally. In response to this comment and the previous comment, we have now explicitly clarified this nuance in the Discussion section (page 21, lines 481–485), where we also emphasize the need for future experiments to corroborate the proposed glycan-mediated effects.

We thank both reviewers for their excellent engagement and work to help revise and improve our paper!

Point by point Response to Reviewers

Reviewer 1

- *In The authors have responded robustly to my last set of comments, with clear caveat in the first paragraph to the results highlighting the absence of glycans N618 and N625 along with added discussion relating to the presence or absence of these two glycans. With these added edits, readers are now appropriately informed of the limitations of the clade a BG505 model.*

Response: We thank the reviewer for their positive feedback.

Reviewer 4

- *The authors have addressed my previous comments by noting that glycan-specific functional experiments, particularly at sites such as N88 and N611, are beyond the scope of the present study and are deferred to future work. While the data support the observed global Env tilting behaviour, the proposed glycan-mediated mechanisms remain, as previously noted, largely based on simulations. Overall, the authors' acknowledgement of the limitations of MD simulations and the need for future experimental validation helps to place the mechanistic conclusions in an appropriate context.*

Response: We thank the reviewer for their positive feedback.
